# Glycyrrhizin suppresses epithelial-mesenchymal transition by inhibiting high-mobility group box1 via the TGF-*β*1/Smad2/3 pathway in lung epithelial cells

**DOI:** 10.7717/peerj.8514

**Published:** 2020-02-03

**Authors:** Yanni Gui, Jian Sun, Wenjie You, Yuanhui Wei, Han Tian, Shujuan Jiang

**Affiliations:** 1 Department of Respiratory and Critical Care Medicine, Shandong Provincial Hospital Affiliated to Shandong University, Jinan, Shandong, China; 2 Cheeloo Collage of Medicine, Shandong University, Jinan, Shandong, China; 3 Department of Respiratory and Critical Care Medicine, Shandong Provincial Hospital Affiliated to Shandong First Medical University, Jinan, Shandong, China

**Keywords:** Glycyrrhizin, High-mobility group box1, Epithelial-mesenchymal transition, TGF-β1, Chronic airway diseases, Lung epithelial cells

## Abstract

**Background:**

Epithelial-mesenchymal transition (EMT) plays an important role in fibrosis, chronic inflammation, tumor metastasis, etc. Glycyrrhizin, an active component extracted from licorice plant, has been reported to treat a variety of inflammatory reactions through inhibiting high-mobility group box1 (HMGB1), which has been suggested to be a significant mediator in EMT process. However, whether glycyrrhizin affects the EMT process or not remains unclear.

**Methods:**

Human alveolar epithelial cell line A549 and normal human bronchial epithelial cell line BEAS-2B were treated with extrinsic TGF-*β*1 to induce EMT. Elisa was used to detect HMGB1 concentrations in cell supernatant. RNA interference and lentivirus infection experiments were performed to investigate the involvement of HMGB1 in EMT process. Cell Counting Kit-8 (CCK-8) was used to detect the viability of A549 and BEAS-2B cells treated with glycyrrhizin. Finally, the effects of glycyrrhizin on EMT changes, as well as the underlying mechanisms, were evaluated via Western blot, immunofluorescence and transwell assays.

**Results:**

Our results showed that HMGB1 expression was increased by TGF-*β*1, and knockdown of HMGB1 expression reversed TGF-*β*1-induced EMT in A549 and BEAS-2B cells. Ectopic HMGB1 expression or TGF-*β*1 treatment caused a significant increase in HMGB1 release. Notably, we found that glycyrrhizin treatment effectively suppressed TGF-*β*1-induced EMT process by inhibiting HMGB1. Also, glycyrrhizin significantly inhibited the migration of both A549 and BEAS-2B cells promoted by TGF-*β*1. Mechanistically, HMGB1 overexpression could activate Smad2/3 signaling in A549 and BEAS-2B cells. Glycyrrhizin significantly blocked the phosphorylation of Smad2/3 stimulated either by TGF-*β*1 or by ectopic HMGB1 in A549 and BEAS-2B cells.

**Conclusions:**

HMGB1 is a vital mediator of EMT changes induced by TGF-*β*1 in lung epithelial cells. Importantly, glycyrrhizin can effectively block Smad2/3 signaling pathway through inhibiting HMGB1, thereby suppressing the EMT progress.

## Introduction

Epithelial-mesenchymal transformation (EMT), a complex process in which epithelial cells are transformed into mesenchymal phenotype cells through specific procedures, accompanied by a series of cytoskeletal and morphological changes (Hay, 1995). In the process of EMT, epithelial cell markers including E-cadherin and Claudin are down-regulated, while mesenchymal cell markers such as vimentin and N-cadherin are up-regulated. Epithelial cells lose their intercellular junctions and apical-basal polarity, showing a fusiform cell morphology, finally acquiring the ability of migration and invasion (Acloque et al., 2009; Kalluri & Weinberg, 2009). Studies have found that EMT not only participates in the process of embryonic development, but also plays an important role in chronic inflammation, tissue reconstruction, cancer metastasis and a variety of fibrosis diseases (Nieto et al., 2016; Schmidt, Sundaram & Flaherty, 2009; Tam & Weinberg, 2013; Vaughan & Chapman, 2013). Multiple signaling pathways are involved in the EMT progression, among which transforming growth factor-*β*1(TGF-*β*1) family signaling plays a predominant role (Lamouille, Xu & Derynck, 2014).

High-mobility group box1 (HMGB1), a highly conserved nonhistone chromosomal protein in the nucleus, mainly stabilizes the structure of nucleosomes and regulates gene transcription, which can be released when stressed (Kang et al., 2014). Extracellular HMGB1 activates the downstream signaling pathway by binding to its corresponding functional receptors including the receptor of advanced glycation end products (RAGE) and toll-like receptors (TLR2 and TLR4 etc.), concomitantly regulating the immune response and promoting the progression of inflammation, tissue and organ fibrosis (Martinotti, Patrone & Ranzato, 2015; Webster et al., 2019; Wu et al., 2013). In addition, HMGB1 can induce EMT in some tumors, such as prostate cancer, hypopharyngeal carcinoma and non-small cell lung cancer (NSCLC), promoting cell migration and invasion (Li et al., 2017; Meng et al., 2019; Zhang et al., 2018b).

Glycyrrhizin, the main active component obtained from licorice roots, has many pharmacological and biological functions such as protecting liver cells, anti-inflammation, anti-virus, immunomodulation, has been widely applied in the treatment of clinically related hepatic diseases (Dastagir & Rizvi, 2016). Glycyrrhizin is a natural inhibitor of HMGB1, which can bind directly to HMGB1, interacting with two shallow concave surfaces formed by the two arms of both HMG boxes (Mollica et al., 2007). Glycyrrhizin can inhibit the chemotactic and mitogenic functions of HMGB1 and reduce its protein activity, thereby inhibiting the inflammatory response caused by HMGB1 overexpression (Kim, Kim & Chang, 2015). Studies have confirmed that glycyrrhizin has a certain protective effect on lung, which can reduce the release of various inflammatory cytokines to alleviate the lung injury (Li et al., 2018; Tsao & Yin, 2015; Zheng et al., 2019) and suppress the growth of lung adenocarcinoma cells (Wu et al., 2018). However, whether glycyrrhizin has a regulatory effect on EMT is still unclear. Therefore, it is of great significance to explore the effect of glycyrrhizin on EMT of epithelial cells, and to study the relevant pathways and mechanism of this process, providing certain guidance for clinical treatment of EMT-related diseases.

In this study, we found the crosstalk between HMGB1 and TGF-*β*1/Smad2/3 pathway is involved in the EMT process in lung epithelial cells. Our findings indicated that glycyrrhizin suppresses EMT by inhibiting HMGB1 and blocking the downstream Smad2/3 signaling pathway.

## Materials & Methods

### Cell culture

Human lung alveolar epithelial cell line A549 and normal bronchial epithelial cell line BEAS-2B were obtained from the American Type Culture Collection (Rockville, MD, USA). All cell lines were maintained in 1,640 medium supplemented with 10% fetal bovine serum (FBS) and 1% antibiotics (100 U/mL penicillin and 100 µg/mL streptomycin), at 37 °C in a humidified incubator with 5% CO_2_. Cells were passaged no more than 15 times after thawing. TGF-*β*1 (Humanzyme, Chicago, IL, USA) was prepared with a buffer containing 0.1% bovine serum albumin (BSA) and the final concentration used was 5ng/ml (Lin et al., 2018; Tan & Cheng, 2018). Glycyrrhizin (Sigma-Aldrich, St. Louis, MO, USA) was dissolved in DMSO and then diluted with 1,640 medium into different concentrations. Cells were cultured for 24 h in 1,640 containing TGF-*β*1(5 ng/ml), with different concentration of glycyrrhizin pretreatment for 2 h.

### Cell viability assay

Cells were seeded into 96-well plate (2 × 10^3^ cells per well) in triplicate and treated with glycyrrhizin (ranged from 25 to 400 µM) or vehicle for 24 or 48 h. Cell Counting Kit-8 (Dojindo, Tokoyo, Japan) was used to detected the cell proliferation rate of each group and 10 µl WST-8 was added into each well. After incubation for 1 h, the optical density (OD) of each hole was measured at 450 nm using an enzyme standard instrument.

### Elisa

Cells were seeded into 6-well plate with a density of 2.0 × 10^4^ per well. Cell supernatant was obtained after centrifugation. The concentration of HMGB1 in cell supernatant was determined with human HMGB1 ELISA kit (Elabscience, Wuhan, China) according to the manufacturer’s instruction. All the samples were analyzed in triplicate. The concentrations of HMGB1 in the samples were determined from standard curve.

### Transfection

Cells were seeded in a 6-well plate with a density of 1.0 × 10^4^ per well. Cells were transfected with siRNAs (Genomeditech, Shanghai, China) using Lipofectamine 2000 (Invitrogen, Carlsbad, CA, USA) or infected with prepared HMGB1 virus (Genechem, ShangHai, China) at different MOI respectively in accordance with the manufacturers’ instructions. The transfection or infection effects were detected by RT-PCR and Western blot. The siRNA sequences are listed as follows: HMGB1-siRNA-1: sense 5′-GGAGAGAUGUGGAAUAACAtt-3′and antisense 5′-UGUUAUUCCACAUCUCUCCtt-3′; HMGB1-siRNA-2: sense 5′-CCAUUGGUGAUGUUGCGAAtt-3′and antisense 5′-UUCGCAACAUCACCAAUGG-3′.

### RT-PCR analysis

Total RNA from cultured A549 and BEAS-2B cells was extracted using TRIzol reagent (TaKaRa, Shiga, Japan) according to the manufacturer’s instructions. 1 mg RNA samples were used for cDNA synthesis. Amplification was conducted using a SYBR Green I PCR kit (TaKaRa, Shiga, Japan). The cycling conditions were as follows: 95 °C for 30s, followed by 40 cycles at 95 °C for 5 s and 60 °C for 20s. The relative expression levels of HMGB1 mRNA were normalized to human *β*-actin expression. The transcription primers are shown as follows:

HMGB1: forward, 5′-GAGAGGCAAAATGTCATCAT-3′, reverse, 5′- GGGATCCTTGAACTTCTTTT-3′;

*β*-actin: forward, 5′-AGTTGCGTTACACCCTTTCTTG-3′, reverse, 5′- CACCTTCACCGTTCCAGTTTT-3′.

### Western blotting

Collected cells were lysed in radioimmunoprecipitation (RIPA) buffer (Beyotime, Shanghai, China) containing protease inhibitors to extract the protein effectively. Protein concentrations were detected by BCA assay kit (Beyotime, Shanghai, China). A 40 µg of protein samples were electrophoresed on 10% SDS polyacrylamide gel and resolved to PVDF membrane. Non-specific binding sites were blocked at room temperature for 1 h by 5% non-fat milk dissolved in Tris buffered saline (TBS) containing 0.1% Tween20 or blocking solution containing 1% BSA. Then the membranes were incubated overnight at 4 °C with the primary antibodies including GAPDH (Proteintech, Wuhan, China), E-cadherin, p-Smad2 (both from Cell Signaling Technology, Danvers, MA, USA), vimentin, HMGB1, TGF-*β*1, p-Smad3 (all from Abcam, Cambridge, UK). Gray values for each protein band were analyzed by Image J analysis software.

### Immunofluorescence analysis

Cells were seeded onto glass slides at a density of 8 × 10^3^ cells/ml, followed the incubation as above. Wash the slides with phosphate-buffered saline (PBS), fixed the cells with 4% paraformaldehyde at room temperature for 30 min, and then permeabilized them in 0.1% Triton X-100 for 10min. After being blocked with the goat serum for 1 h, cells were incubated with primary antibody (1:150) overnight at 4 °C. The next day, the slides were washed for three times and incubated with secondary antibody (1:200) for 1 h at room temperature in dark. PBST (PBS containing 0.1% Teen20) were used to washed away the remaining secondary antibodies and then the slides were mounted in antifade solution with DAPI (Solarbio, Beijing, China). Images were captured with an Olympus BX51 micro-scope (Japan).

### Cell migration assay

The incubated cells (1 × 10^4^cells/well) were plated in Matrigel-coated Transwell chambers (Corning, New York, NY, USA) with 8 µm pore size polycarbonate microporous membranes. 200 µL serum-free medium was added into the upper chamber while for 600 µL medium with 10% FBS was added into the lower chamber. After incubation for 24 h, the cells migrated the lower surface were fixed with 4% paraformaldehyde for 30 min and stained with 0.1% Hematoxylin for 15 min. Then, the invading cells were photographed by the fluorescence microscope and counted by Image J software.

### Statistical analysis

All results were performed at least three separate experiments. Data were analyzed using two-tailed unpaired *t*-tests with SPSS version23.0 (Chicago, IL, USA) and Graphpad Prism 7 (Graphpad Software, San Diego, CA, USA) and were shown as mean ± standard deviation (SD). Difference of *P* < 0.05 was considered statistically significant.

## Results

### Knockdown of HMGB1 expression reversed the TGF-*β*1-induced EMT in A549 and BEAS-2B cells

**Figure 1:**
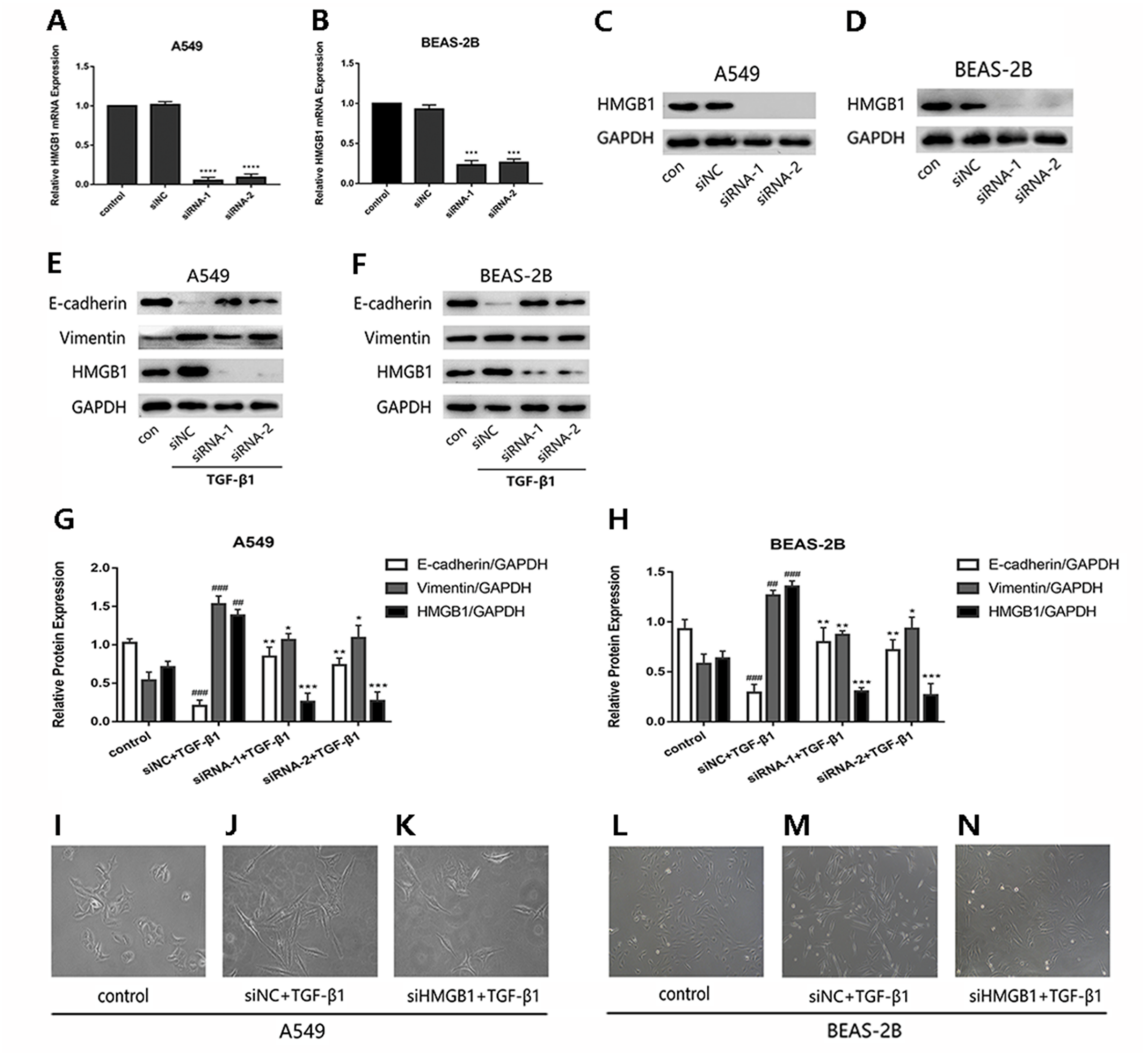
Knock down of HMGB1 reversed the TGF-*β*1-induced EMT in airway epithelial cells. (A, B) A549 and BEAS-2B cells were transfected with siRNA (50 nM) for 48 h, the mRNA expression of HMGB1 and *β*-actin were detected using RT-PCR. (C, D) The protein expression of HMGB1 were detected by Western blot after siRNA transfection. (E, F) The siRNA-transfected epithelial cells were treated with TGF- *β*1 (5 ng/ml) for 24 h, and E-cadherin, Vimentin and HMGB1 expression were detected using Western blot. (G, H) Statistical analysis of the relative expression of E-cadherin, Vimentin and HMGB1 in control and TGF-*β*1 groups. Data were normalized to GAPDH expression. ^##^*P* < 0.01, ^###^*P* < 0.001 compared with the value of control group, **P < 0.01, ***P < 0.001 compared with the value of siNC+TGF-*β*1 group. Data were the representatives of three independent experiments. (I–N) Morphological characteristics of each group were observed under 40× microscope.

To investigate whether HMGB1was involved in the EMT process induced by TGF-*β*1 in A549 and BEAS-2B cells, HMGB1 gene was knocked down by siHMGB1(siRNA-1 and siRNA-2) transfection. The mRNA level of HMGB1 was reduced (Figs. 1A and 1B), and the protein expression of HMGB1 was decreased significantly (Figs. 1C and 1D). As shown in Figs. 1E–1H, after TGF-*β*1 treatment for 24 h, the expression of E-cadherin was increased in HMGB1-silenced cells, while the expression of vimentin was decreased relatively in HMGB1-silenced cells compared with the control. Mesenchymal characteristics can be observed in TGF-*β*1 treated cells under the microscope, while HMGB1 knockdown slowed down the TGF-*β*1-induced morphological change in A549 and BEAS-2B cells (Figs. 1I–1N). Therefore, we draw the conclusion that HMGB1 was involved in TGF-*β*1-mediated EMT in lung epithelial cells.

### The lentivirus-mediated HMGB1 overexpression in A549 and BEAS-2B cells induced EMT, which can be inhibited by glycyrrhizin

**Figure 2:**
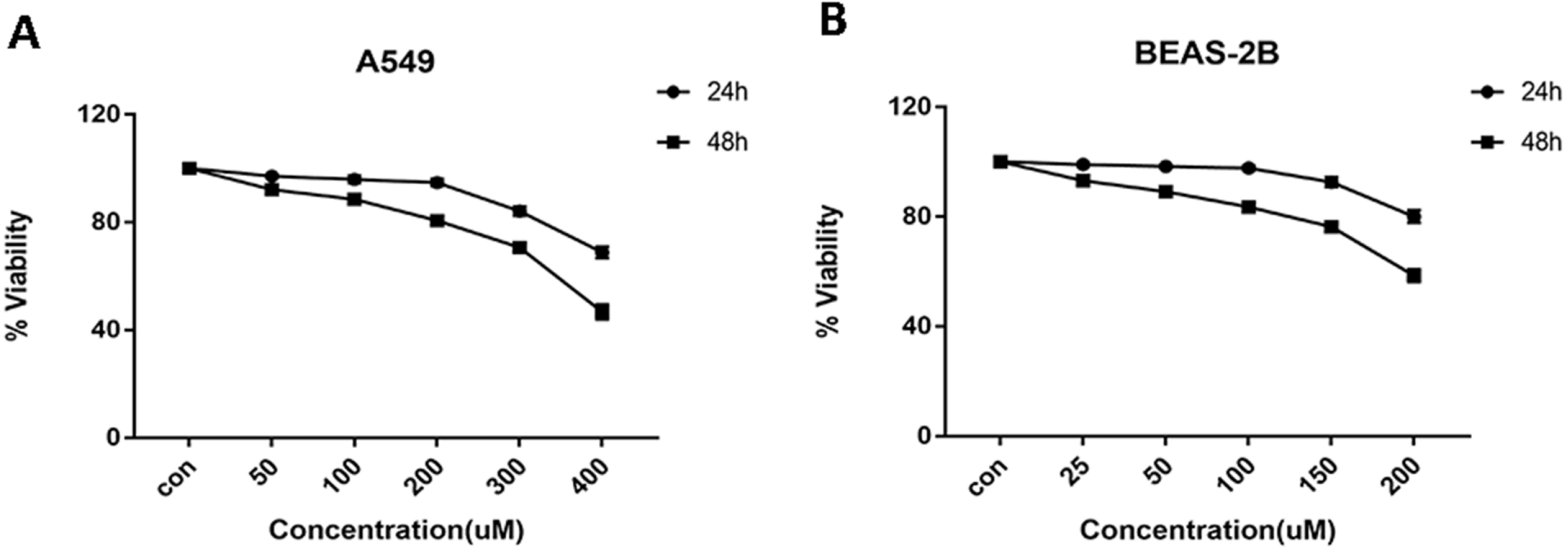
Cell viability of A549 and BEAS-2B cells treated with different concentrations of glycyrrhizin. (A) A549 cells were treated with glycyrrhizin at the concentrations of 50 µM, 100 µM, 200 µM respectively for 24 h and 48 h. (B) BEAS-2B cells were treated with glycyrrhizin at the concentrations of 25 µM, 50 µM, 100 µM respectively for 24 h and 48 h. Cell viability assays were performed to determine the viability of cells with various concentrations glycyrrhizin treatment.

Cells were treated with glycyrrhizin at different concentrations respectively for 24 h and 48 h to explore the appropriate drug treatments. And when treated for 24 h, there was no significant difference in cell viability at the concentrations of 50 µM, 100 µM, 200 µM for A549 cells (Table 1) and 25 µM, 50 µM, 100 µM for BEAS-2B cells (Table 2). As drug concentrations continued to rise, the cell viability had decreased significantly. Meanwhile, glycyrrhizin treatment for 48 h led to obvious reduction in cell viability at all concentrations (Fig. 2).

**Table 1:** Viability of A549 treated with various concentrations of glycyrrhizin 24 h ($\overline{\mathrm{X}}$± SD, *n* = 3). The statistical method used is unpaired *t*-test. Data were expressed as mean ± SD, *n* = 3.

concentration (µM)	0	50	100	200	300	400
viability (IR, %)	100 ± 0	92.97 ± 3.42	89.73 ± 4.17	85.48 ± 5.46	69.84 ± 4.91	60.50 ± 5.77
*p* value		0.1089	0.0695	0.0563	0.0036	0.0024

**Table 2:** Viability of BEAS-2B treated with various concentrations of glycyrrhizin 24 h ($\overline{\mathrm{X}}$± SD, *n* = 3). The statistical method used is unpaired *t*-test. Data were expressed as mean ± SD, *n* = 3.

concentration (µM)	0	25	50	100	150	200
Viability (IR, %)	100 ± 0	95.84 ± 2.64	92.19 ± 3.80	86.41 ± 5.48	78.35 ± 4.87	71.07 ± 5.44
*p* value		0.1898	0.1095	0.0684	0.0113	0.0060

**Figure 3:**
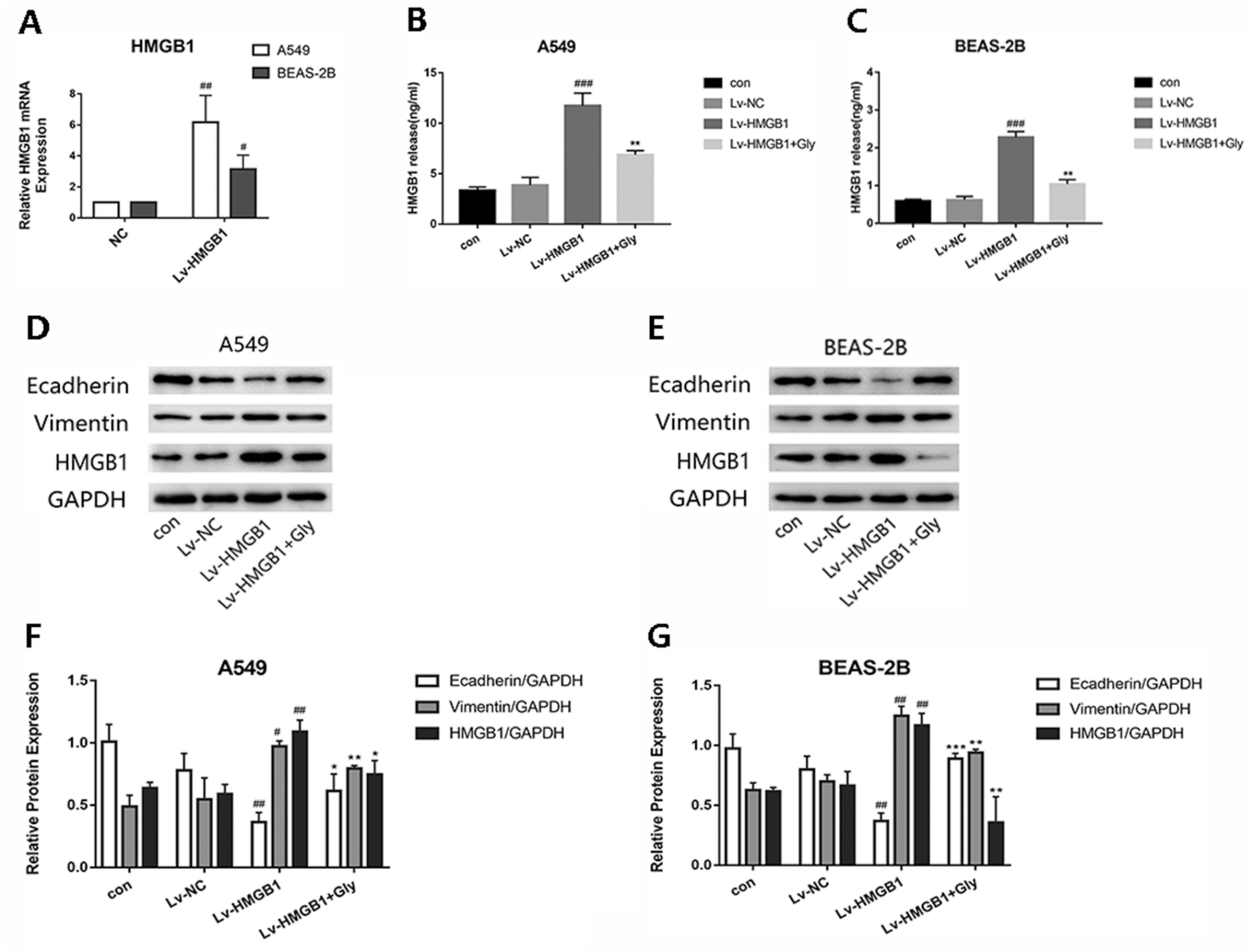
The lentivirus-mediated HMGB1 overexpression in A549 and BEAS-2B cells induced EMT, which can be inhibited by glycyrrhizin. Transfected the lung epithelial cells with lentivirus, then treated A549 cells with 100 µM glycyrrhizin and treated BEAS-2B cells with 50 µM glycyrrhizin for 24 h. (A) The relative HMGB1 mRNA expression was detected by RT-PCR to verify the transfection efficiency. Data were normalized to *β*-actin expression. (B, C) The concentration of HMGB1 in control, Lv-NC, Lv-HMGB1 and Lv-HMGB1+Glycyrrhizin groups were detected by Elisa. (D, E) The E-cadherin, Vimentin and HMGB1 expression of cells was detected using Western blotting. (F, G) Statistical analysis of the relative expression of E-cadherin, Vimentin and HMGB1. ^#^*P* < 0.05, ^##^*P* < 0.01 compared with the value of Lv-NC group, *P < 0.05, **P < 0.01, ***P < 0.001 compared with the value of Lv-HMGB1 group.

Infected A549 and BEAS-2B cells with Lv-HMGB1, then the HMGB1 mRNA expression level determined by real-time PCR was significantly increased (Fig. 3A). We found that the level of HMGB1 in cell supernatant detected by Elisa kit were elevated significantly compared with the control group, which could be reduced by glycyrrhizin treatment (Figs. 3B and 3C). In the two epithelial cells, the expression of E-cadherin in Lv-HMGB1 groups was downregulated, while the expression of vimentin was upregulated compared with the control groups, indicating that HMGB1 overexpression promoted the EMT progress. And treatment with glycyrrhizin rescued E-cadherin expression and suppressed vimentin expression (Figs. 3D–3G). Therefore, glycyrrhizin inhibited the EMT induced by HMGB1 overexpression.

### Glycyrrhizin suppressed the TGF-*β*1-induced EMT process by inhibiting HMGB1

**Figure 4:**
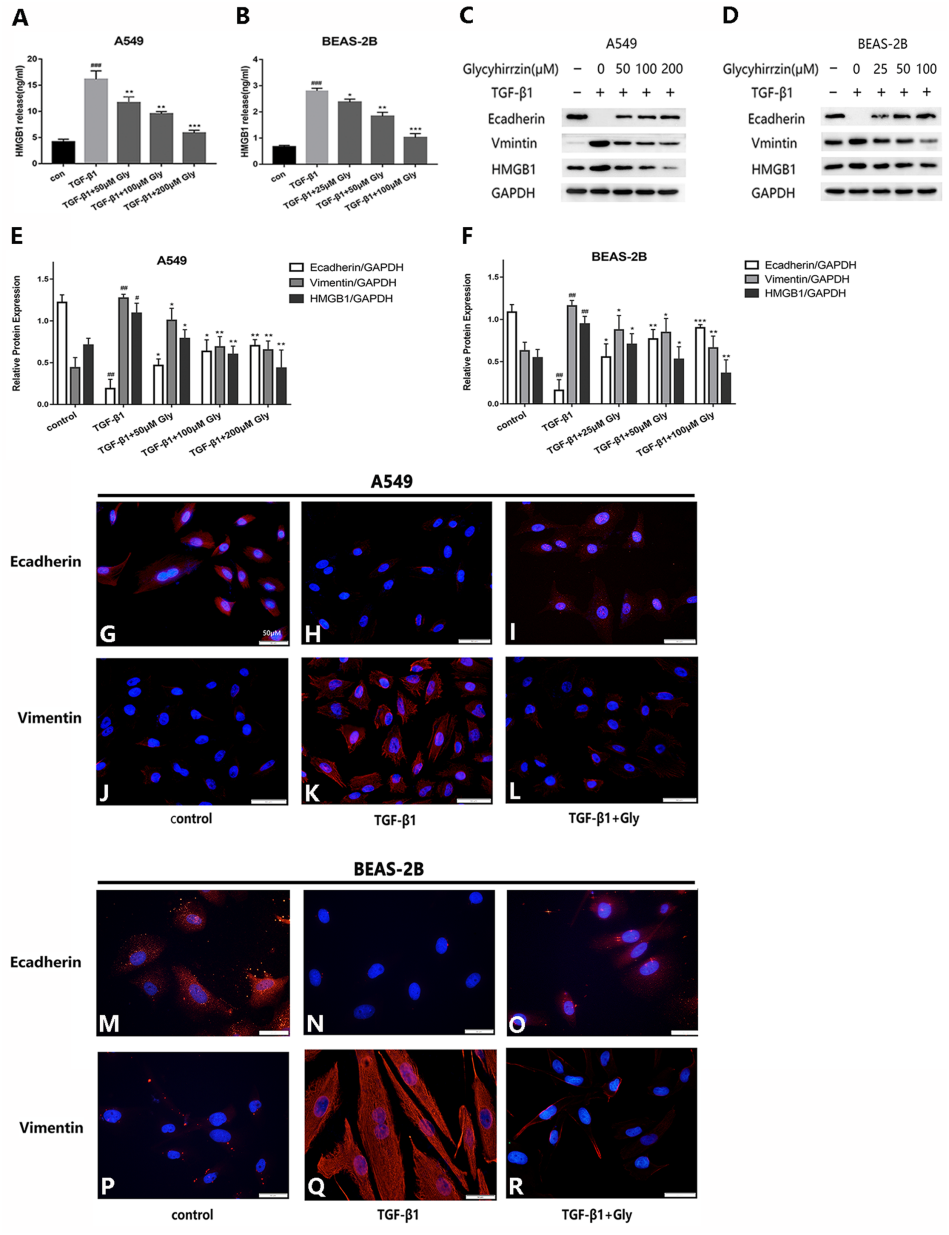
Glycyrrhizin suppressed the TGF-*β*1-induced EMT process by inhibiting HMGB1. Pretreated A549 cells (50 µM, 100 µM, 200 µM) and BEAS-2B cells (25 µM, 50 µM, 100 µM) with glycyrrhizin for 2 h, then stimulated the cells with 5 ng/ml TGF-*β*1 for 24 h. (A, B) Elisa was performed to detect the HMGB1 concentration in cell supernatant. (C, D) Western blot was used to detect the expression of E-cadherin, Vimentin, and HMGB1. (E, F) Statistical analysis of the expression of E-cadherin, Vimentin and HMGB1 compared to GAPDH. ^#^*P* < 0.05, ^##^*P* < 0.01 compared with the value of control group, *P < 0.05, **P < 0.01, ***P < 0.001 compared with the value of TGF-*β*1 group. (G-R) A549 and BEAS-2B cells were stained with DAPI (blue, nuclear stain) and antibodies to E-cadherin or Vimentin (red), and confocal images were acquired at 40× magnification. All experiments were performed in three independent experiments.

The two cell lines were exposed to TGF-*β*1 (5 ng/ml) for 24 h, with or without different concentrations of glycyrrhizin pretreatment for 2 h. We detected the high level of HMGB1 in cell supernatant in TGF-*β*1 treated group with Elisa. It indicated that TGF-*β*1 triggered HMGB1 release to supernatant, while glycyrrhizin treatment could reduce it (Figs. 4A and 4B). As examined by western blotting, E-cadherin was downregulated and vimentin was upregulated, and the HMGB1 expression was increased by TGF- *β*1 significantly. With glycyrrhizin pretreatment, the expression of E-cadherin was rescued and the expression of vimentin was downregulated (Figs. 4C–4F). By immunofluorescence analysis, the fluorescence signal of E-cadherin in TGF-*β*1 group was almost nonexistent, while the vimentin signal was enhanced. Whereas in TGF-*β*1+Glycyrrhizin group, the level of E-cadherin was increased and the vimentin was decreased obviously (Figs. 4G–4R). These findings showed that glycyrrhizin suppressed the TGF-*β*1-induced EMT by inhibiting HMGB1, and the effects were in a concentration-dependent manner.

### Glycyrrhizin inhibited cell migration promoted by TGF-*β*1

**Figure 5:**
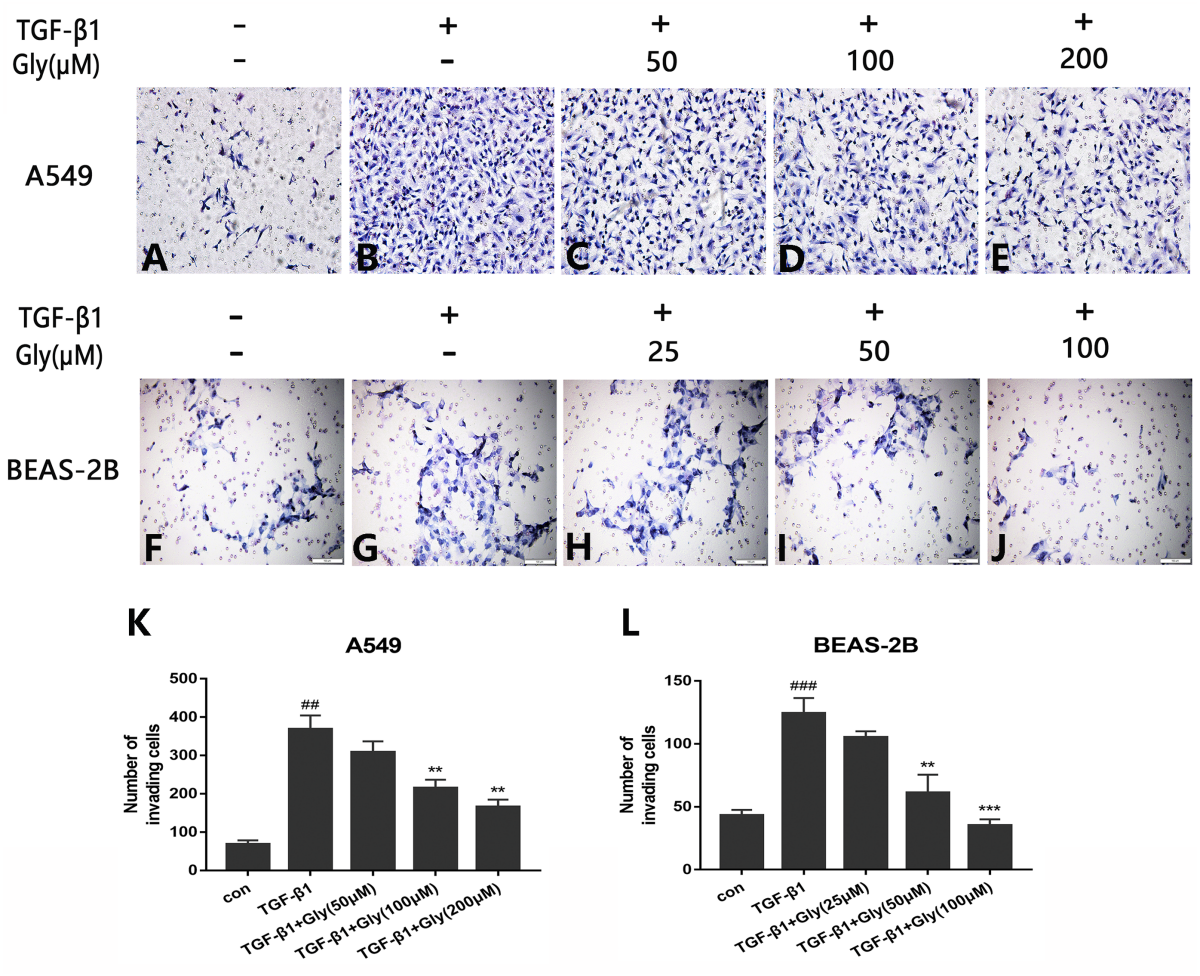
Effect of glycyrrhizin on TGF-*β*1-mediated cell migration of A549 and BEAS-2B cells. (A–J) Cell migration capacity were detected by transwell migration assays. The number of migrated cells were counted by ImageJ analysis software. (K, L) Analyze the number of migrated cells according to three independent repeated experiments. ^##^*P* < 0.01, ^###^*P* < 0.001, **P < 0.01, ***P < 0.001.

The number of migrating cells was increased in the TGF-*β*1 group than in the control group, indicating that TGF-*β*1 promoted cell migration capacity of A549 and BEAS-2B cells. The number of migrating cells in glycyrrhizin pretreated groups was lower than the TGF-*β*1 group, indicating that glycyrrhizin inhibited cell migration promoted by TGF-*β*1 (Figs. 5A–5J). Moreover, as the concentration of glycyrrhizin rose, the number of migrating cells decreased (Figs. 5K and 5L).

### Glycyrrhizin blocked the TGF-*β*1/Smad2/3 pathway by inhibiting HMGB1

**Figure 6:**
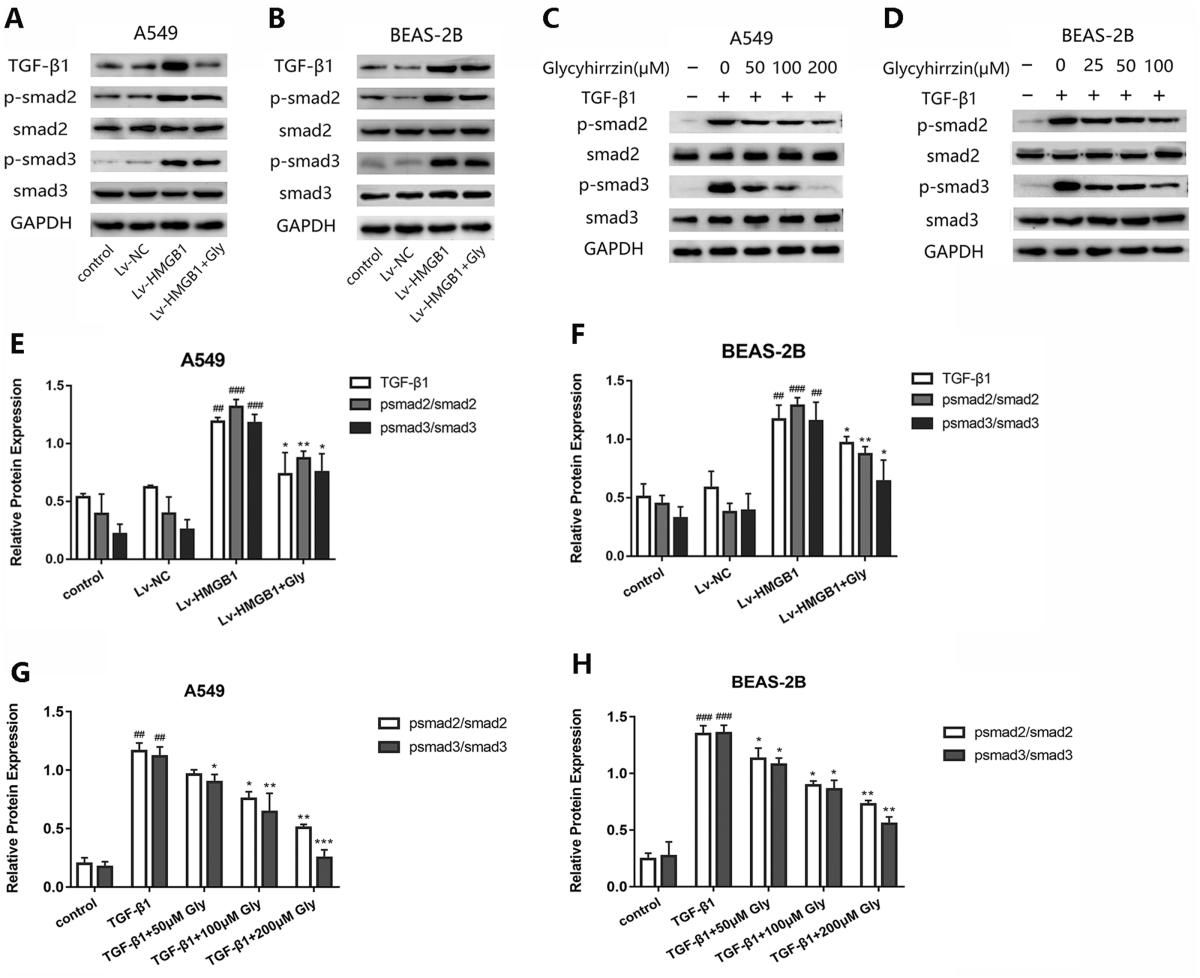
Glycyrrhizin blocked the TGF-*β*1/Smads pathway by inhibiting HMGB1. (A, B) Western blot was used to detect the protein level of TGF-*β*1, Smad2, p-Smad2, Smad3, p-Smad3 of cells in the control, Lv-NC, Lv-HMGB1, Lv-HMGB1+glycyrrhizin groups. (C, D) Western blot analysis was used to detect the protein expression of Smad2, p-Smad2, Smad3, p-Smad3 of cells stimulated by 5 ng/ml TGF-*β*1 with or without various concentrations of glycyrrhizin pretreatment. (E, F) Statistical analysis of the relative expression of TGF-*β*1/GAPDH, p-Smad2/Smad2, and p-Smad3/Smad3. ^##^*P* < 0.01, ^###^*P* < 0.001 compared with the value of Lv-NC group, *P < 0.05, **P < 0.01, compared with the value of Lv-HMGB1 group. (G, H) Analyze the relative expression of p-Smad2/Smad2, p-Smad3/Smad3 according to the data of three independent repeated experiments. ^##^*P* < 0.01, ^###^*P* < 0.001 compared with the value of control group, *P < 0.05, **P < 0.01, ***P < 0.001 compared with the value of TGF-*β*1 group.

To investigate whether TGF-*β*1/Smad2/3 pathway was involved in the EMT process induced by overexpressed HMGB1, we tested the protein levels of TGF-*β*1, phospho-Smad2, phosphor-Smad3 and total Smad2/3 in A549 and BEAS-2B cells (Figs. 6A and 6B). All the levels of TGF-*β*1, phospho-Smad2 and phospho-Smad3 in HMGB1 overexpressed cells were higher than the normal group. The phosphorylation of Smad2/3 was promoted, indicating that the TGF-*β*1/Smad2/3 pathway was activated by HMGB1 overexpression. Moreover, glycyrrhizin treatment downregulated the expression of TGF- *β*1, p-Smad2/Smad2 and p-Smad3/Smad3, showing that glycyrrhizin could block the TGF-*β*1/Smad2/3 pathway activated by HMGB1 overexpression (Figs. 6E and 6F).

After stimulation by TGF-*β*1, the expressions of p-Smad2/Smad2 and p-Smad3/Smad3 were upregulated (Figs. 6C and 6D). However, in the groups with glycyrrhizin pretreatment, the phosphorylation of Smad2/3 was blocked in a concentration-dependent manner (Figs. 6G and 6H). These findings illustrated that glycyrrhizin suppressed the TGF-*β*1/Smad2/3 pathway by inhibiting HMGB1.

## Discussion

HMGB1 is a highly abundant protein that is widely expressed in a variety of human cells, especially in lung, such as lung epithelial cells, lung endothelial cells and alveolar macrophages (Ding, Cui & Liu, 2017; Hiraku et al., 2016). HMGB1 is normally located in the nucleus and the ratio of nuclear to cytoplasmic HMGB1 is about 30:1 (Kuehl, Salmond & Tran, 1984). As the most mobile protein in the nucleus, HMGB1 can translocate from the nuclei to the cytoplasm, and then is released into the extracellular space by immune cells, fibroblasts, or epithelial cells actively, following various stressors (Kang et al., 2014). HMGB1 release plays a critical role in stimulating the acute airway inflammatory responses (Wu et al., 2013), promoting the tissue repair and structural remodeling in chronic airway diseases (Lee et al., 2013), and accelerating the invasion and migration of lung tumors (Meng et al., 2019). Studies have found that HMGB1 bound to TLR4 and RAGE receptors, promoted the extracellular matrix synthesis and modulated cell–matrix adhesion to stimulate epithelial cells repair and restitution (Ojo et al., 2015). Inhibiting HMGB1 prevented the development of pulmonary fibrosis in rats induced by bleomycin (Zhang et al., 2015), also inhibited the EMT progress in NSCLC cells and weakened their invasion and migration abilities (Liu et al., 2017). In our study, we confirmed that the extracellular HMGB1 in the control group was at low levels. However, both HMGB1 overexpression and TGF-*β*1 treatment accelerated the transfer of HMGB1 from nucleus to cell supernatant, and then promoted the occurrence of EMT in human bronchial epithelial cell line BEAS-2B and the alveolar epithelial cell line A549.

HMGB1 can be involved in the process, in which TGF-*β*1 signaling pathway contributes to EMT. As a pleiotropic cytokine closely related to inflammation, repair, cell proliferation and apoptosis, transforming growth factor-*β*1(TGF-*β*1) has been shown to promote EMT through multiple signaling pathways (Luo et al., 2019). TGF- *β*1 can activate the tetrameric complex of T beta R I (TGFR1) and T beta R II(TGFR2), then the post-receptor sensors Smad2 and Smad3 are phosphorylated and combined with Smad4 to form a relatively stable trimeric complex, which can regulate the EMT process through synergistic action with the related transcription factors (Syed, 2016; Xu, Lamouille & Derynck, 2009). It has been proved that advanced glycation end products (AGEs) induced HMGB1 and promoted the connective tissue growth factor (CTGF) and TGF-*β*1 in renal epithelial HK-2 cells receptor for advanced glycation end products (RAGE)-dependently (Cheng et al., 2015), regulating the progress of renal fibrosis in diabetic nephropathy (Lynch et al., 2010). HMGB1 can regulate TGF-*β*1-induced EMT of FaDu hypopharyngeal carcinoma cells through activation of RAGE (Li et al., 2017). In our experiments, we found that knockdown of HMGB1 expression, the EMT progress induced by TGF-*β*1 was suppressed. We had detected the elevated expression of TGF-*β*1, p-Smad2 and p-Smad3 in the HMGB1 overexpression cells, indicating that HMGB1 overexpression promoted the phosphorylation of Samd2 and Smad3, thereby activating the TGF-*β*1/Smad2/3 signaling pathway. Moreover, TGF-*β*1 treatment upregulated the HMGB1 expression and promoted the HMGB1 release into cell supernatant, further confirming that there was a crosstalk between HMGB1 and TGF-*β*1 signaling in EMT.

In recent years, studies on glycyrrhizin are not only limited to its anti-inflammatory and antiviral effects, but also its therapeutic effects on fibrosis. Glycyrrhizin inhibited pro-fibrotic cytokine TGF-*β*1-mediated pathways, including Smad2, Smad3 as an active ingredient in astragalus saponins (Zhou et al., 2016), changed the pathological morphology and prevented the liver fibrosis induced by the bile duct ligation (BDL) model (Qu et al., 2015; Zhang et al., 2018a). Glycyrrhizin can inhibit the progression of bleomycin induced lung fibrosis in rats by inhibiting HMGB1 (Kida et al., 2018). The latest research conducted by Heng-Yu Chang et al. showed that in prostate cancer cells, glycyrrhizin may block the EMT process by modulating HMGB1 initiated novel signaling pathway through Cdc42, GSK-3*β*, Snail, and E-cadherin Kinases (Chang et al., 2019). To test whether glycyrrhizin could regulate the HMGB1-related EMT progress in lung epithelial cells, we chose the proper concentrations of glycyrrhizin hardly affected the growth of A549 and BEAS-2B cells by CCK-8 assays. We found that when the HMGB1-overexpressed epithelial cells were treated with glycyrrhizin, the HMGB1 release was reduced, and the EMT progress obtained a certain degree of inhibition, therefore we considered that glycyrrhizin can reverse the progress of EMT in lung epithelial cells by inhibiting HMGB1. Meanwhile, the migration capacity of the epithelial cells was significantly decreased by glycyrrhizin. In the following experiments, we found that with glycyrrhizin pretreatment, HMGB1 release was reduced, and the TGF-*β*1-induced EMT progress in the two cell lines were suppressed, along with the obstruction of the phosphorylation of Smad2 and Smad3.

EMT has been proved to be significantly associated with pulmonary fibrosis and lung cancers, providing a new target for clinical drug treatment work. But for now, there are still no specific drugs that can slow down or even reverse the EMT progress. Most previous studies on EMT have focused on the development and metastasis of tumors, however the key role of EMT in the occurrence and development of chronic airway disease should not be ignored. Glycyrrhizin is expected to be put into clinical trials in the adjuvant treatment of airway diseases for those therapeutic effects above. Our current work is at the cellular level, and we chose the appropriate concentrations of glycyrrhizin that did not affect cell viability. We need further animal experiments to verify the results of experiments *in vitro*. Clinical trials will be carried out if approved, notably, the adverse reactions of glycyrrhizin and the possible interactions with other drugs taken concurrently should be explored.

## Conclusions

Our research indicated that there was a crosstalk between HMGB1 and TGF-*β*1/Smad2/3 pathway related to EMT progress. In the alveolar epithelial cell line A549 and the human bronchial epithelial cell line BEAS-2B, knockdown of HMGB1 expression restrained the EMT process mediated by TGF-*β*1, while HMGB1 overexpression activated the TGF-*β*1/Smad2/3 pathway involved in EMT. As a pleiotropic inhibitor of HMGB1, we found glycyrrhizin can inhibit the EMT induced by HMGB1 overexpression. Further study showed that by inhibiting HMGB1 release, glycyrrhizin suppressed TGF-*β*1-induced EMT progress, blocked the TGF-*β*1/Smad2/3 pathway and inhibited the cells migration. These findings may provide certain guidance for clinical treatment of EMT-related chronic airway diseases.

##  Supplemental Information

10.7717/peerj.8514/supp-1 Supplemental Information 1 Western blots (Figs. 1, 3 and 4)

10.7717/peerj.8514/supp-2 Supplemental Information 2 Viability of cells detected by CCK-8 three times

10.7717/peerj.8514/supp-3 Supplemental Information 3 Results of RT-PCR for measuring the lentivirus transfection effects *n* = 3.

10.7717/peerj.8514/supp-4 Supplemental Information 4 Results of RT-PCR for measuring the siRNA transfection effects *n* = 3.

## Additional Information and Declarations

### Data Availability

The following information was supplied regarding data availability: The raw data is available in the Supplemental Files.
