# Supplementary figures and images for: Glycyrrhizin suppresses epithelial-mesenchymal transition by inhibiting high-mobility group box1 via the TGF-β1/Smad2/3 pathway in lung epithelial cells
Source: PeerJ. 2020 Feb 3;8:e8514. doi: 10.7717/peerj.8514 (PMC7003690; doi:10.7717/peerj.8514)

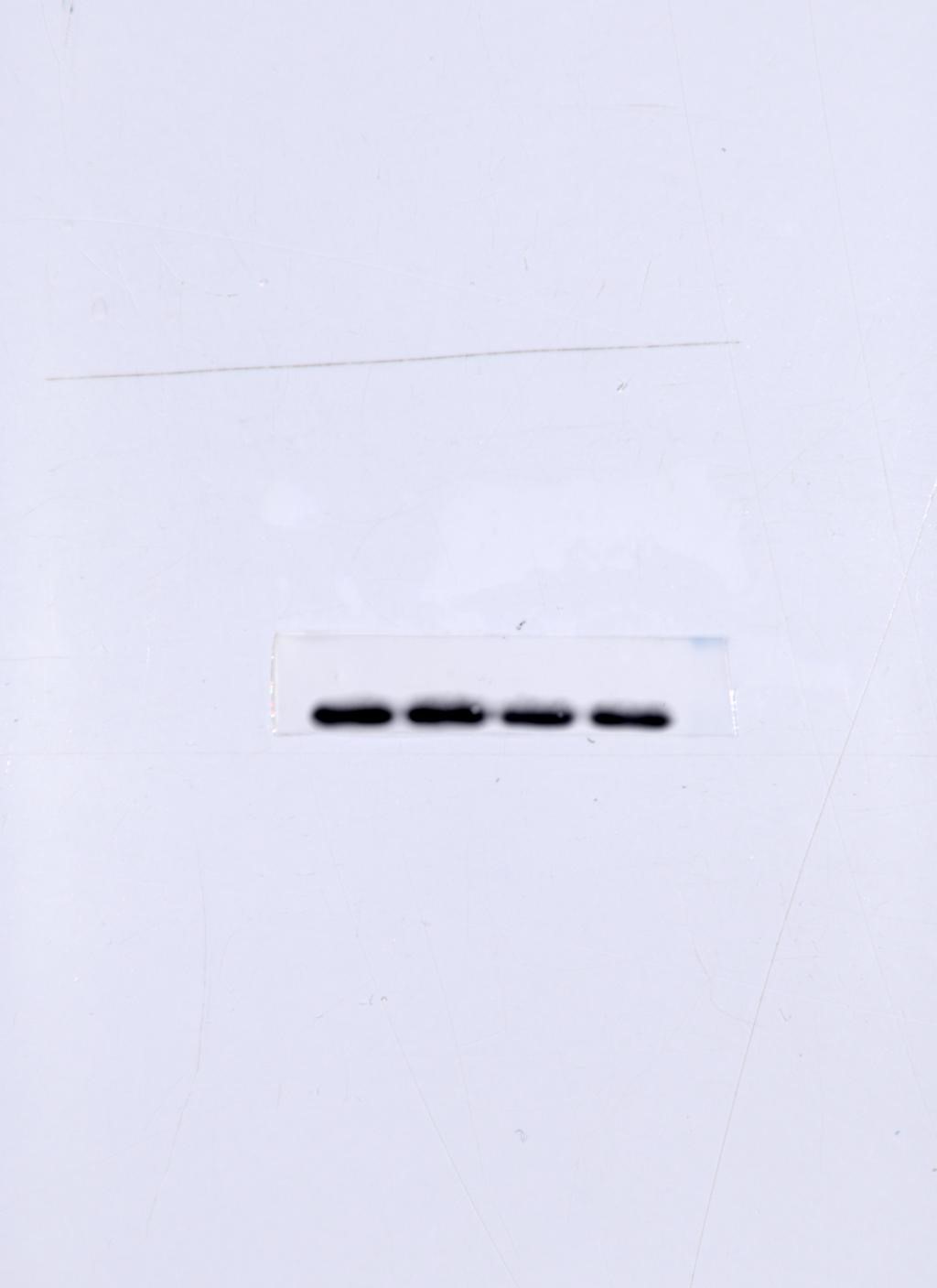

Supplement: Supplemental Information 1 [file peerj-08-8514-s001.zip › western blot/Figure1/Figure1B/A549/GAPDH.jpg]

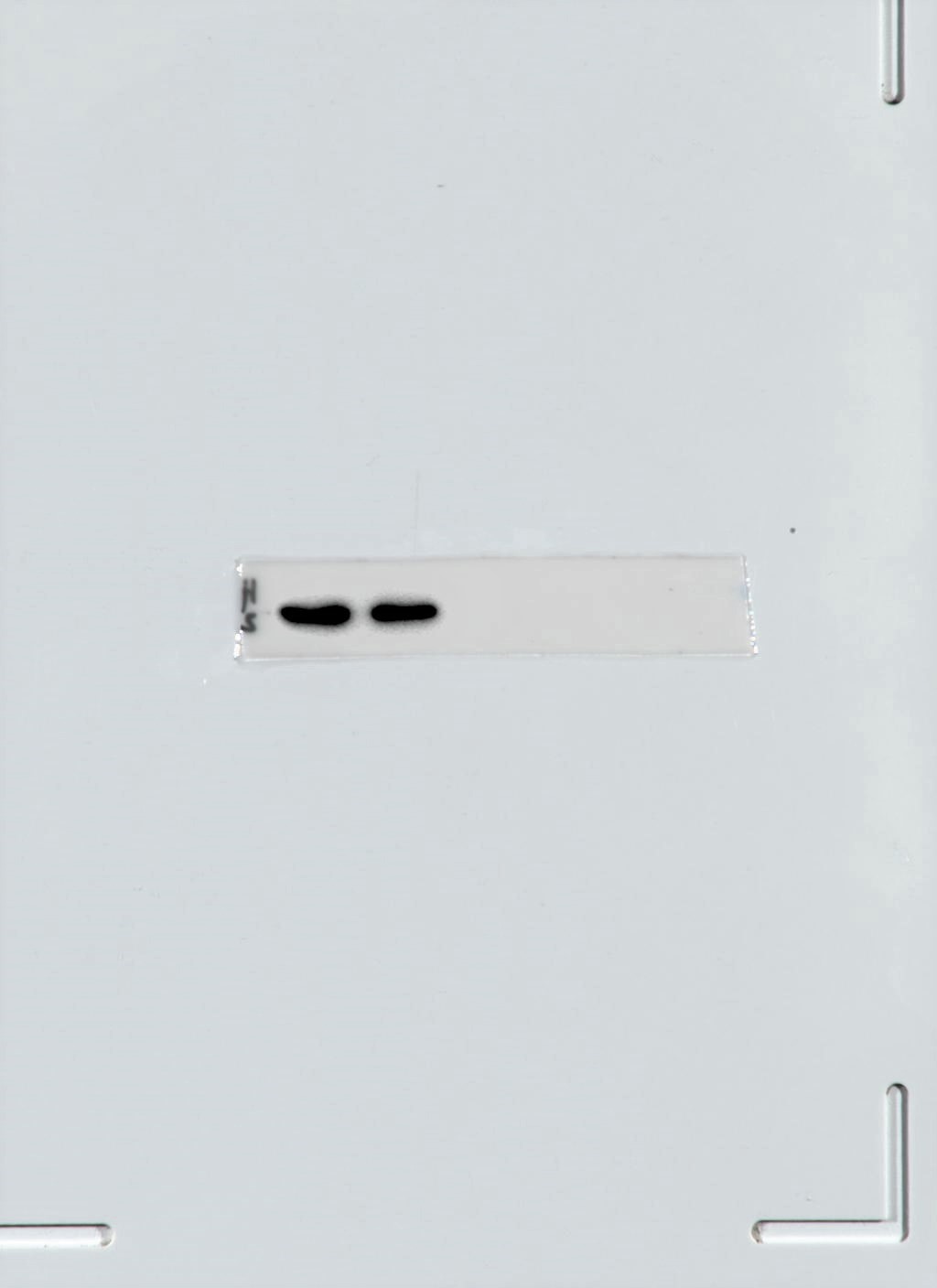

Supplement: Supplemental Information 1 [file peerj-08-8514-s001.zip › western blot/Figure1/Figure1B/A549/HMGB1.jpg]

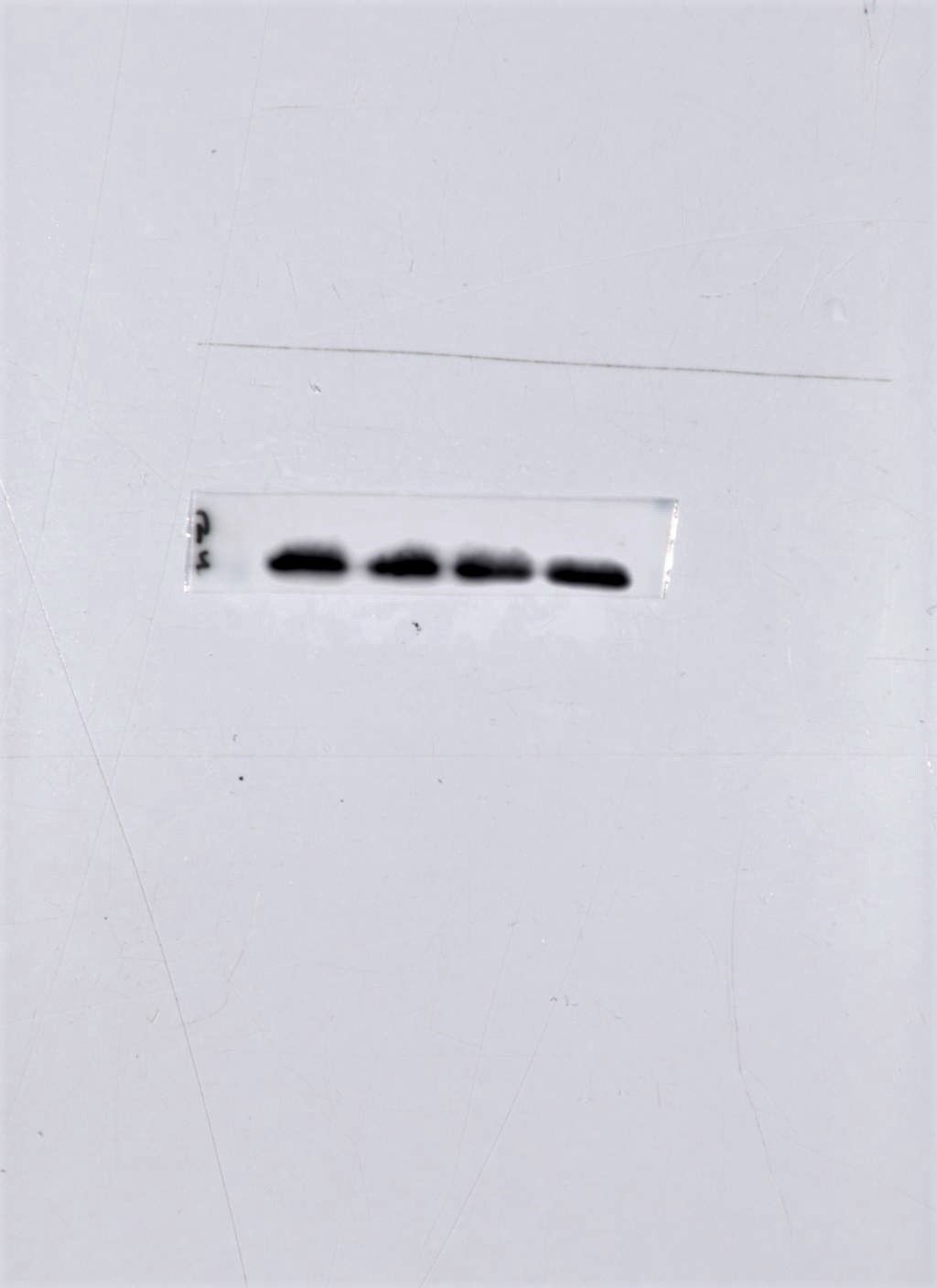

Supplement: Supplemental Information 1 [file peerj-08-8514-s001.zip › western blot/Figure1/Figure1B/BEAS-2B/GAPDH.jpg]

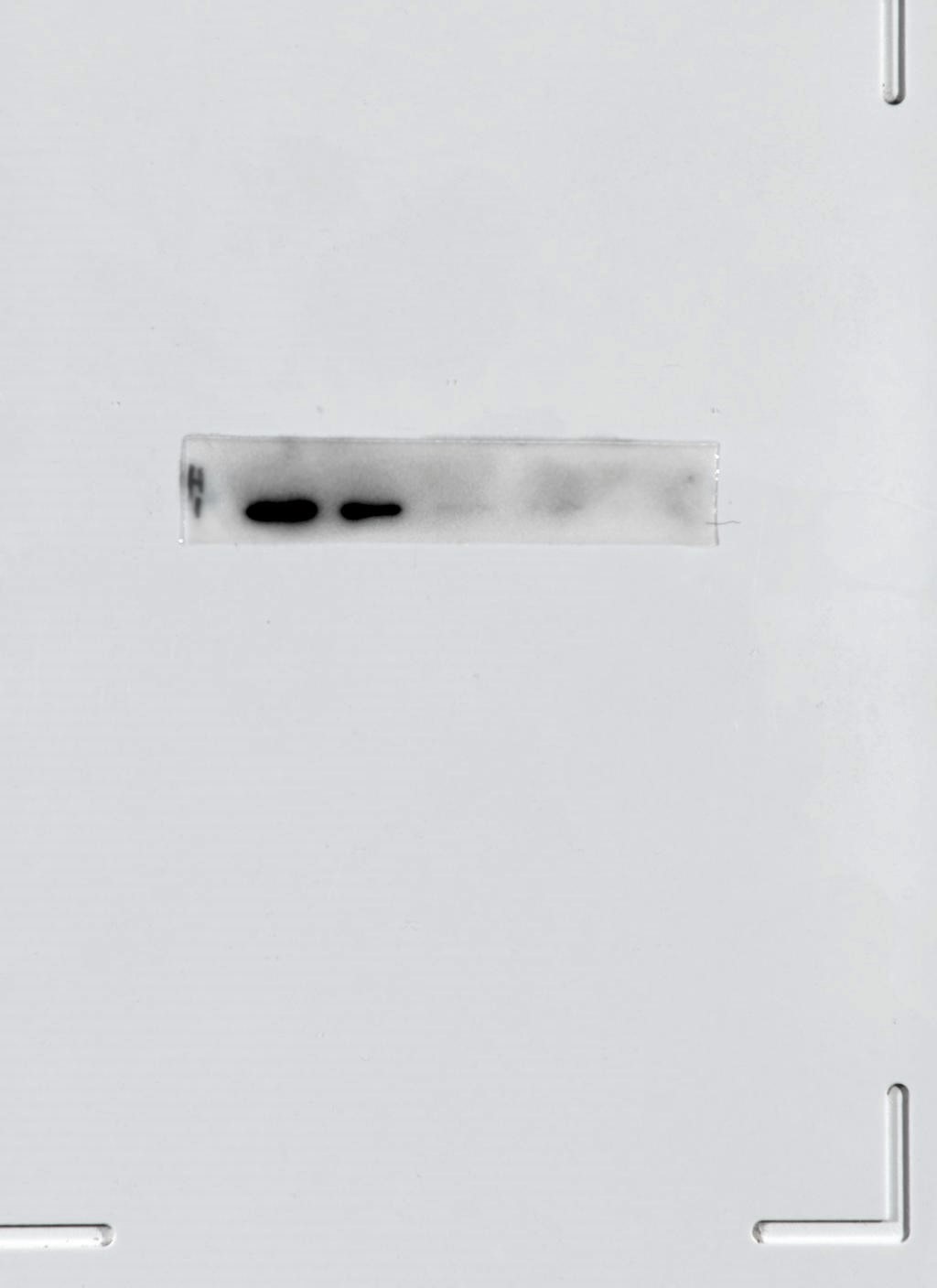

Supplement: Supplemental Information 1 [file peerj-08-8514-s001.zip › western blot/Figure1/Figure1B/BEAS-2B/HMGB1.jpg]

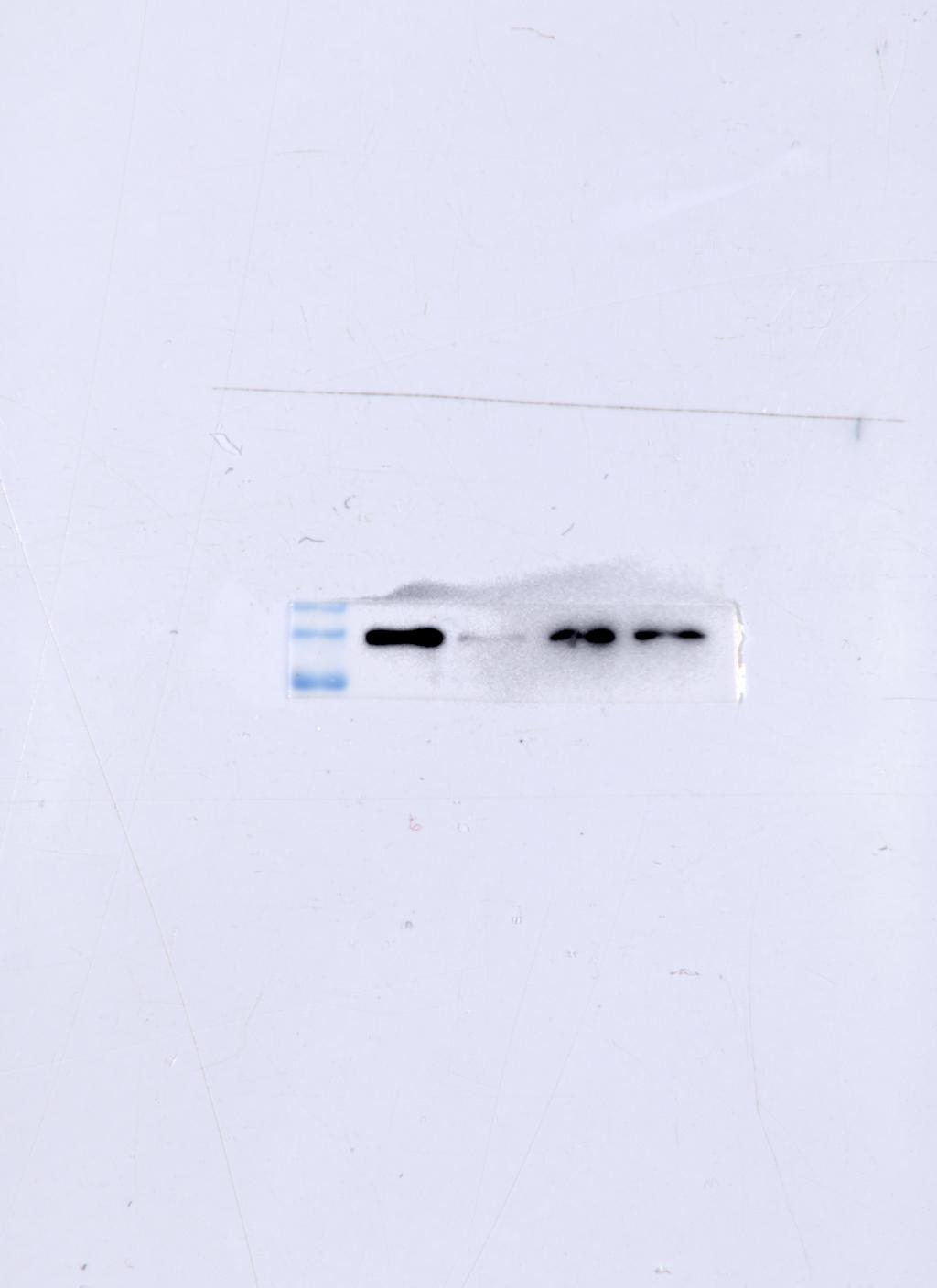

Supplement: Supplemental Information 1 [file peerj-08-8514-s001.zip › western blot/Figure1/Figure1C/A549/E-cadherin.jpg]

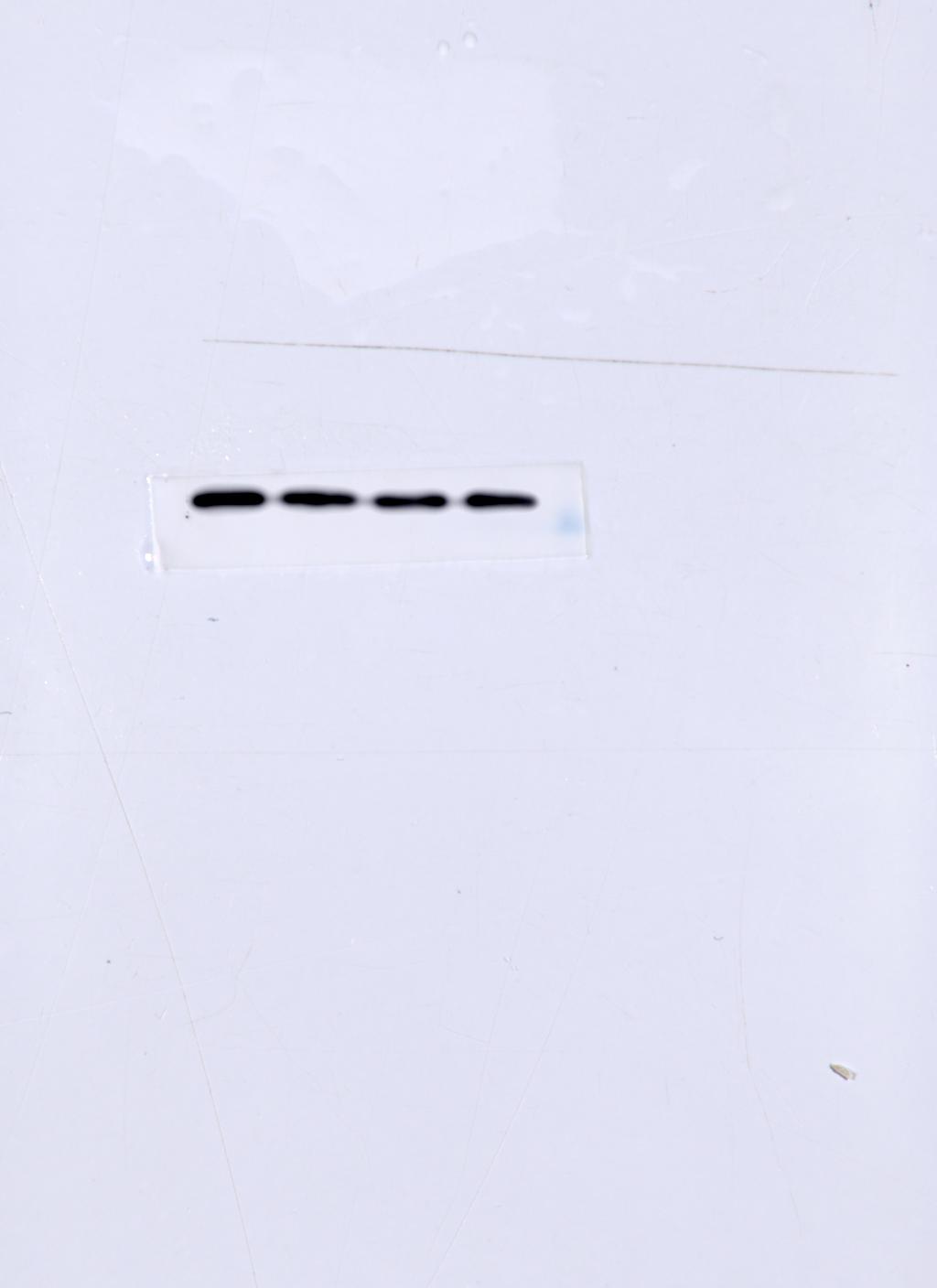

Supplement: Supplemental Information 1 [file peerj-08-8514-s001.zip › western blot/Figure1/Figure1C/A549/GAPDH.jpg]

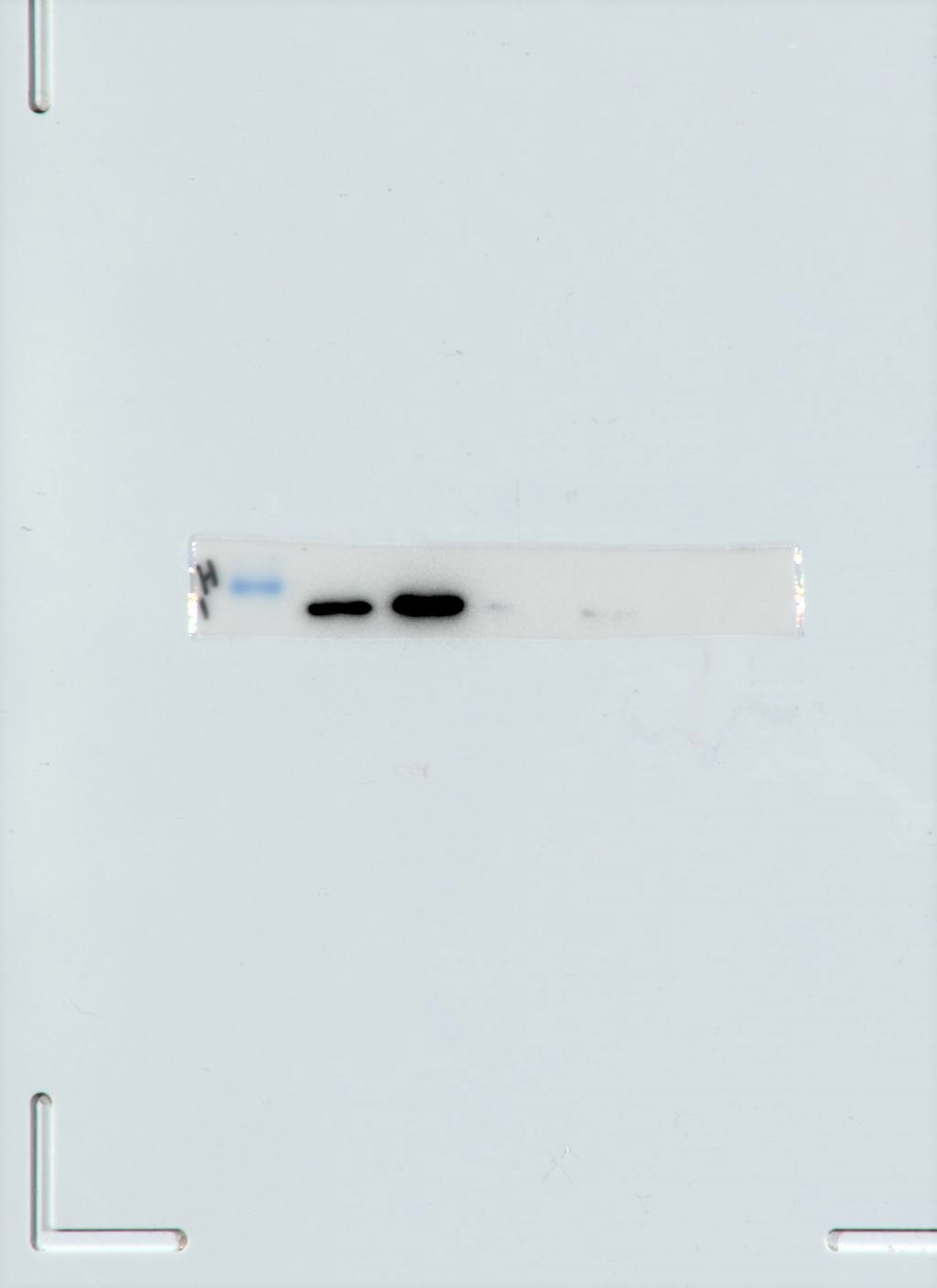

Supplement: Supplemental Information 1 [file peerj-08-8514-s001.zip › western blot/Figure1/Figure1C/A549/HMGB1.jpg]

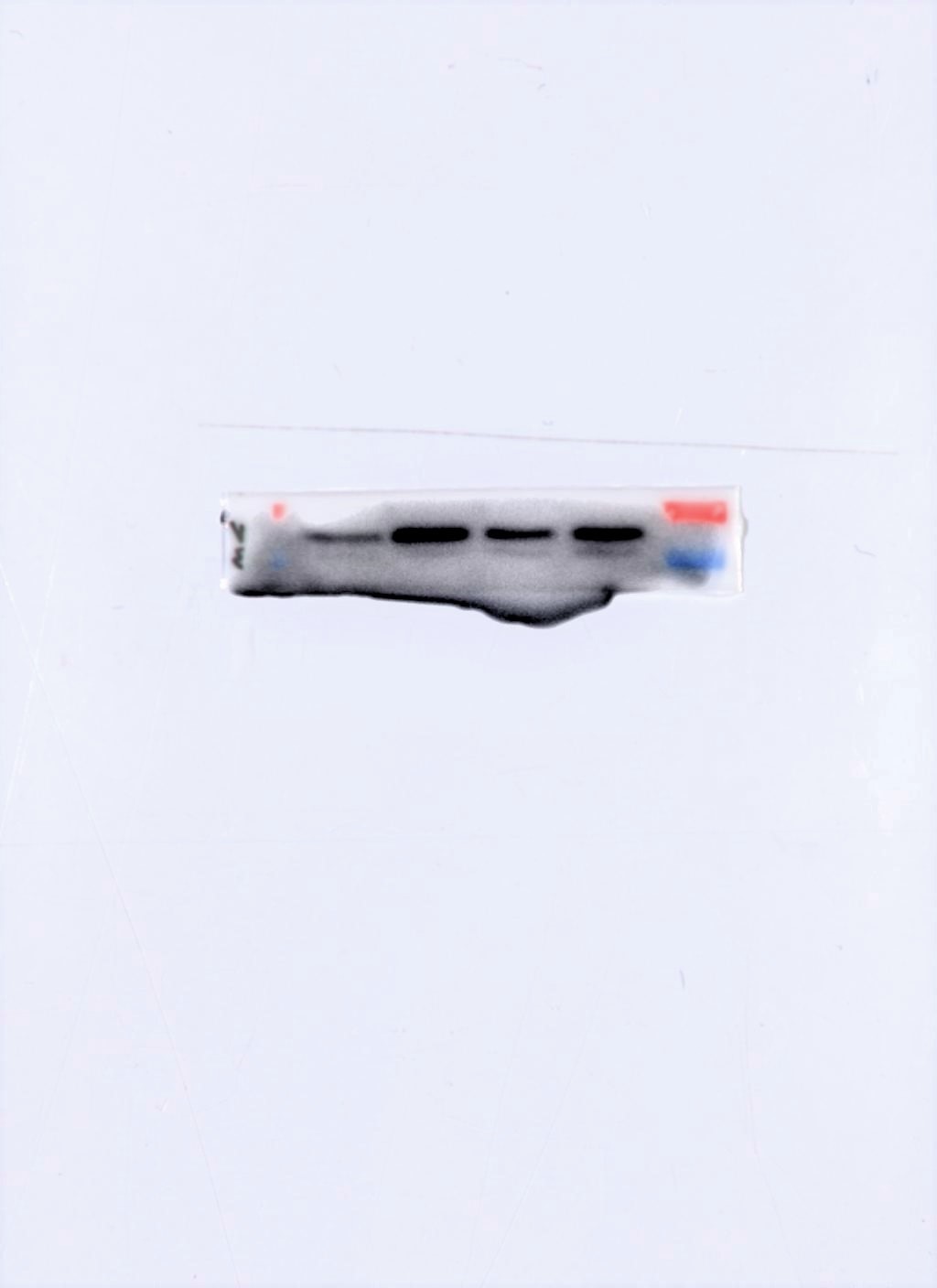

Supplement: Supplemental Information 1 [file peerj-08-8514-s001.zip › western blot/Figure1/Figure1C/A549/Vimentin.jpg]

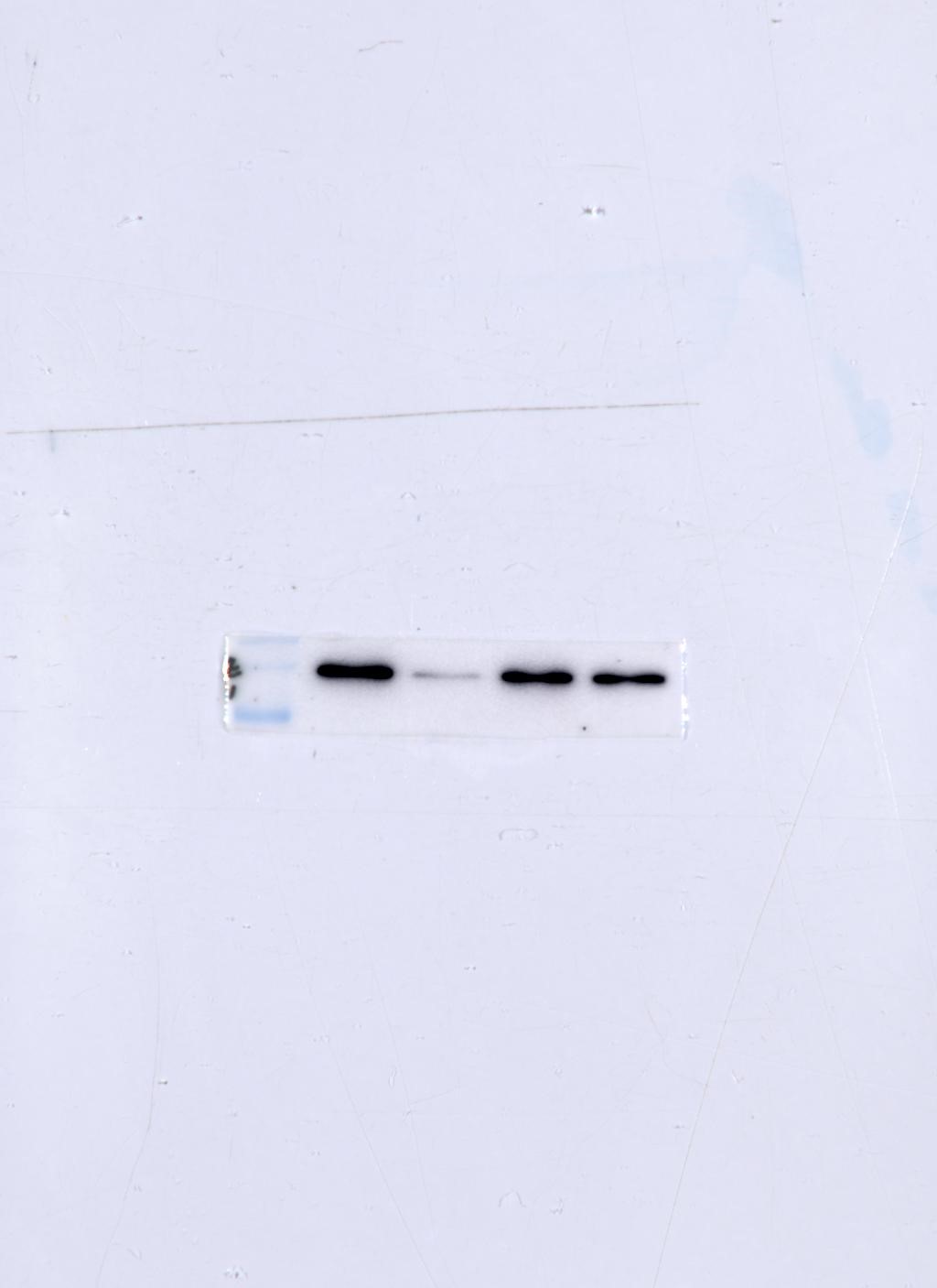

Supplement: Supplemental Information 1 [file peerj-08-8514-s001.zip › western blot/Figure1/Figure1C/BEAS-2B/E-cadherin.jpg]

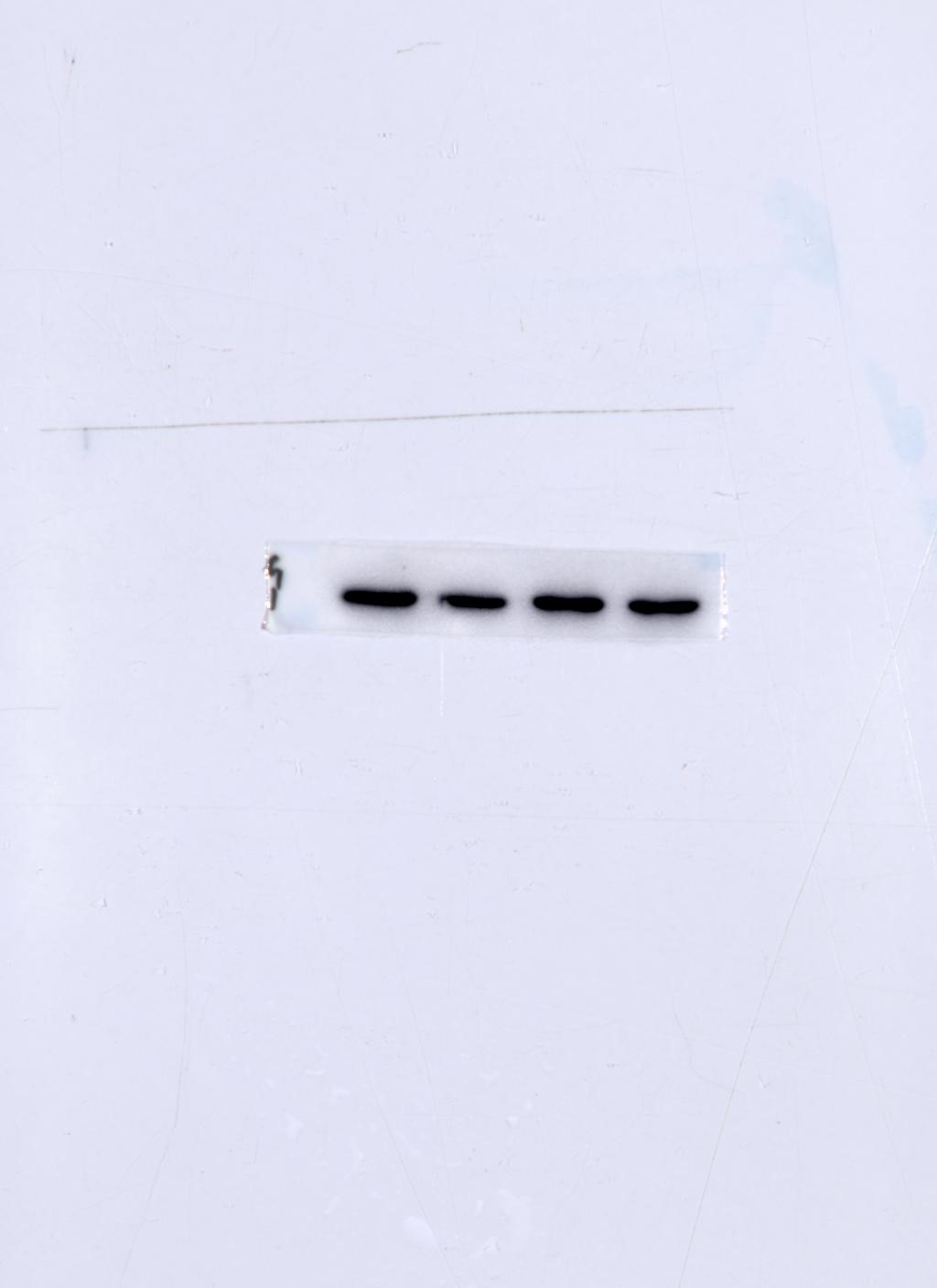

Supplement: Supplemental Information 1 [file peerj-08-8514-s001.zip › western blot/Figure1/Figure1C/BEAS-2B/GAPDH.jpg]

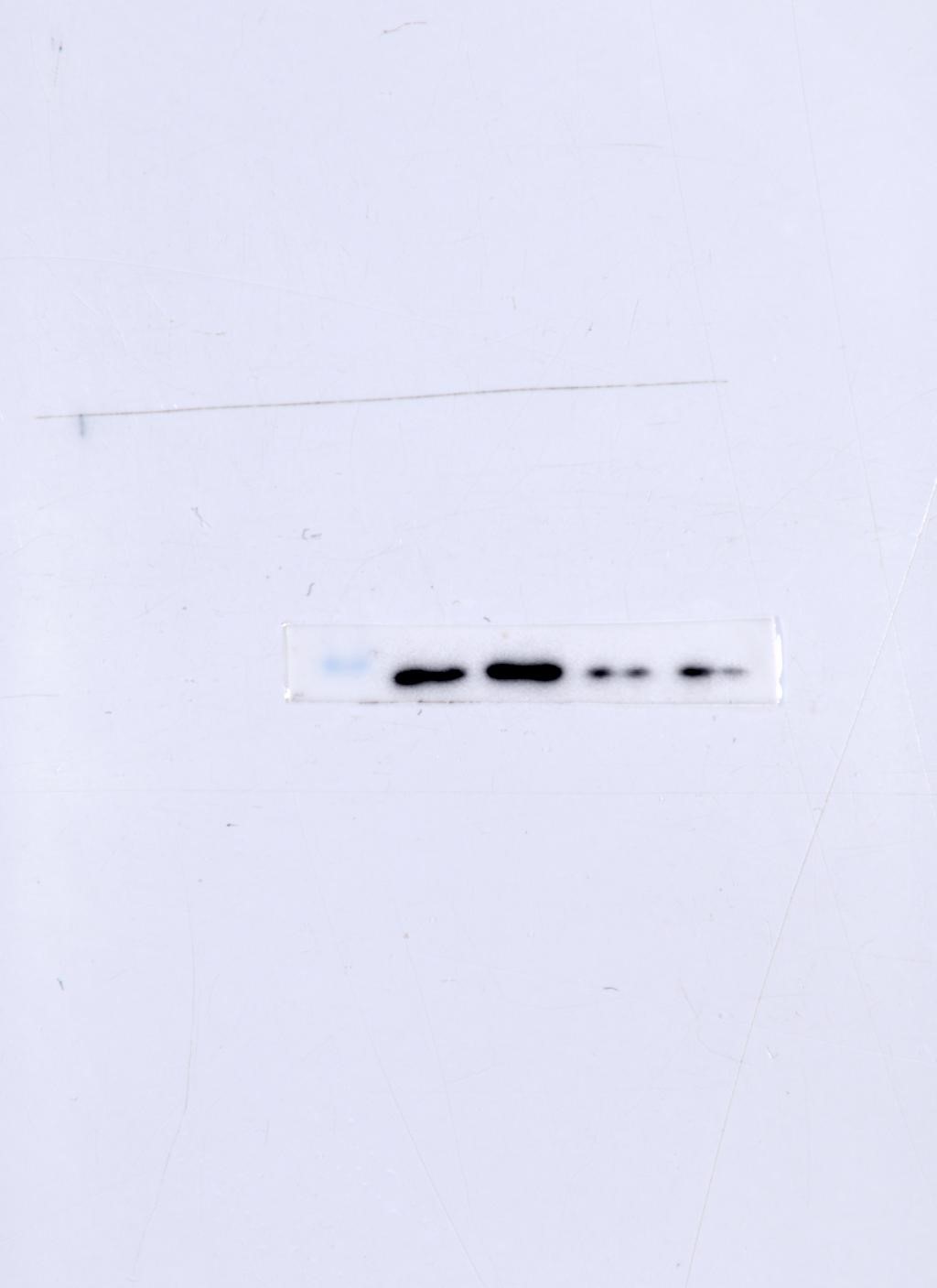

Supplement: Supplemental Information 1 [file peerj-08-8514-s001.zip › western blot/Figure1/Figure1C/BEAS-2B/HMGB1.jpg]

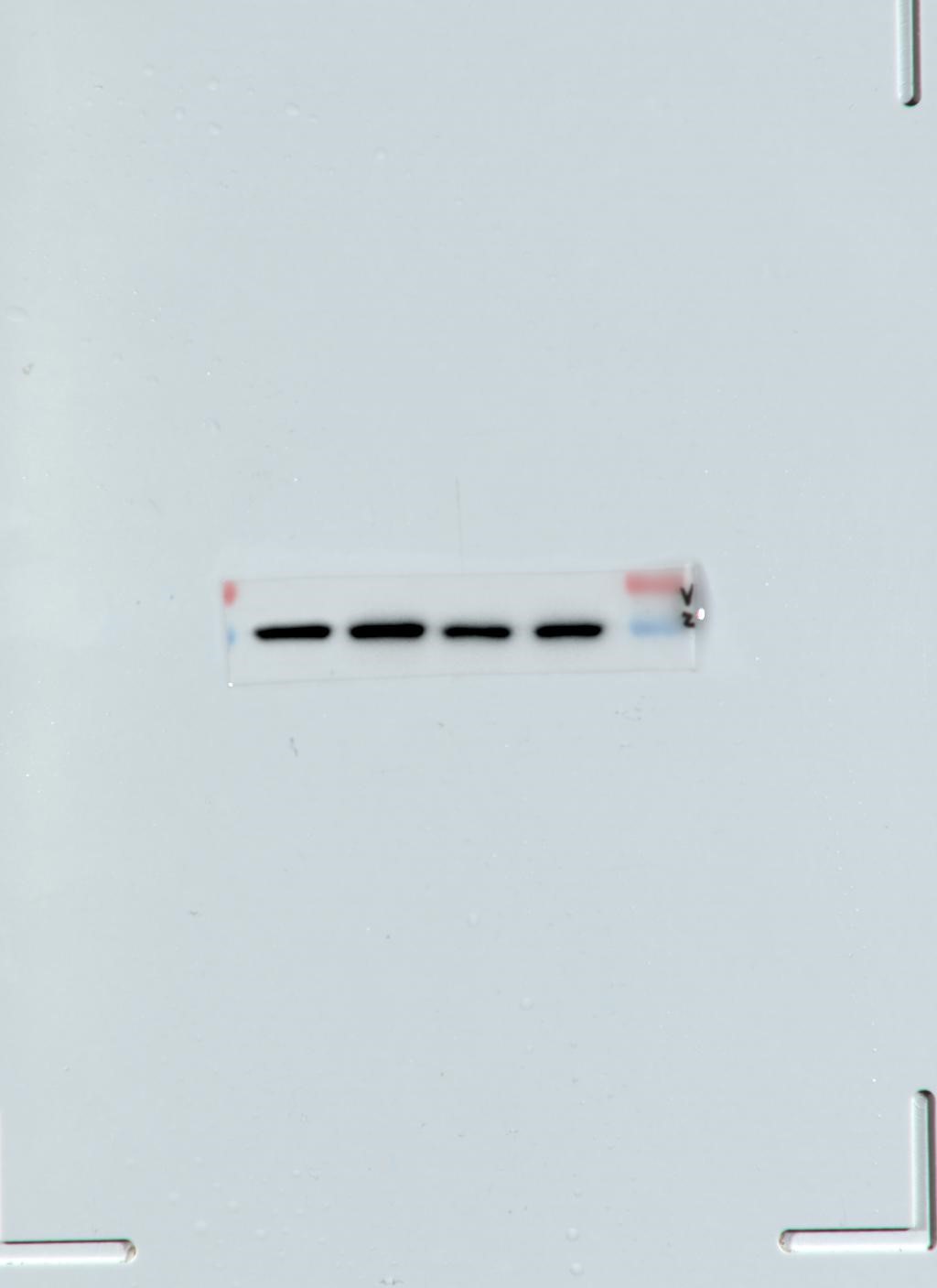

Supplement: Supplemental Information 1 [file peerj-08-8514-s001.zip › western blot/Figure1/Figure1C/BEAS-2B/Vimentin.jpg]

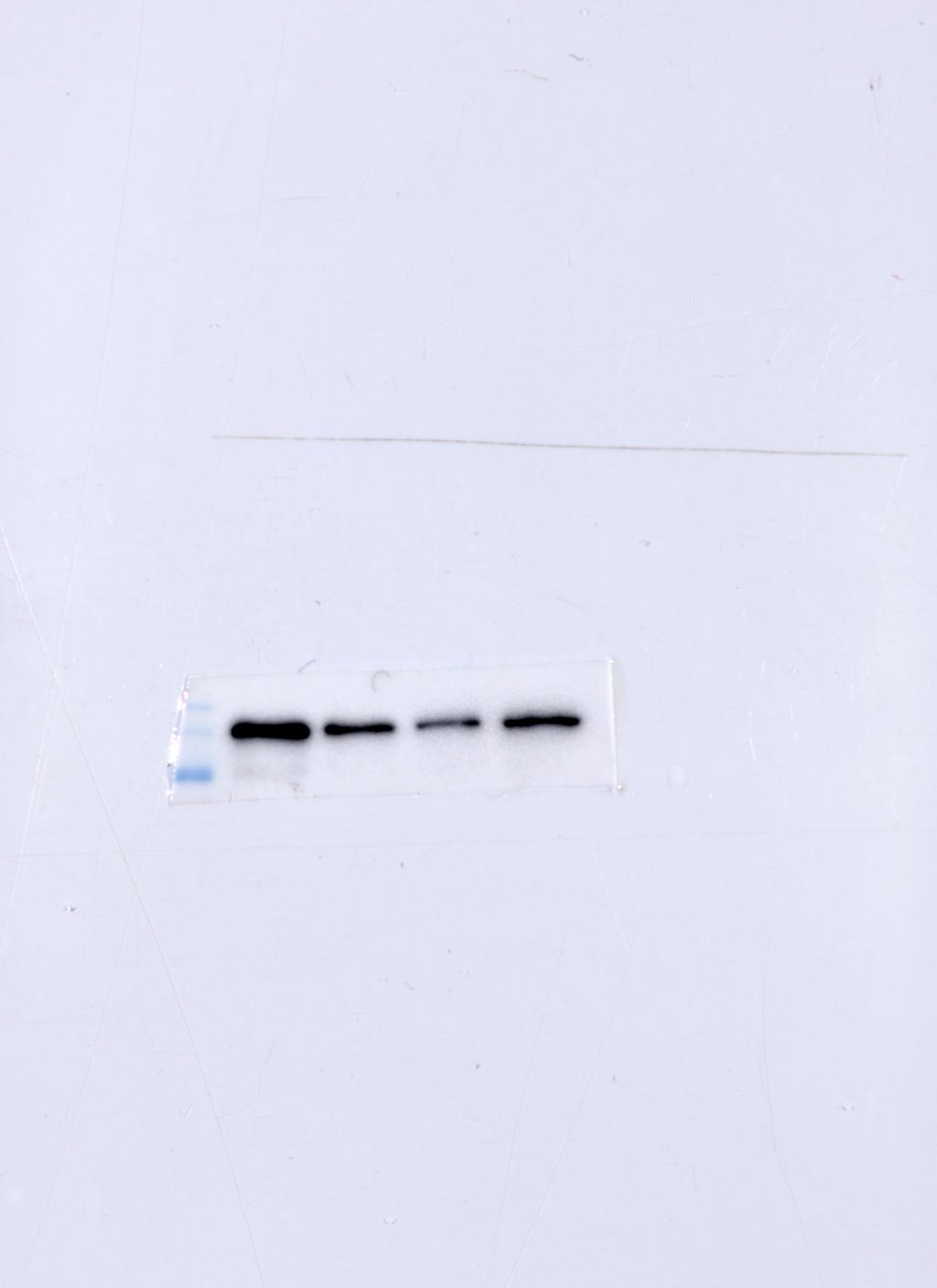

Supplement: Supplemental Information 1 [file peerj-08-8514-s001.zip › western blot/Figure3/Figure3B/A549/E-cadherin.jpg]

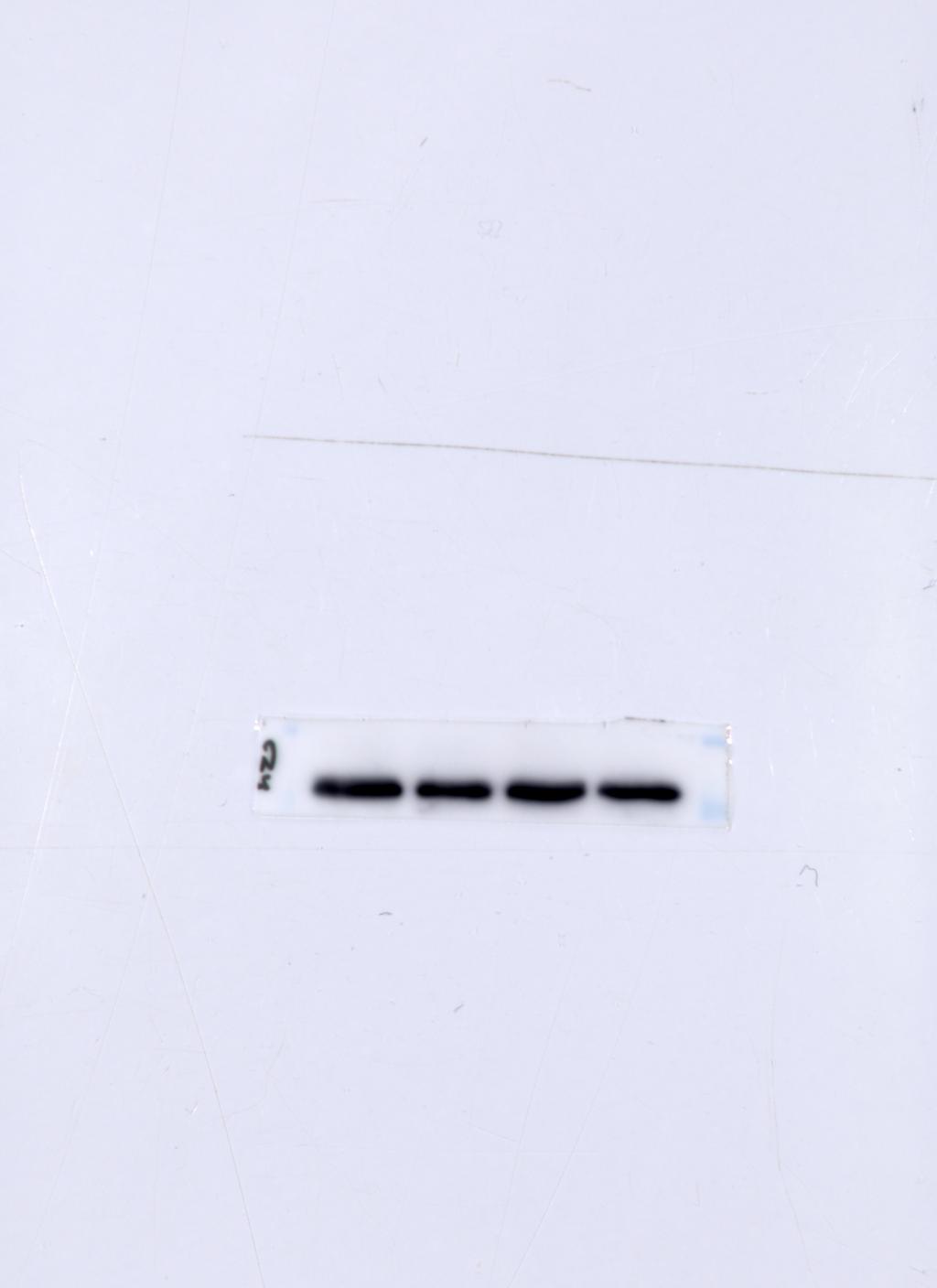

Supplement: Supplemental Information 1 [file peerj-08-8514-s001.zip › western blot/Figure3/Figure3B/A549/GAPDH.jpg]

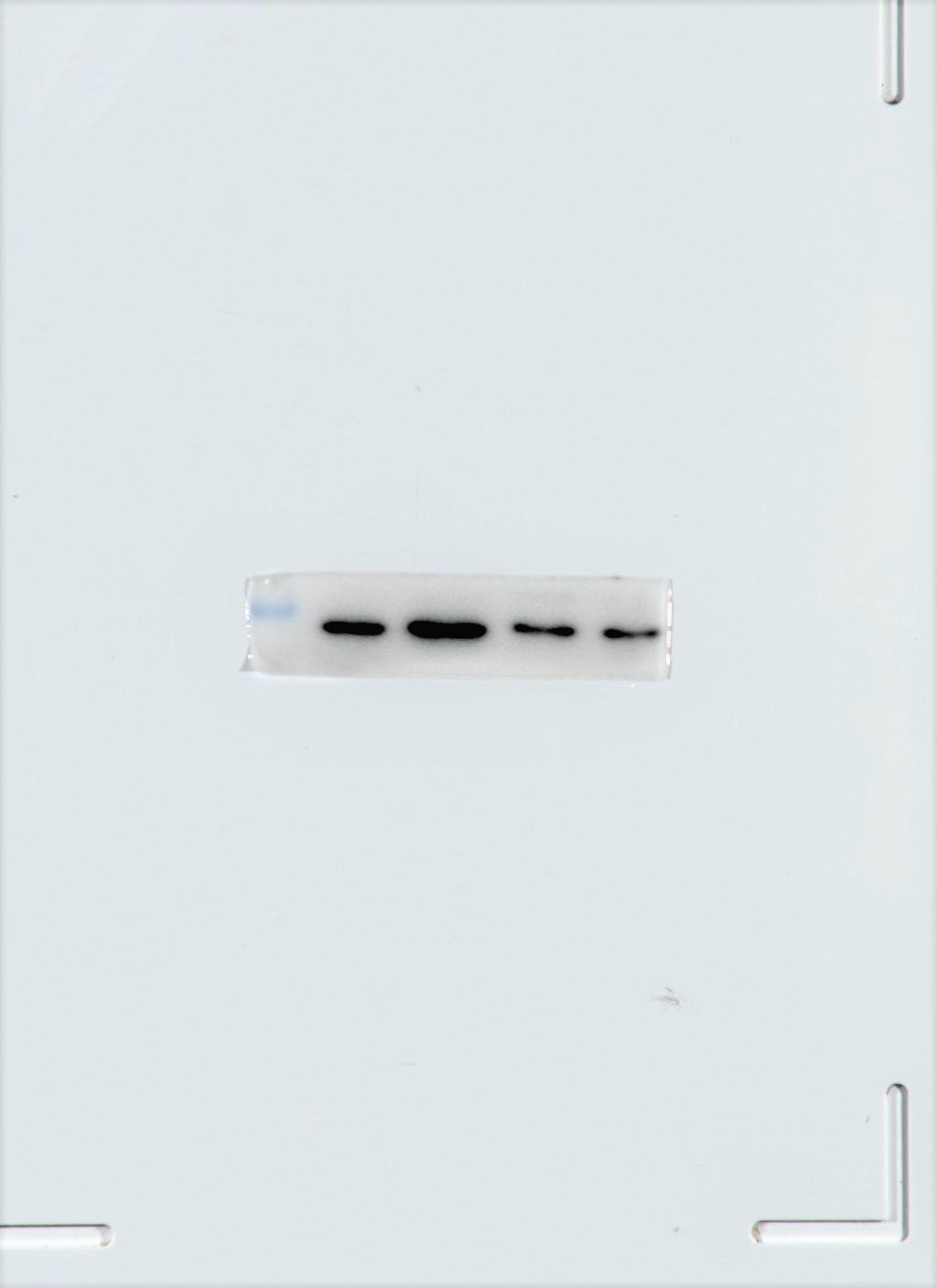

Supplement: Supplemental Information 1 [file peerj-08-8514-s001.zip › western blot/Figure3/Figure3B/A549/HMGB1.jpg]

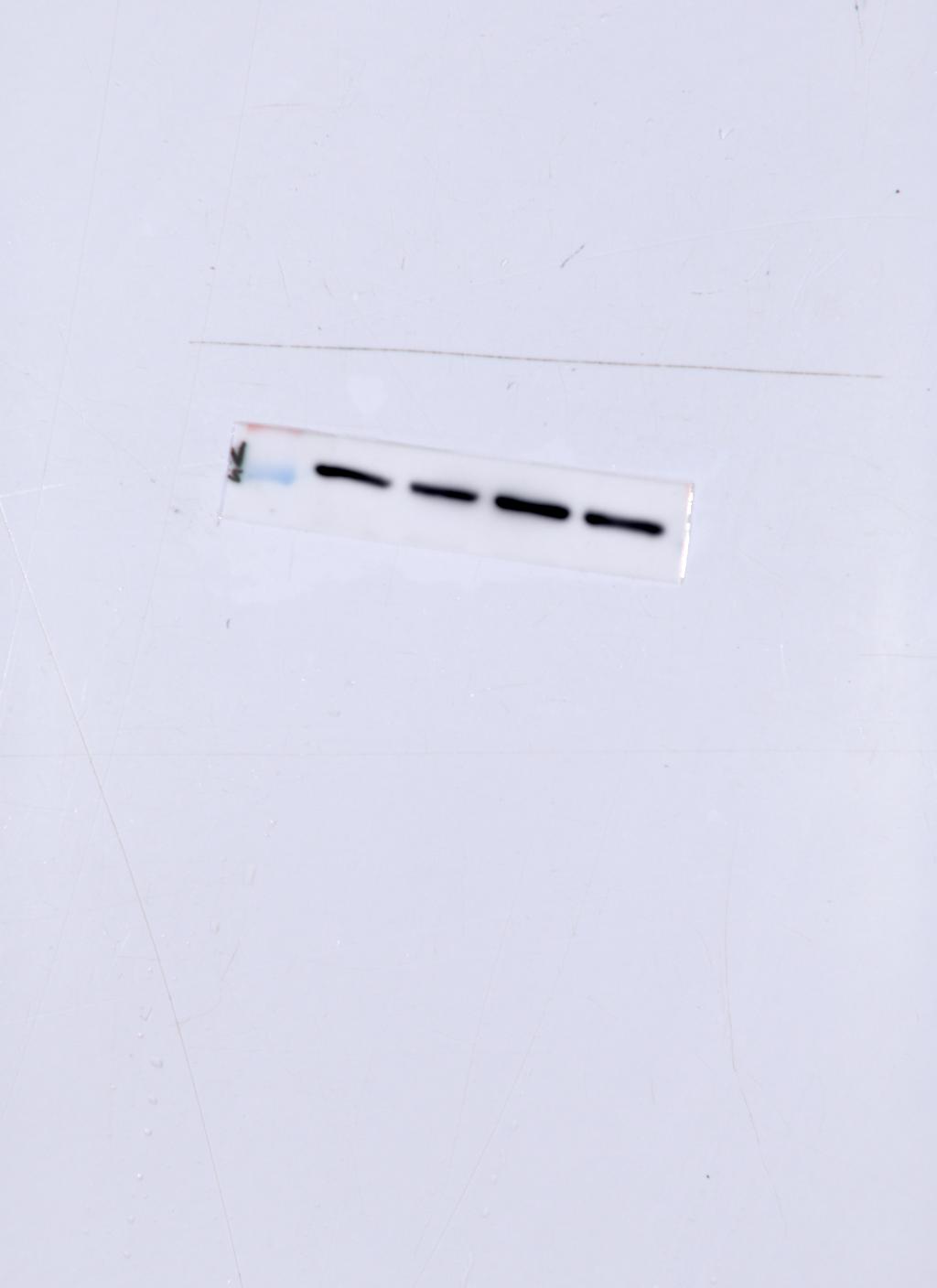

Supplement: Supplemental Information 1 [file peerj-08-8514-s001.zip › western blot/Figure3/Figure3B/A549/Vimentin.jpg]

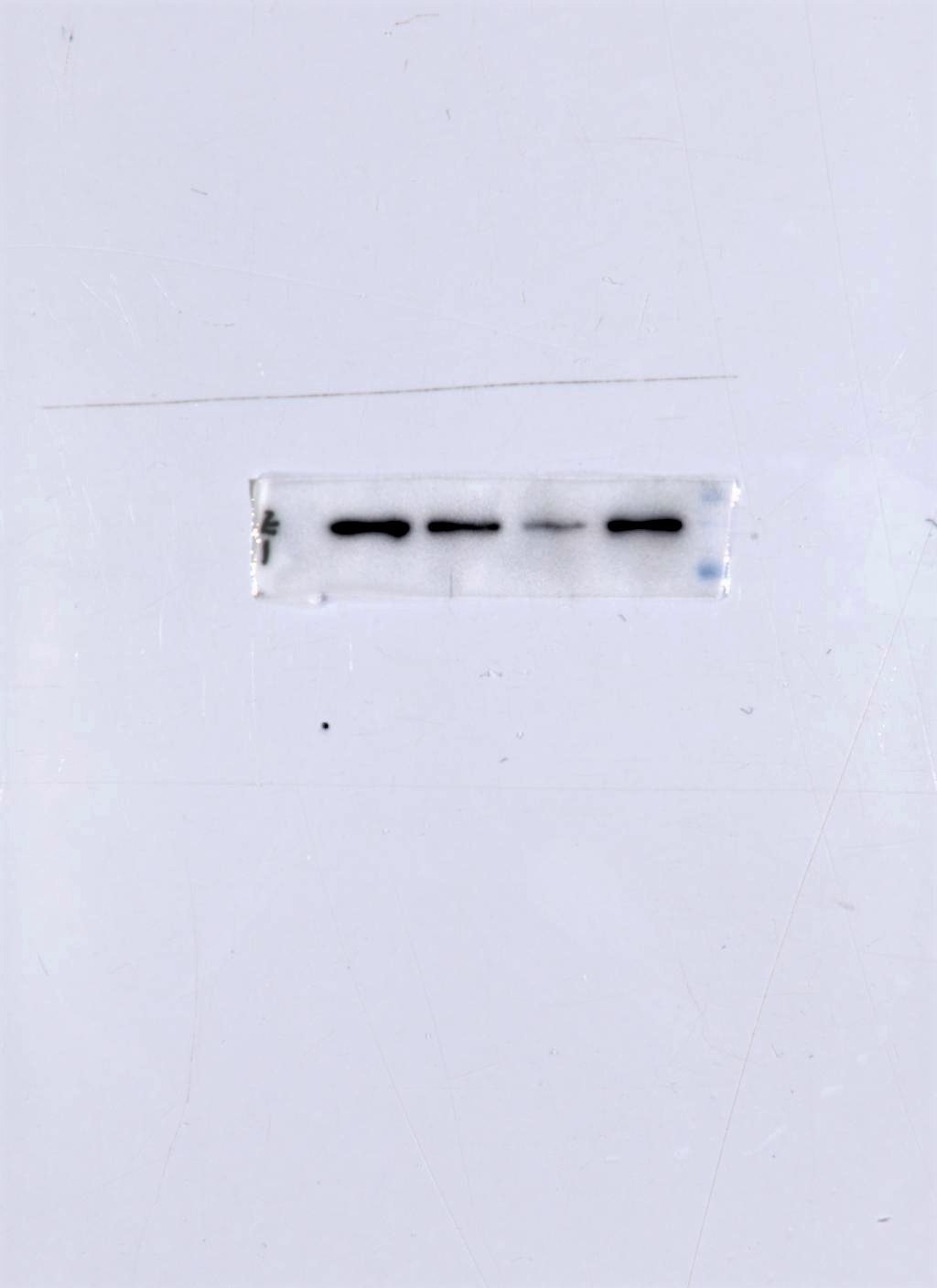

Supplement: Supplemental Information 1 [file peerj-08-8514-s001.zip › western blot/Figure3/Figure3B/BEAS-2B/E-cadherin.jpg]

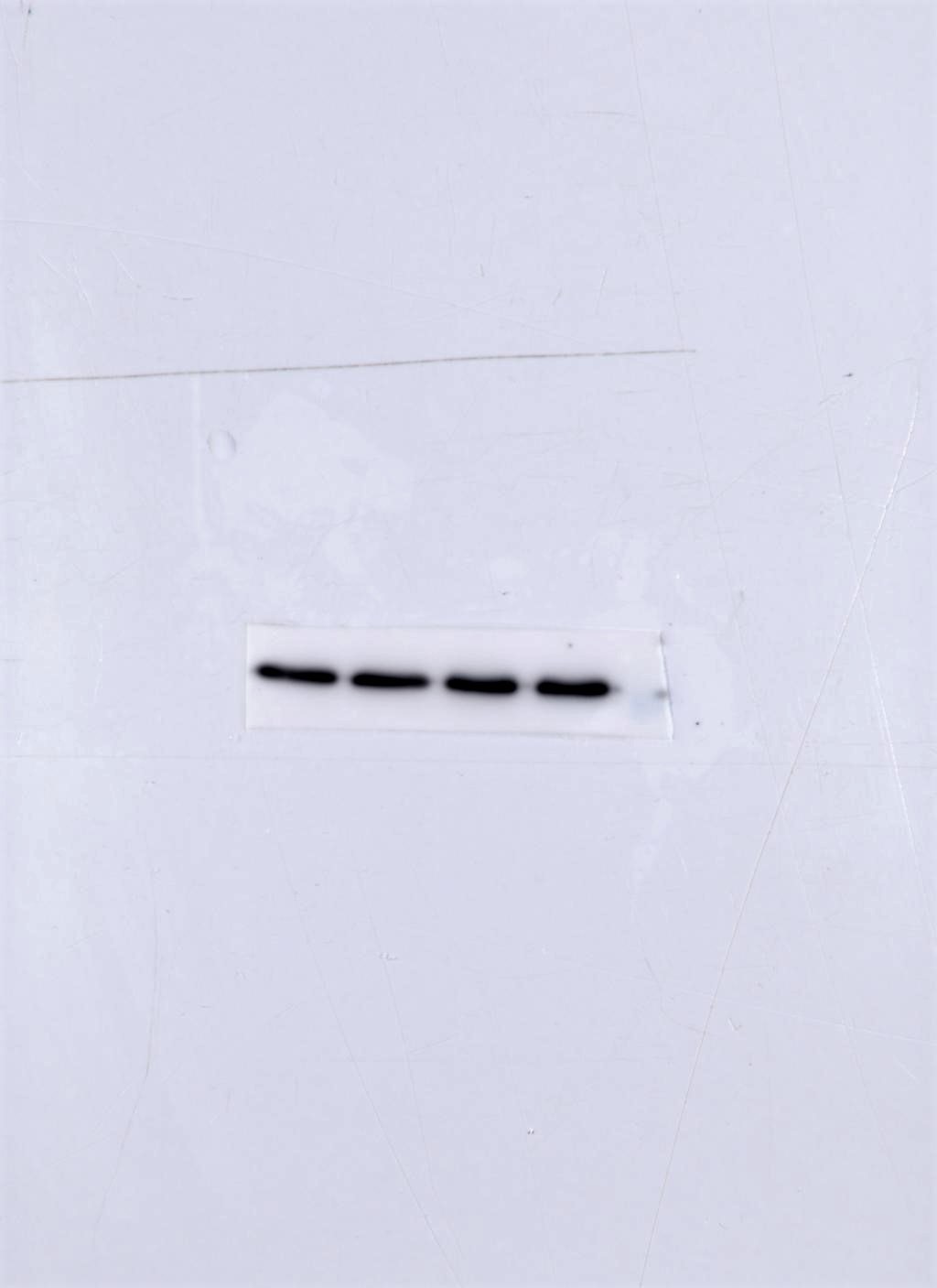

Supplement: Supplemental Information 1 [file peerj-08-8514-s001.zip › western blot/Figure3/Figure3B/BEAS-2B/GAPDH.jpg]

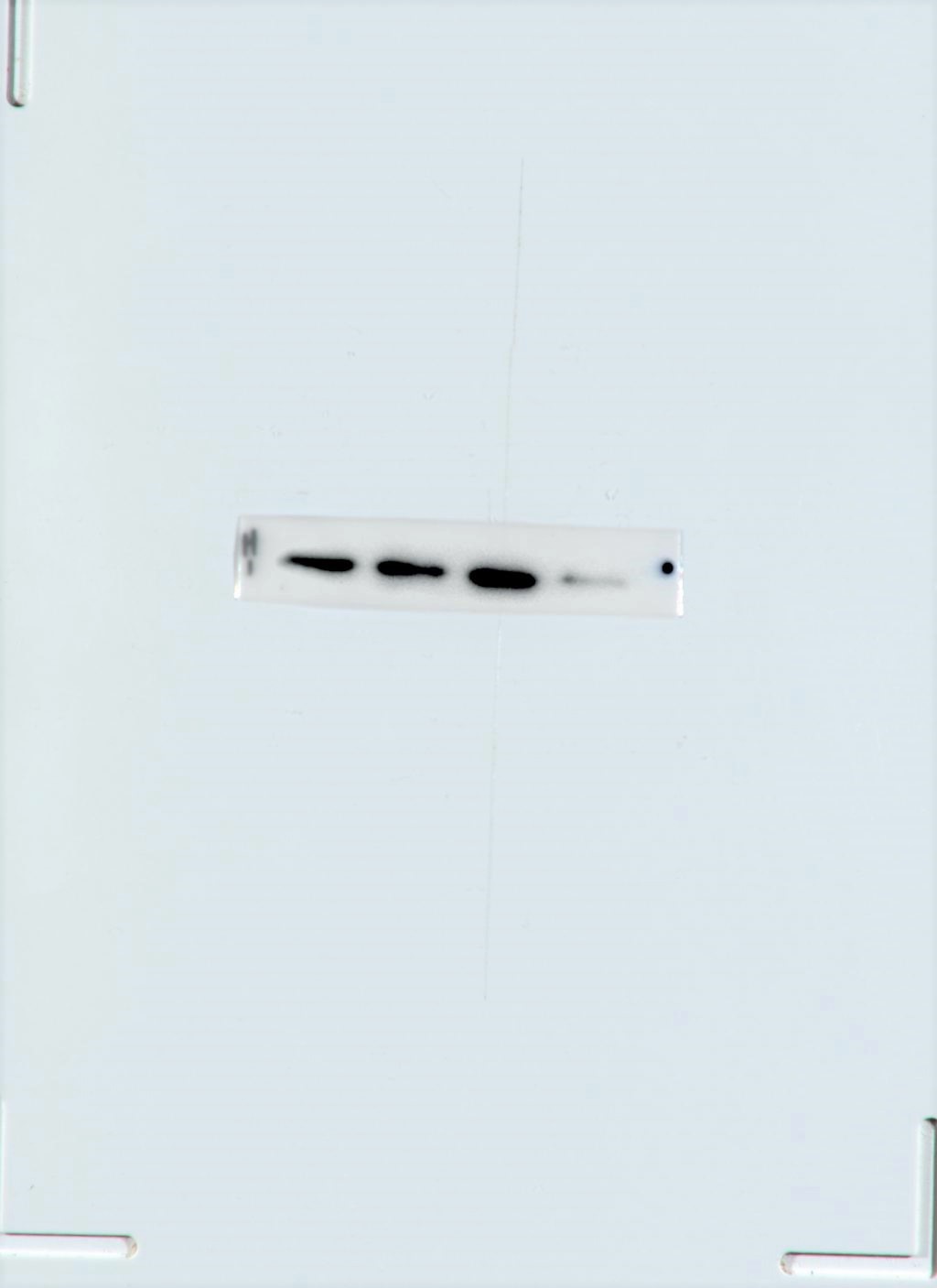

Supplement: Supplemental Information 1 [file peerj-08-8514-s001.zip › western blot/Figure3/Figure3B/BEAS-2B/HMGB1.jpg]

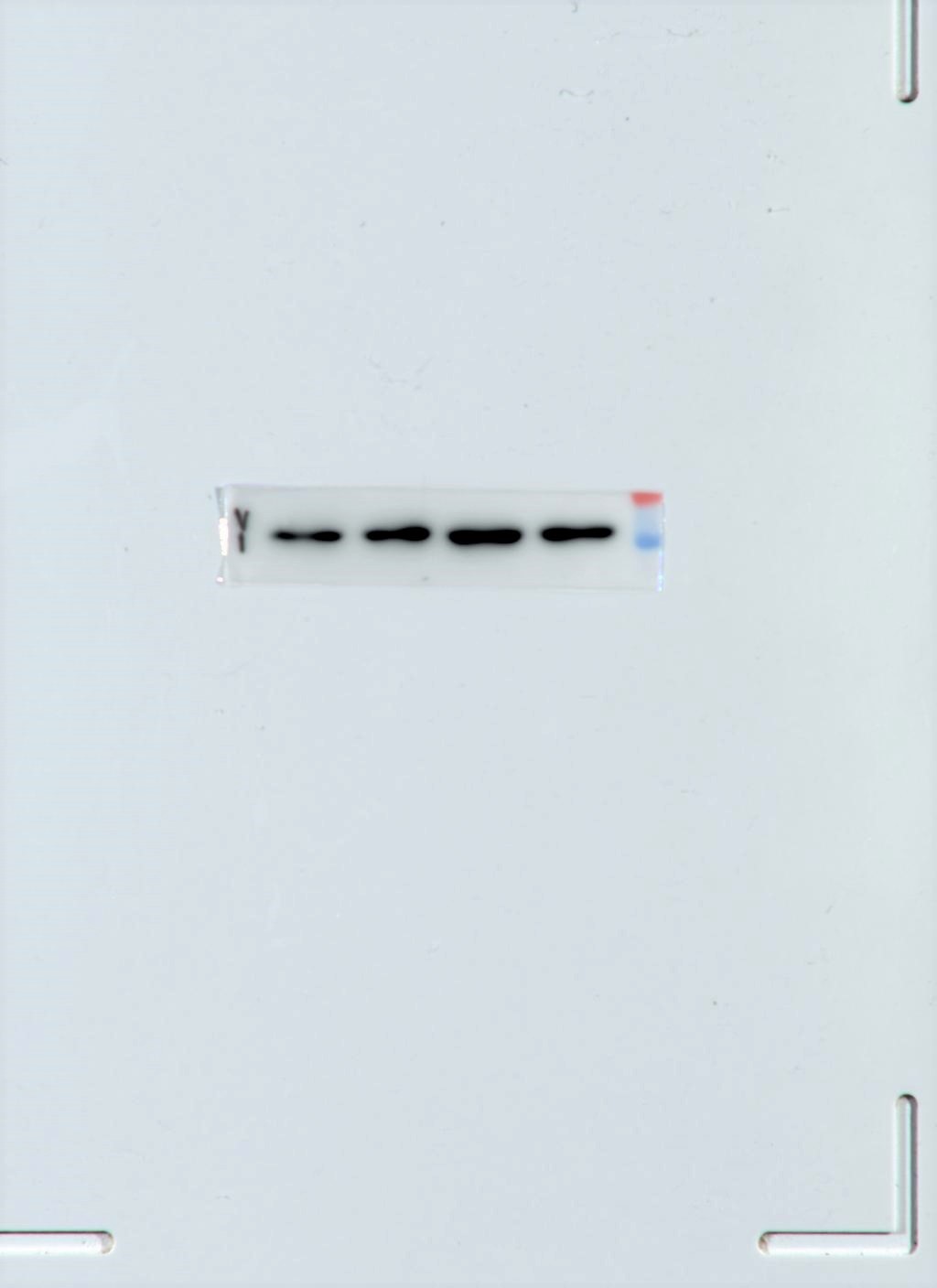

Supplement: Supplemental Information 1 [file peerj-08-8514-s001.zip › western blot/Figure3/Figure3B/BEAS-2B/Vimentin.jpg]

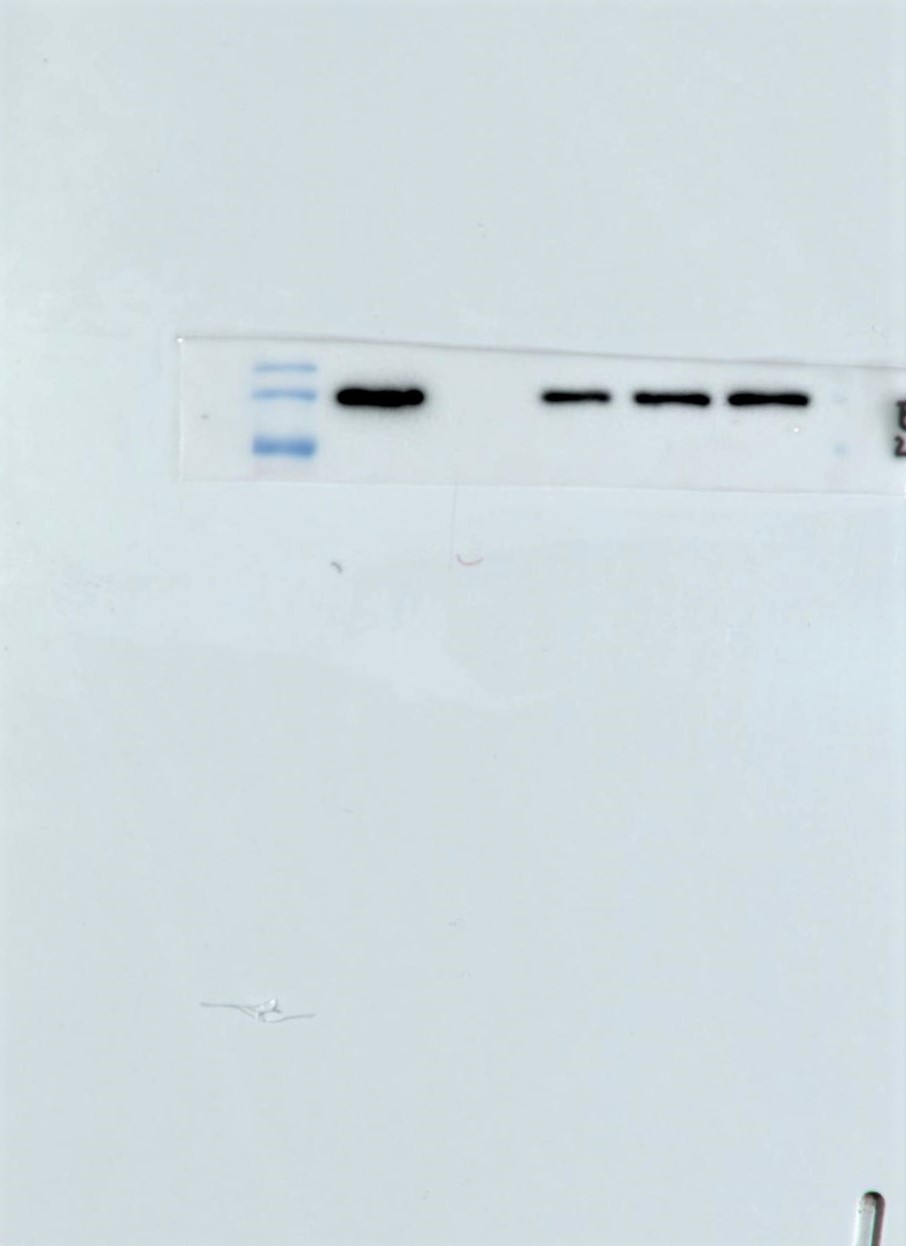

Supplement: Supplemental Information 1 [file peerj-08-8514-s001.zip › western blot/Figure3/Figure3C/A549/E-cadherin.jpg]

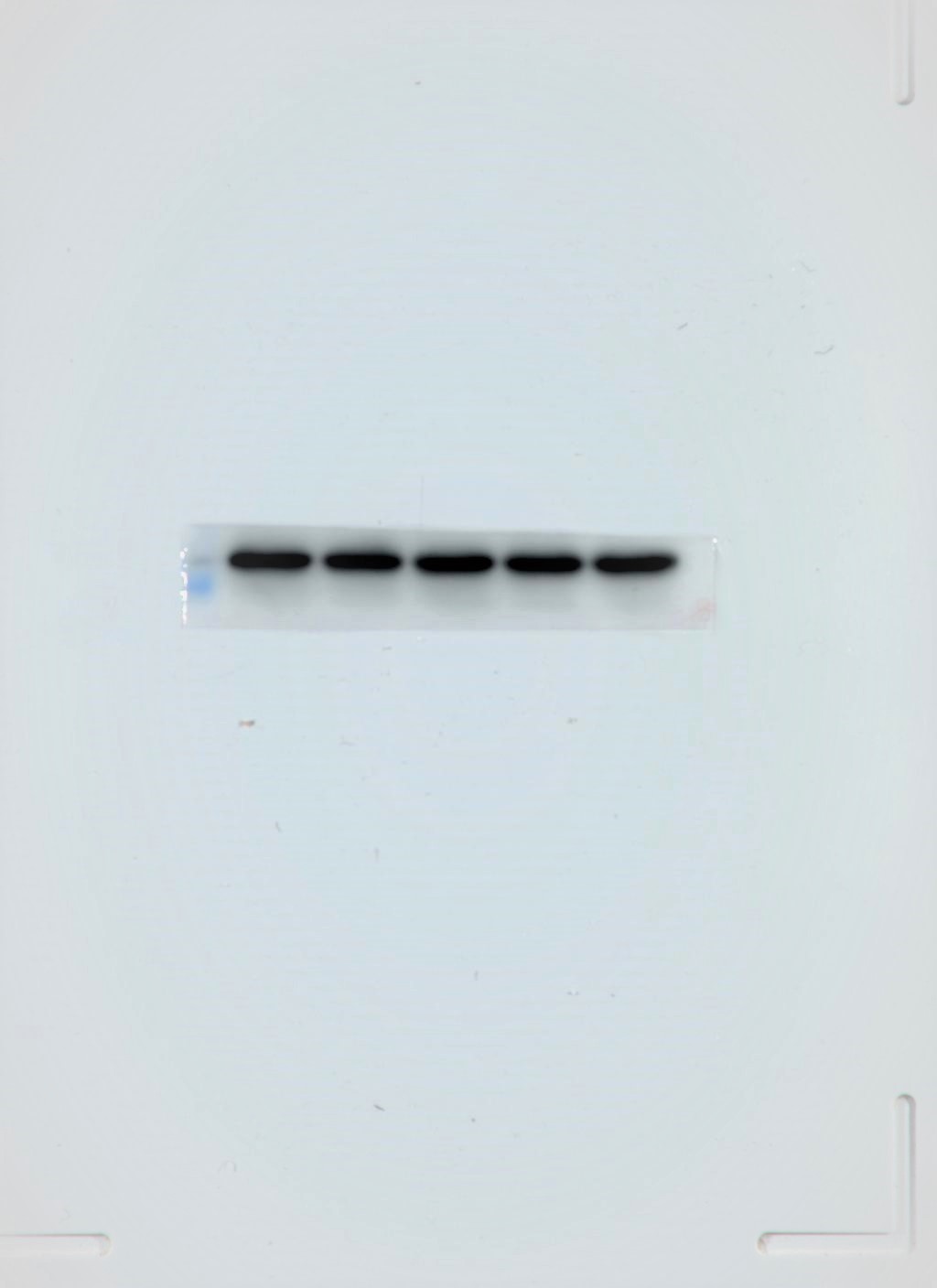

Supplement: Supplemental Information 1 [file peerj-08-8514-s001.zip › western blot/Figure3/Figure3C/A549/GAPDH.jpg]

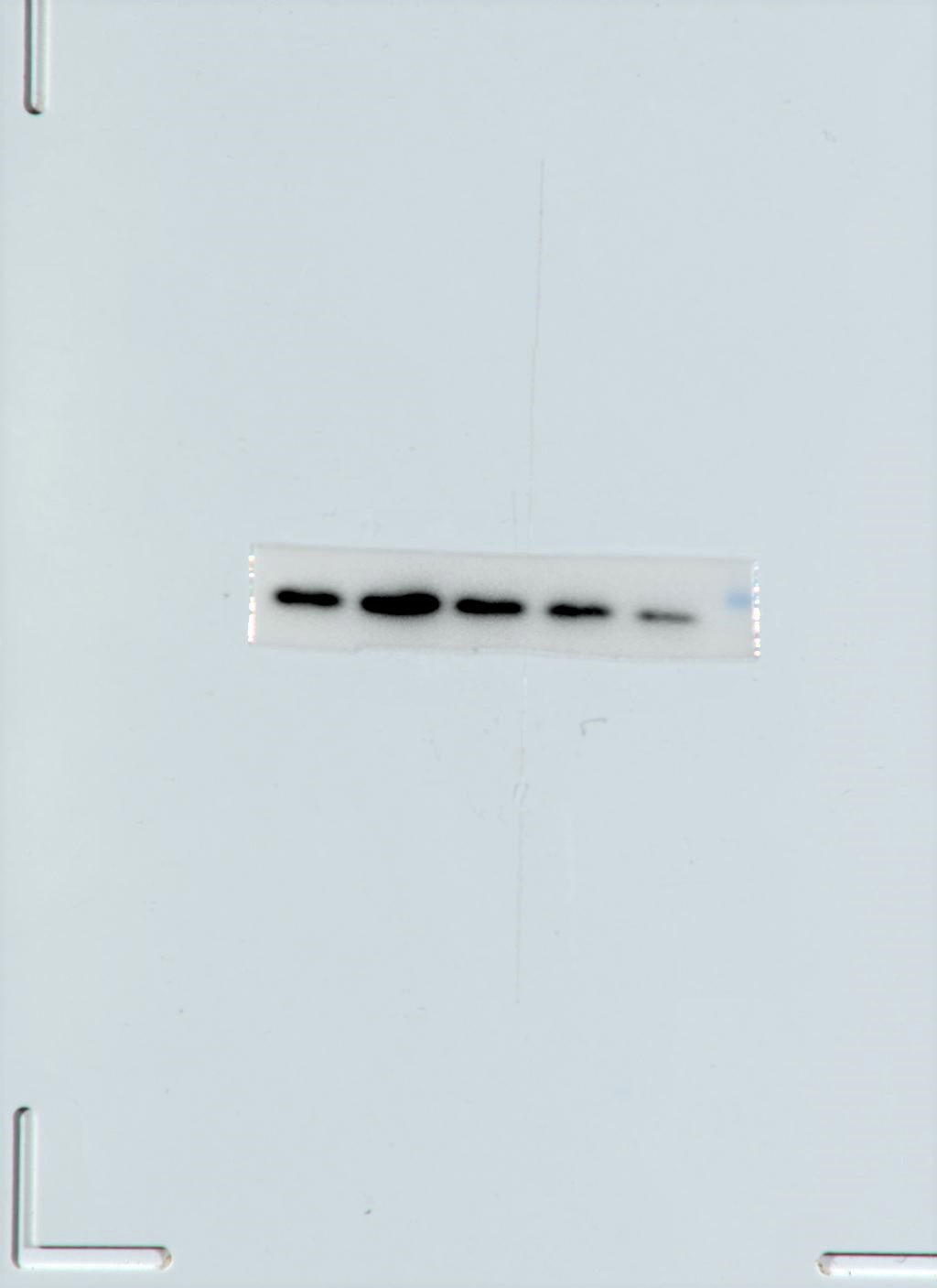

Supplement: Supplemental Information 1 [file peerj-08-8514-s001.zip › western blot/Figure3/Figure3C/A549/HMGB1.jpg]

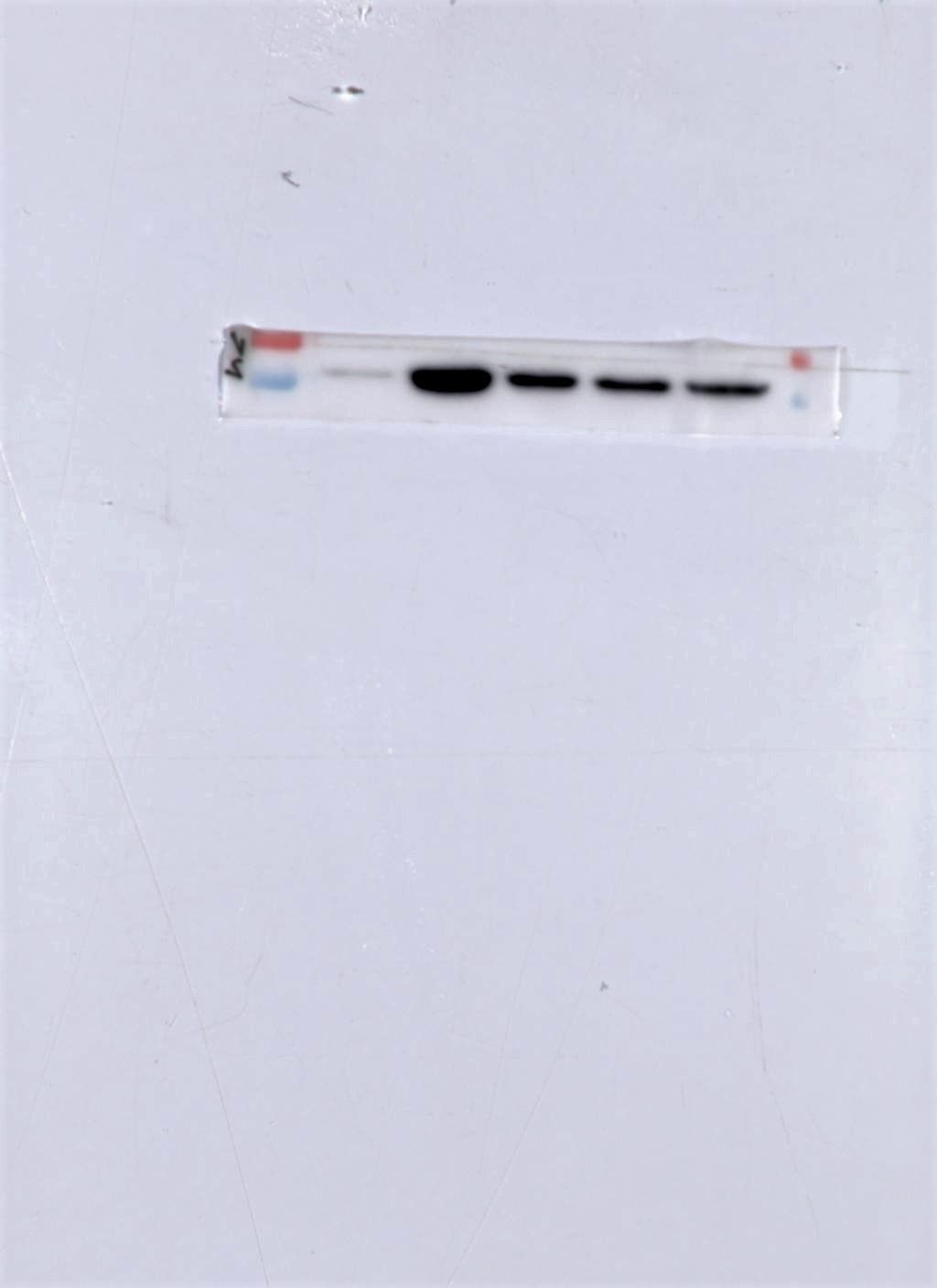

Supplement: Supplemental Information 1 [file peerj-08-8514-s001.zip › western blot/Figure3/Figure3C/A549/Vimentin.jpg]

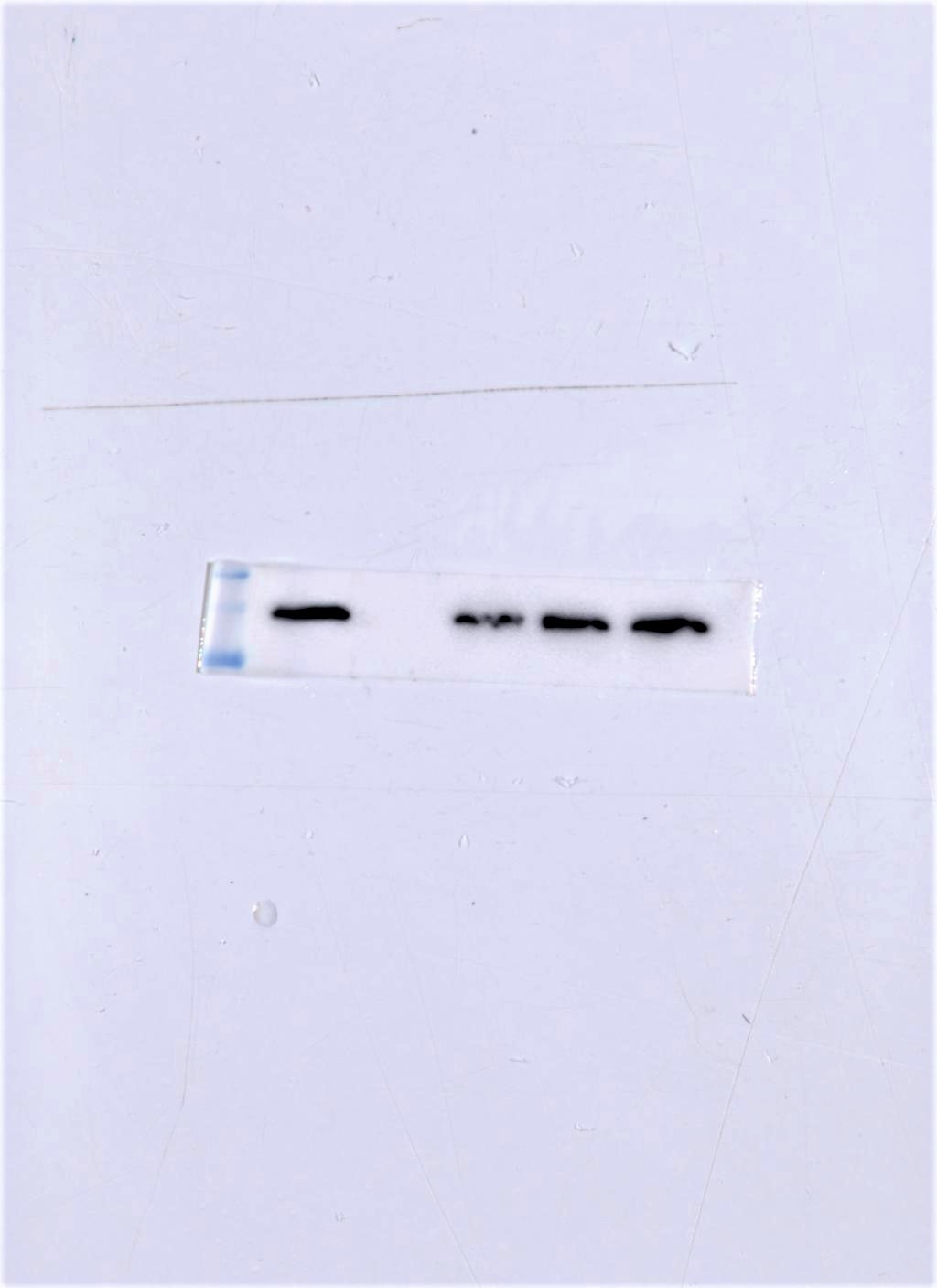

Supplement: Supplemental Information 1 [file peerj-08-8514-s001.zip › western blot/Figure3/Figure3C/BEAS-2B/E-cadherin.jpg]

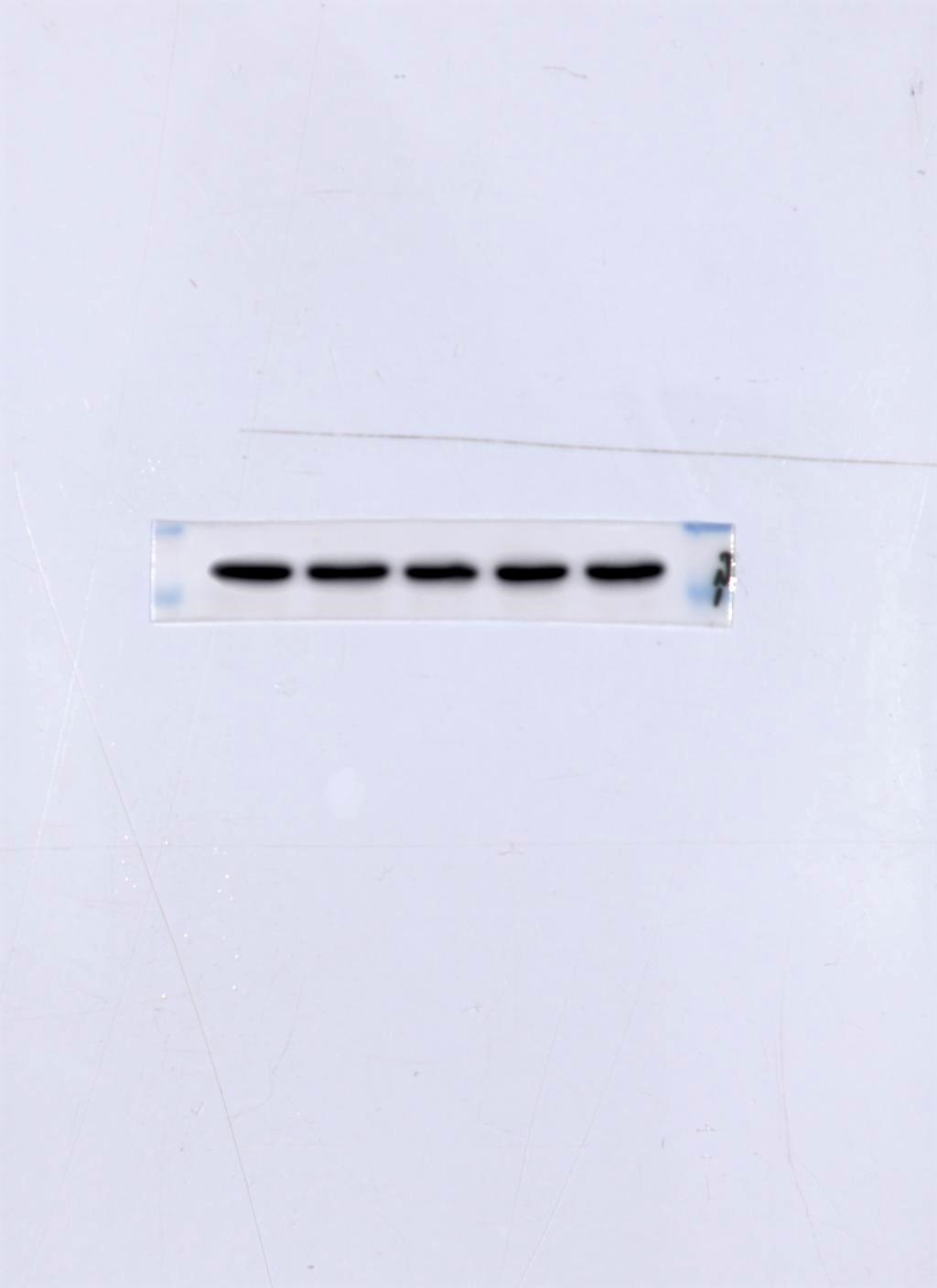

Supplement: Supplemental Information 1 [file peerj-08-8514-s001.zip › western blot/Figure3/Figure3C/BEAS-2B/GAPDH.jpg]

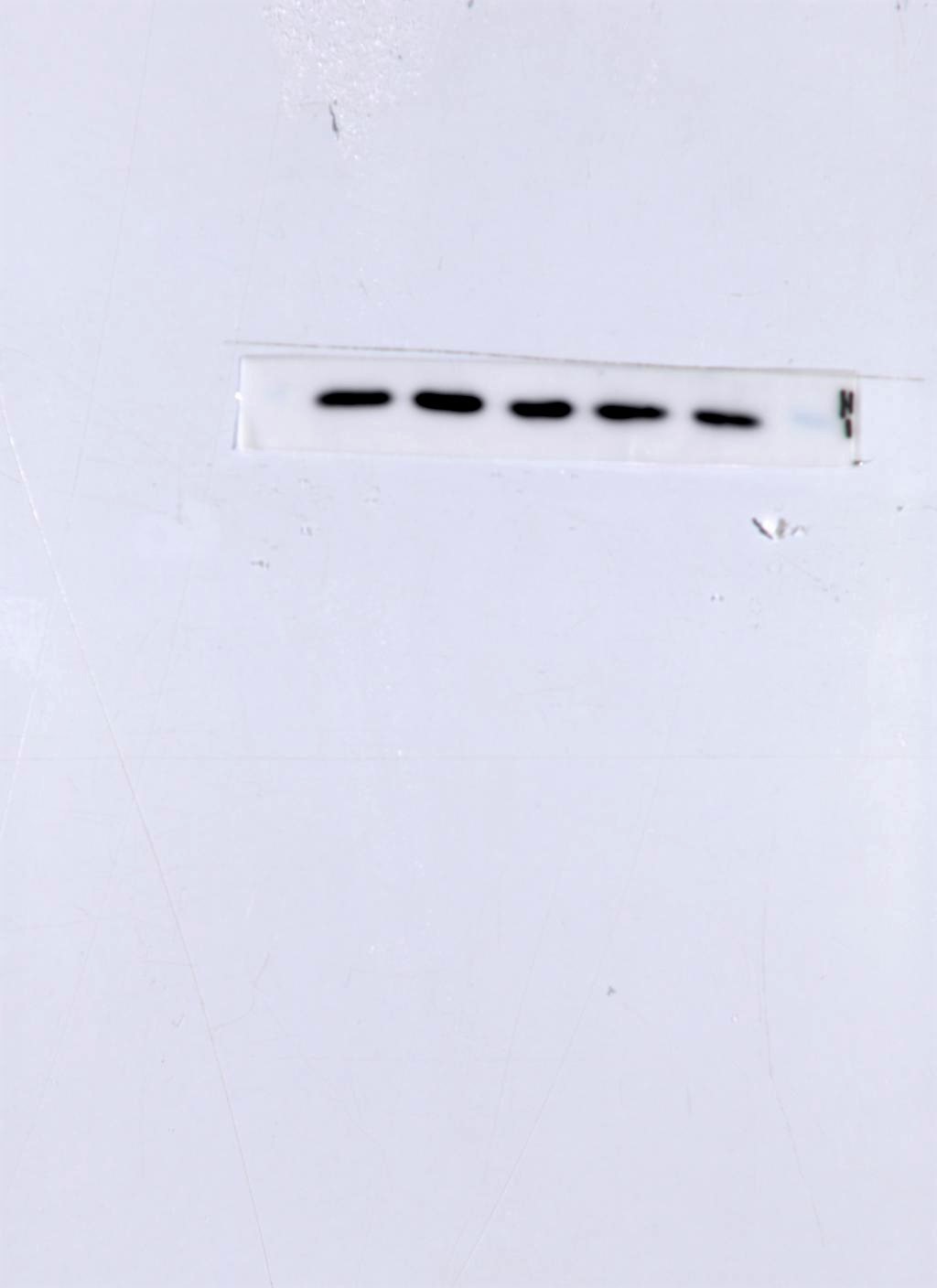

Supplement: Supplemental Information 1 [file peerj-08-8514-s001.zip › western blot/Figure3/Figure3C/BEAS-2B/HMGB1.jpg]

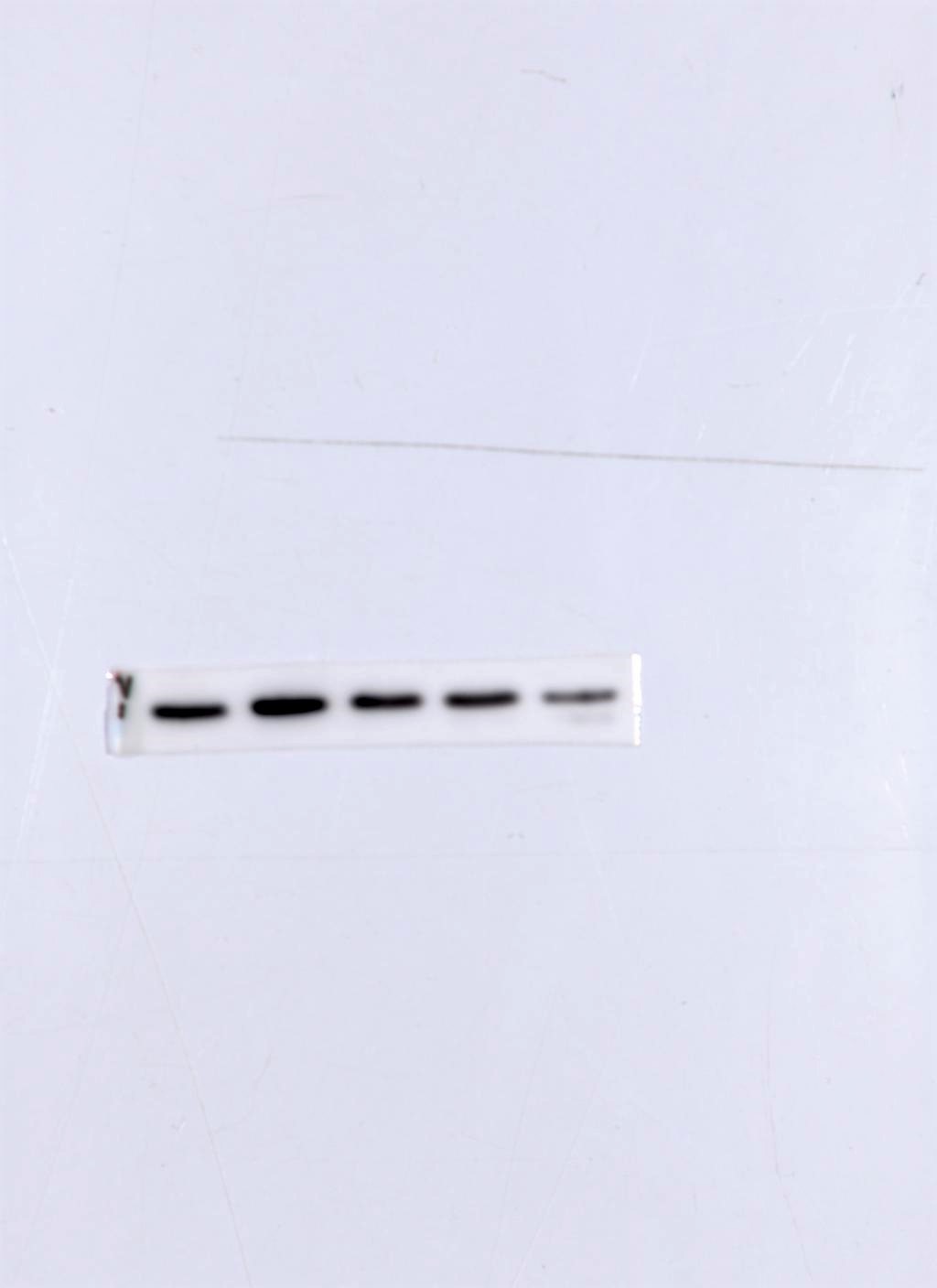

Supplement: Supplemental Information 1 [file peerj-08-8514-s001.zip › western blot/Figure3/Figure3C/BEAS-2B/Vimentin.jpg]

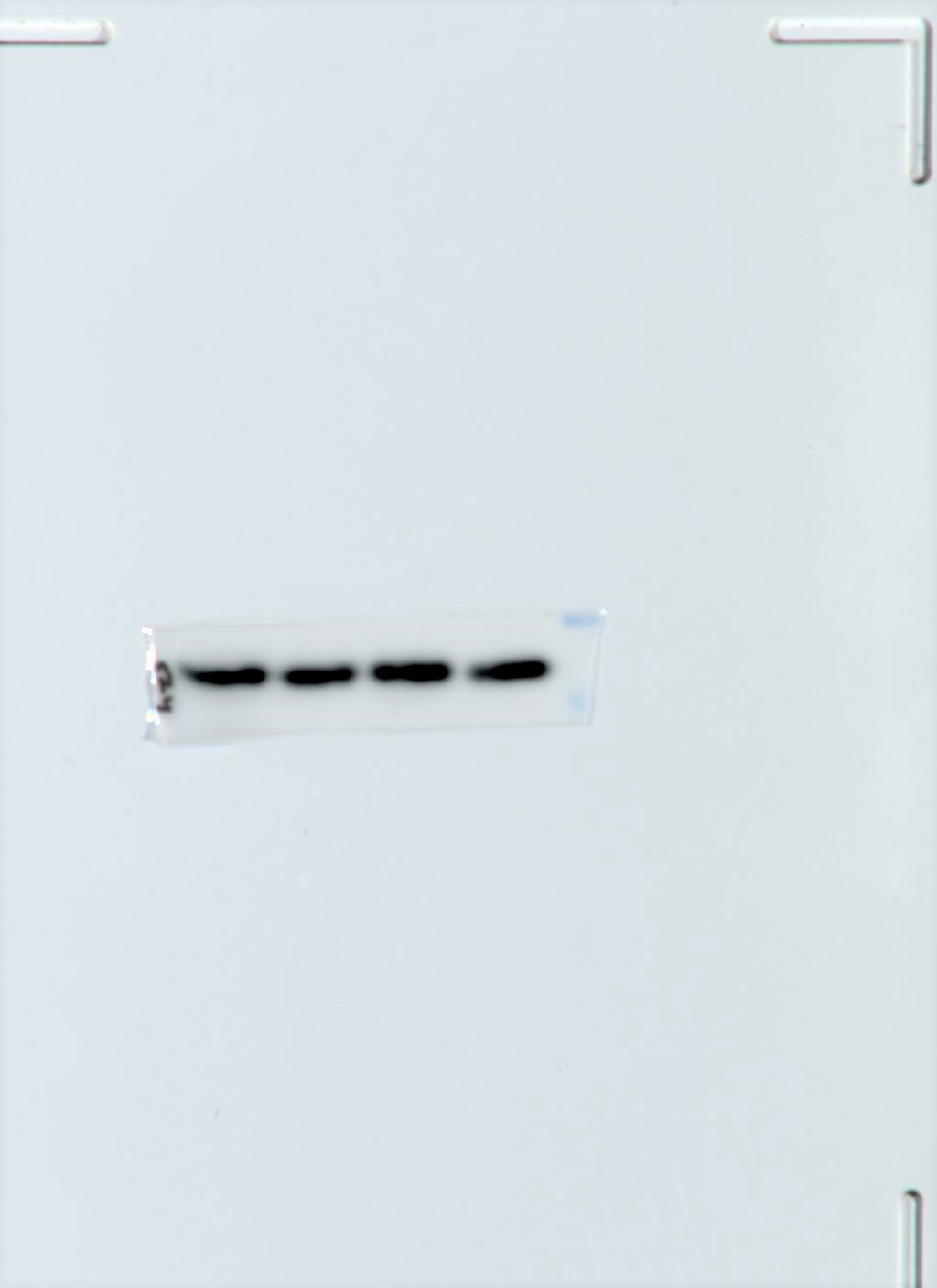

Supplement: Supplemental Information 1 [file peerj-08-8514-s001.zip › western blot/Figure4/Figure4A/A549/GAPDH.jpg]

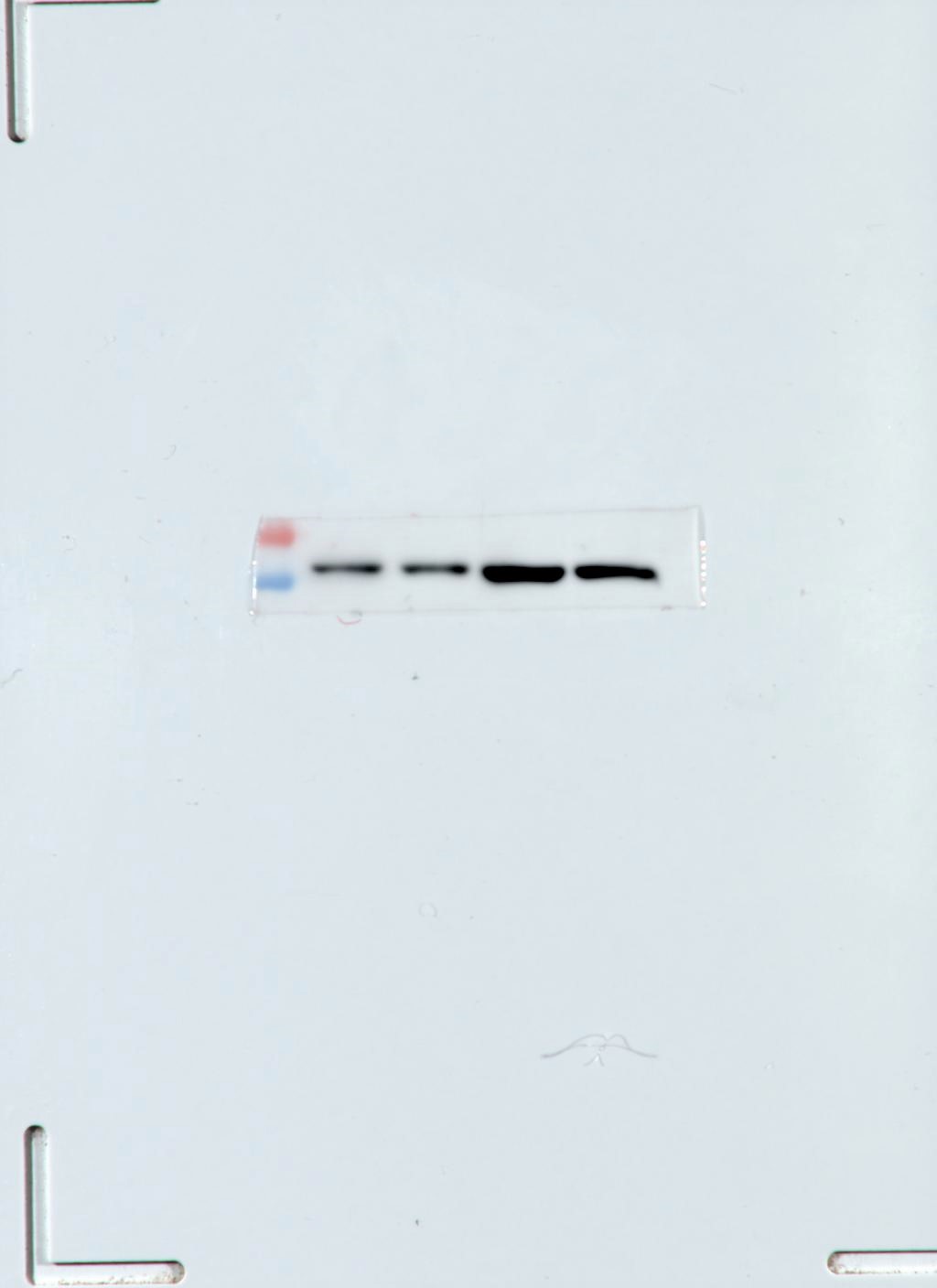

Supplement: Supplemental Information 1 [file peerj-08-8514-s001.zip › western blot/Figure4/Figure4A/A549/psmad2.jpg]

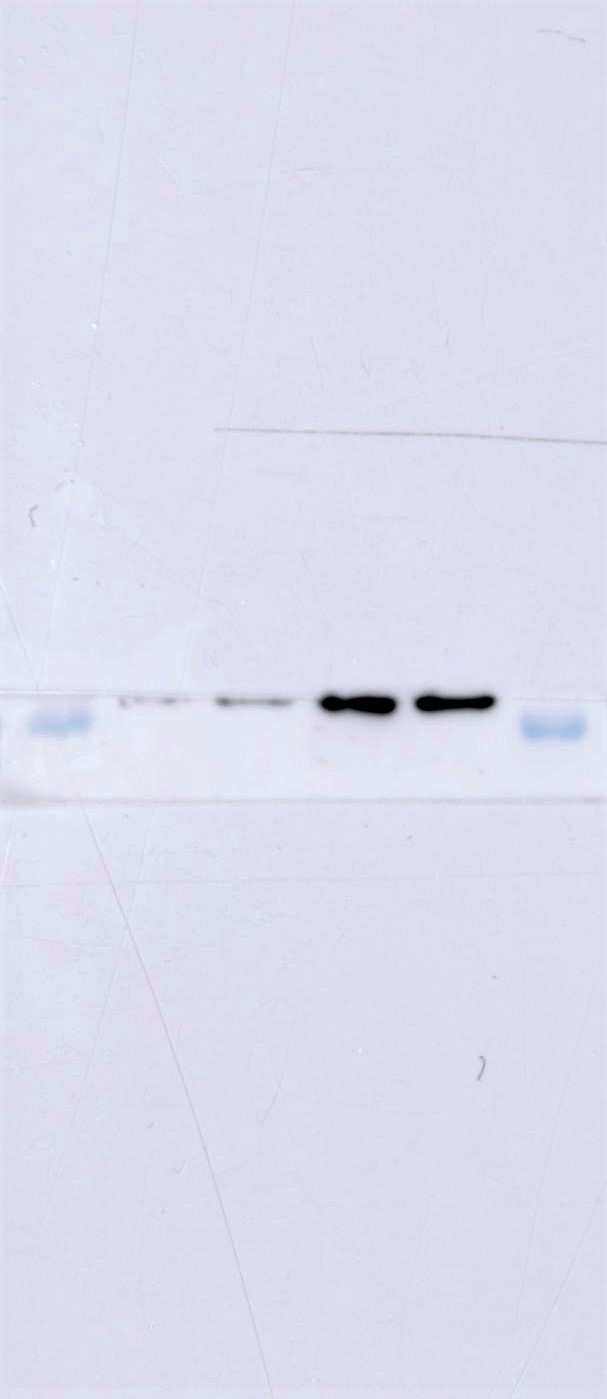

Supplement: Supplemental Information 1 [file peerj-08-8514-s001.zip › western blot/Figure4/Figure4A/A549/psmad3.jpg]

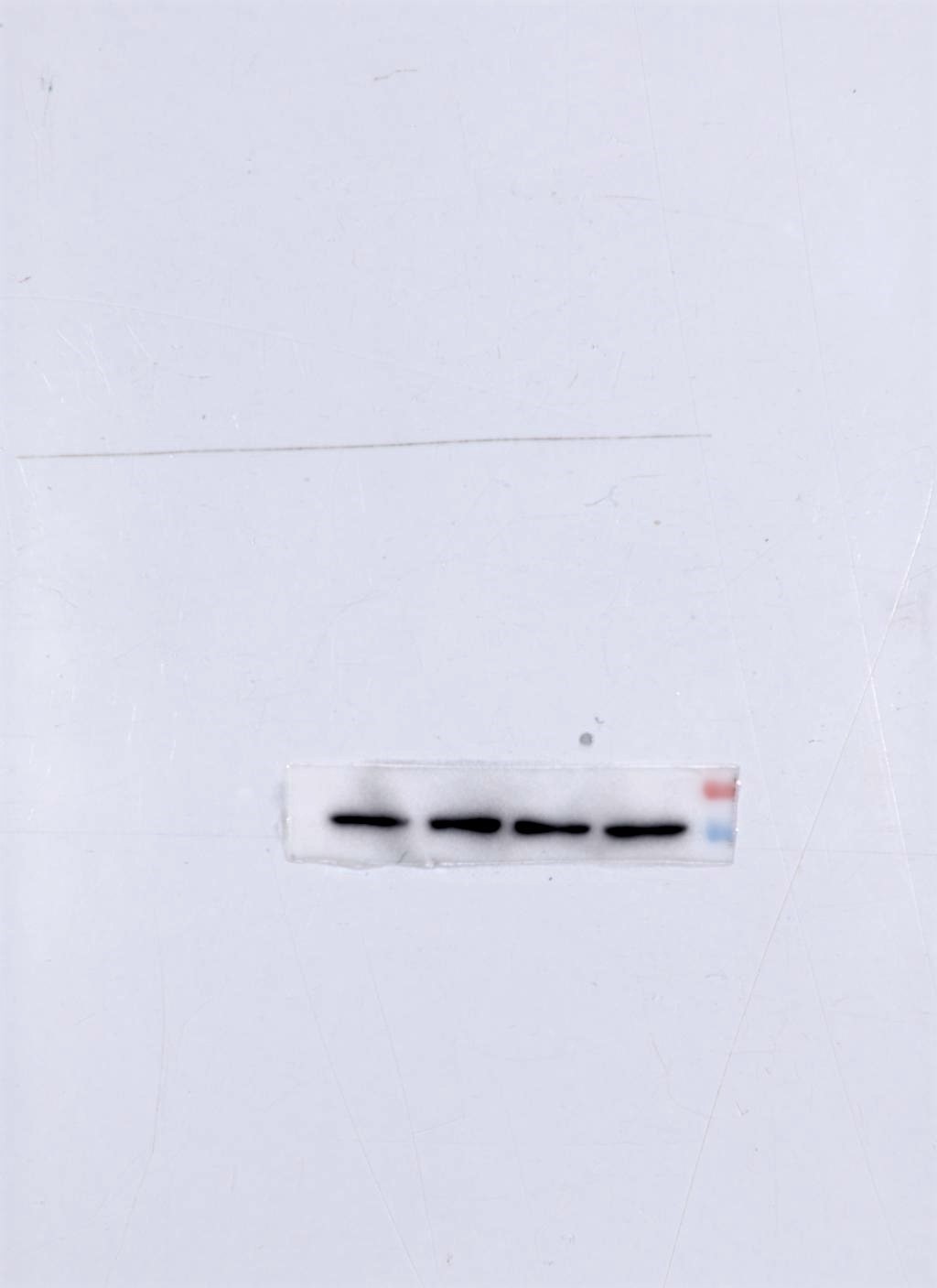

Supplement: Supplemental Information 1 [file peerj-08-8514-s001.zip › western blot/Figure4/Figure4A/A549/smad2.jpg]

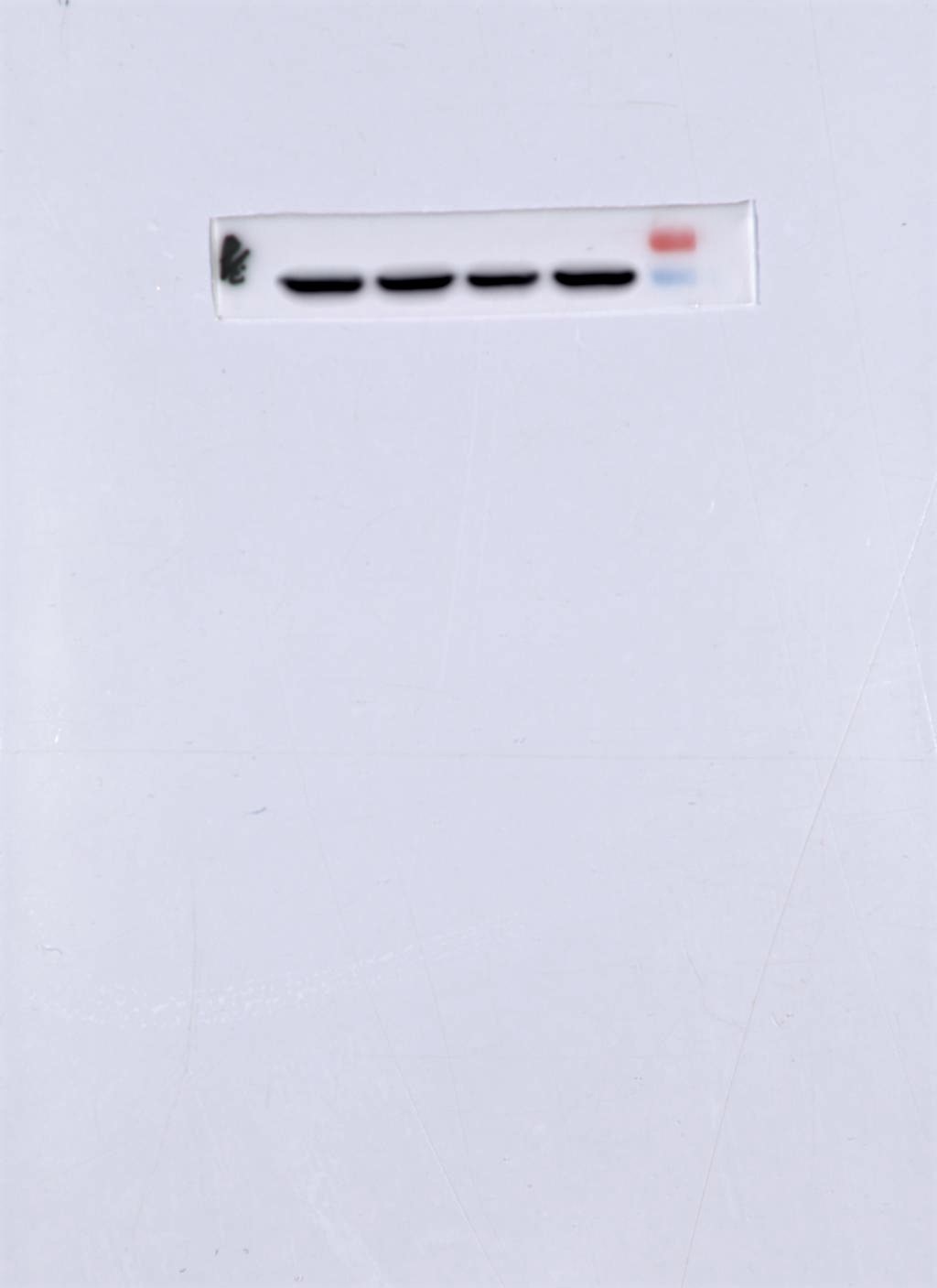

Supplement: Supplemental Information 1 [file peerj-08-8514-s001.zip › western blot/Figure4/Figure4A/A549/smad3.jpg]

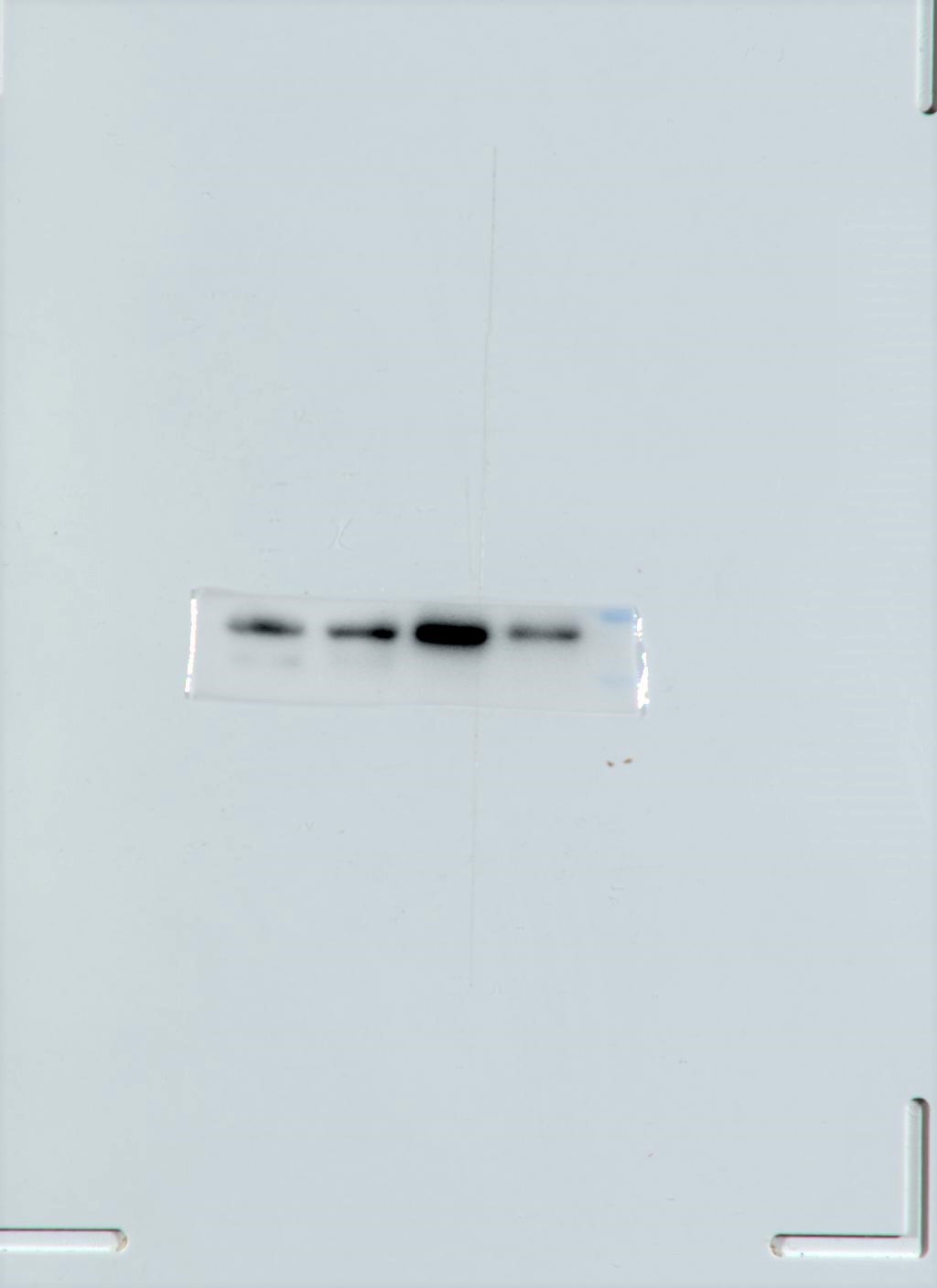

Supplement: Supplemental Information 1 [file peerj-08-8514-s001.zip › western blot/Figure4/Figure4A/A549/TGF-β1.jpg]

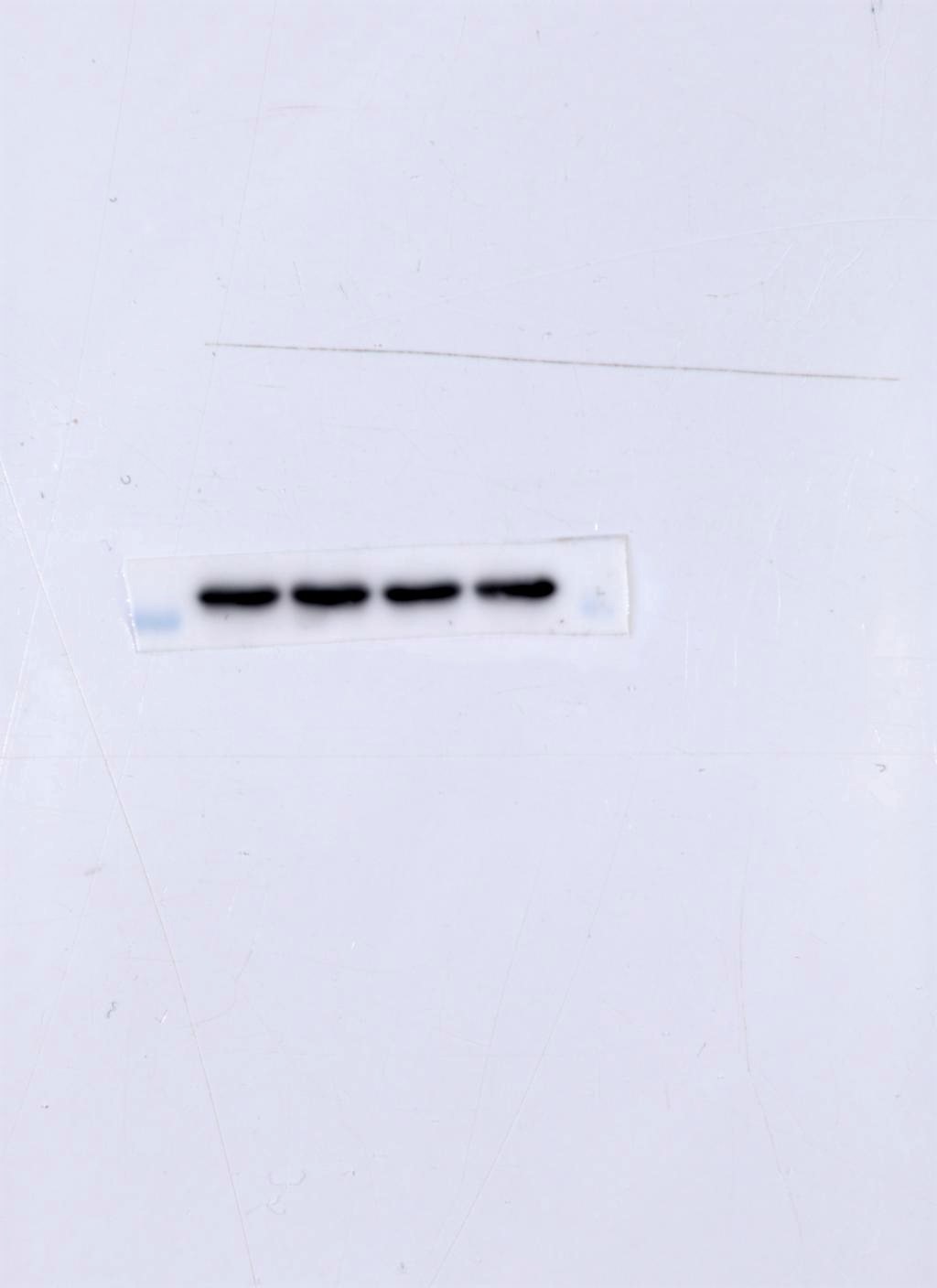

Supplement: Supplemental Information 1 [file peerj-08-8514-s001.zip › western blot/Figure4/Figure4A/BEAS-2B/GAPDH.jpg]

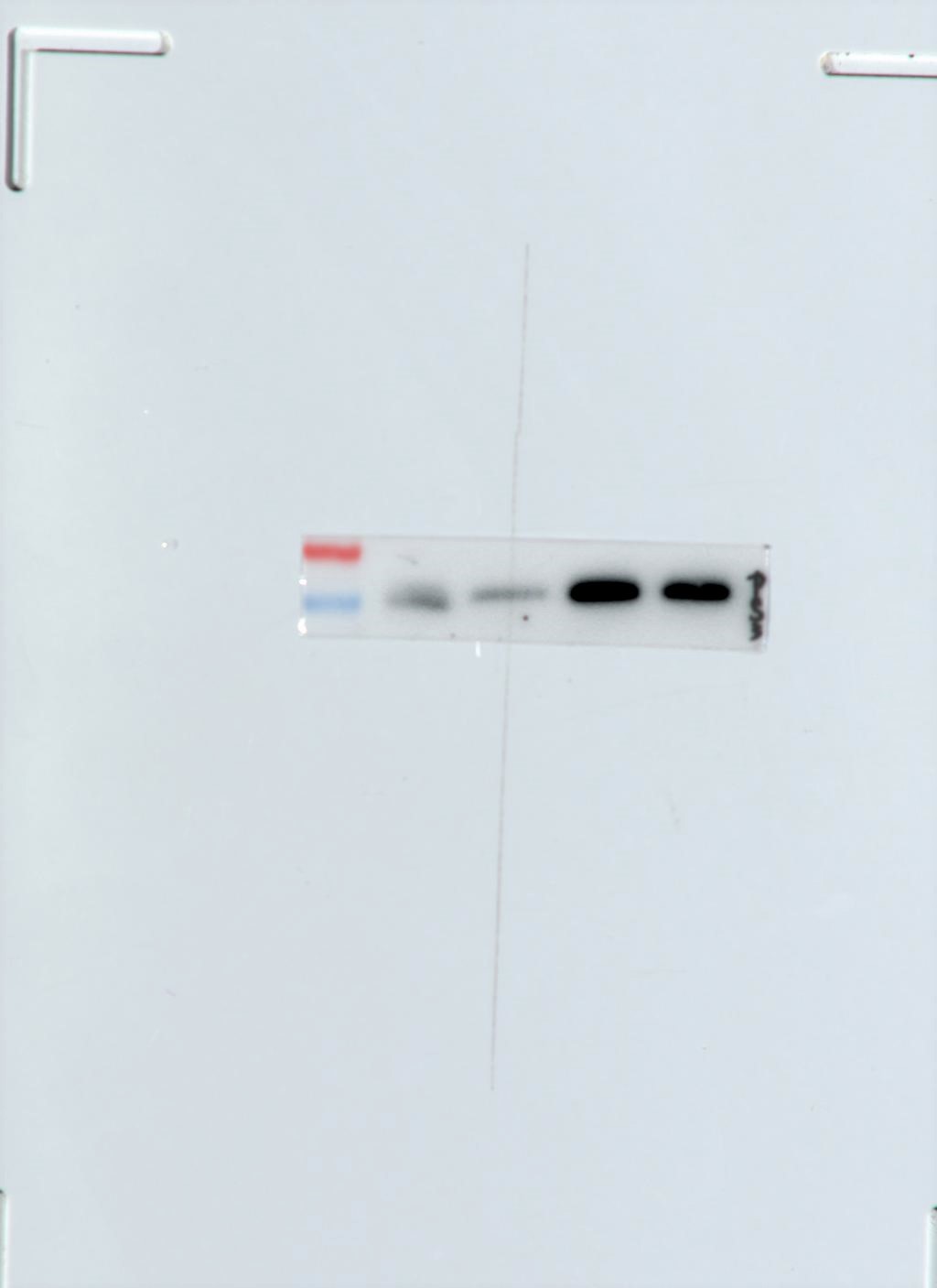

Supplement: Supplemental Information 1 [file peerj-08-8514-s001.zip › western blot/Figure4/Figure4A/BEAS-2B/psamd3.jpg]

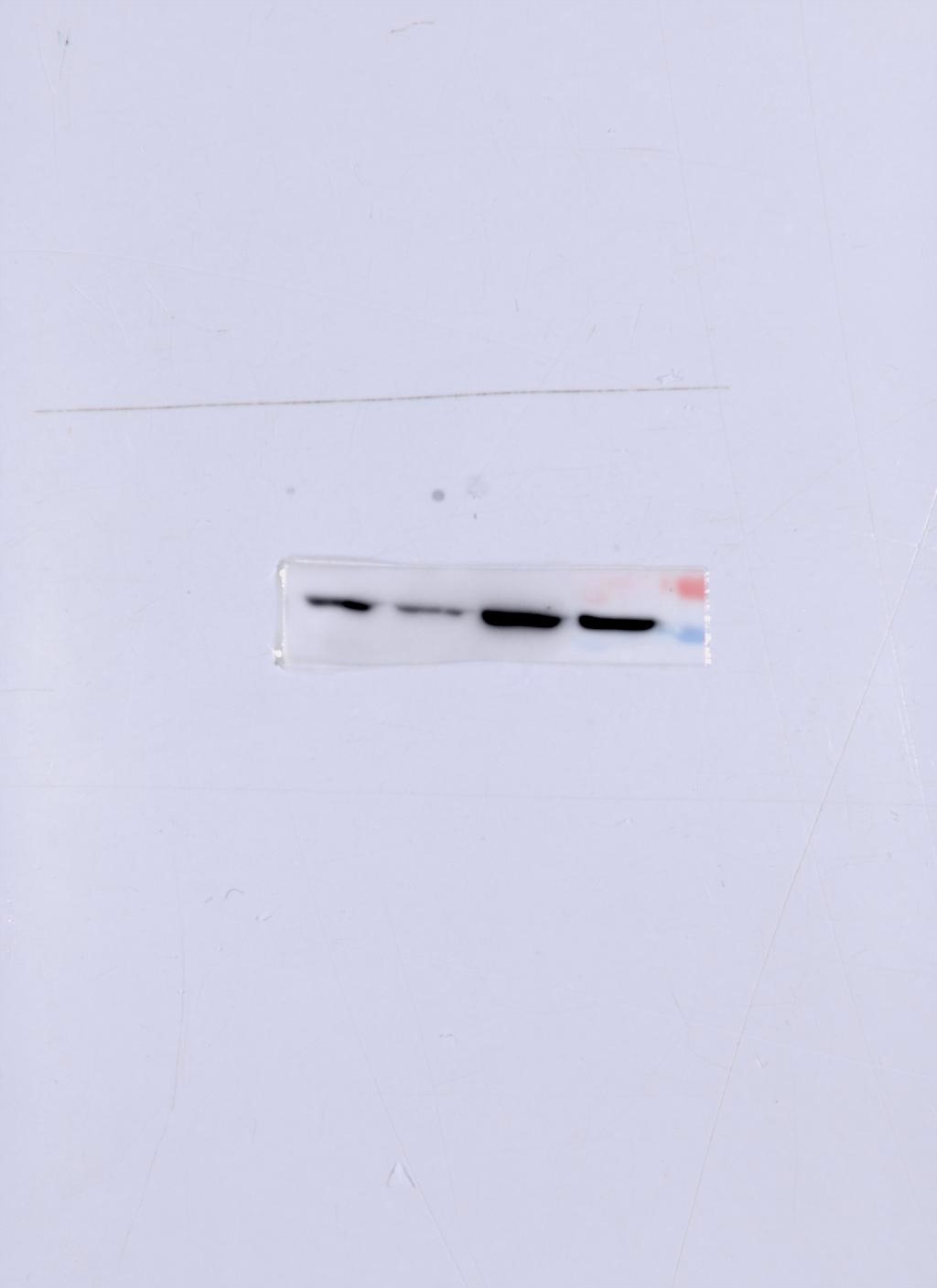

Supplement: Supplemental Information 1 [file peerj-08-8514-s001.zip › western blot/Figure4/Figure4A/BEAS-2B/psmad2.jpg]

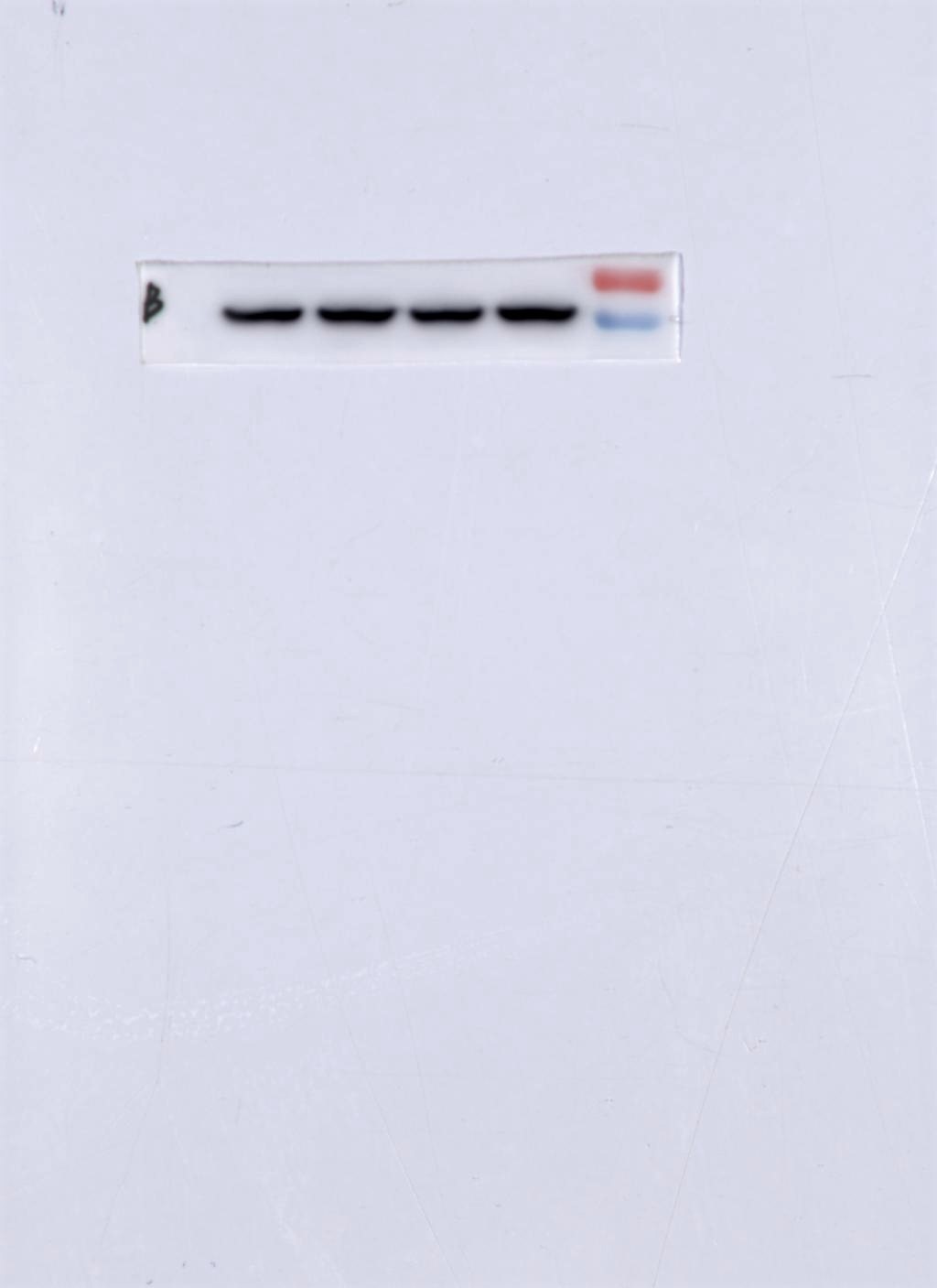

Supplement: Supplemental Information 1 [file peerj-08-8514-s001.zip › western blot/Figure4/Figure4A/BEAS-2B/smad2.jpg]

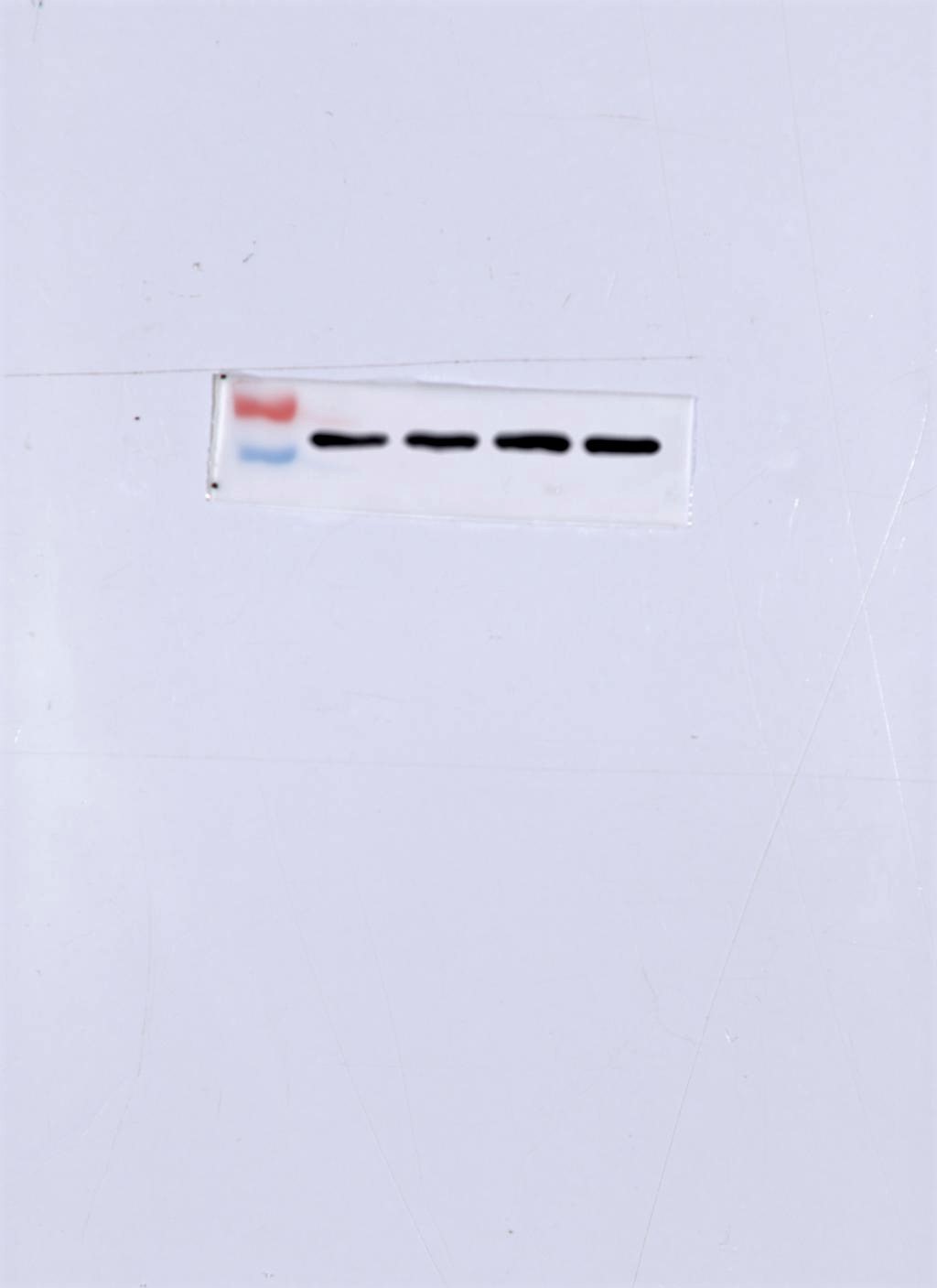

Supplement: Supplemental Information 1 [file peerj-08-8514-s001.zip › western blot/Figure4/Figure4A/BEAS-2B/smad3.jpg]

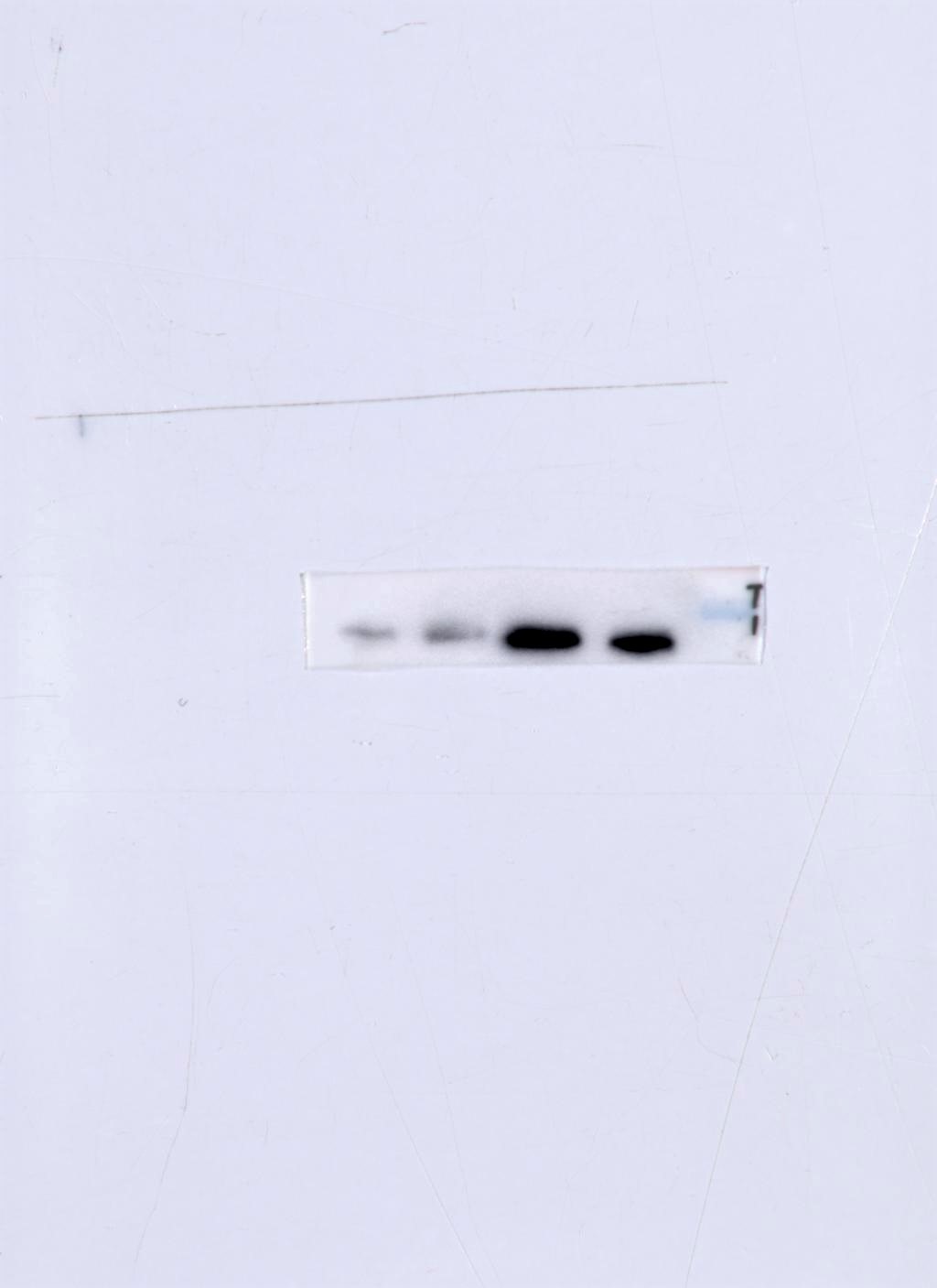

Supplement: Supplemental Information 1 [file peerj-08-8514-s001.zip › western blot/Figure4/Figure4A/BEAS-2B/TGF-β1.jpg]

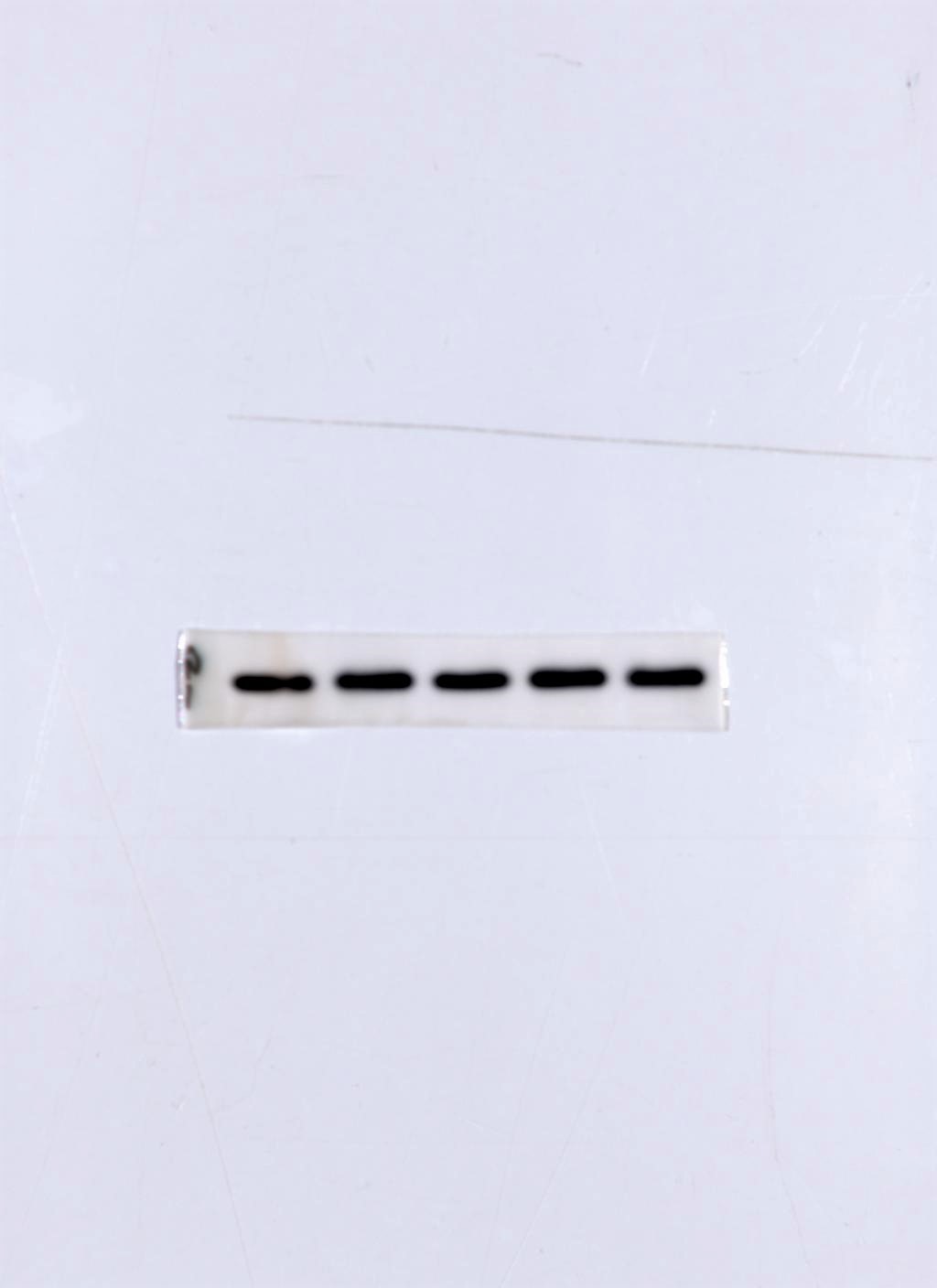

Supplement: Supplemental Information 1 [file peerj-08-8514-s001.zip › western blot/Figure4/Figure4C/A549/GAPDH.jpg]

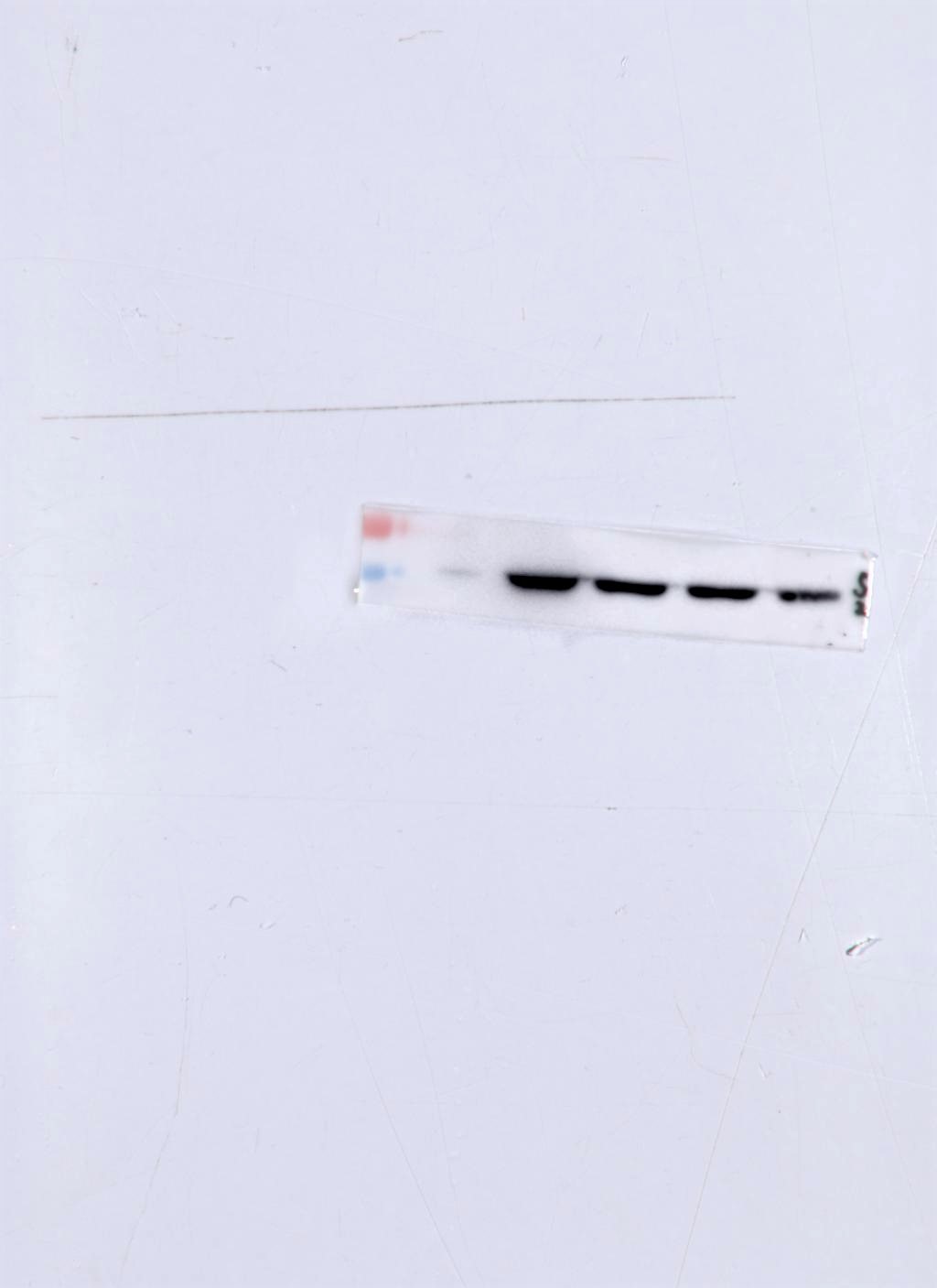

Supplement: Supplemental Information 1 [file peerj-08-8514-s001.zip › western blot/Figure4/Figure4C/A549/psmad2.jpg]

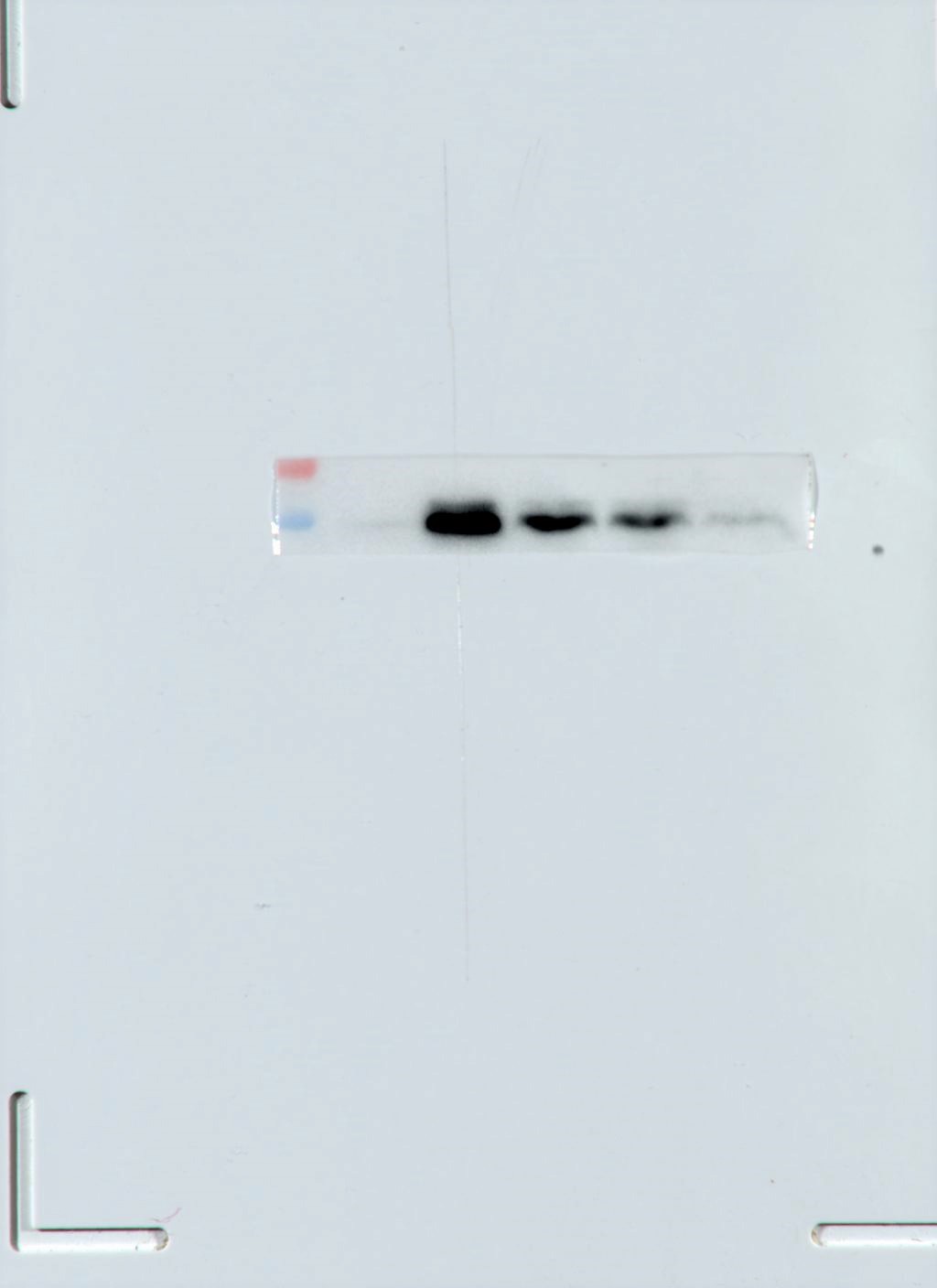

Supplement: Supplemental Information 1 [file peerj-08-8514-s001.zip › western blot/Figure4/Figure4C/A549/psmad3.jpg]

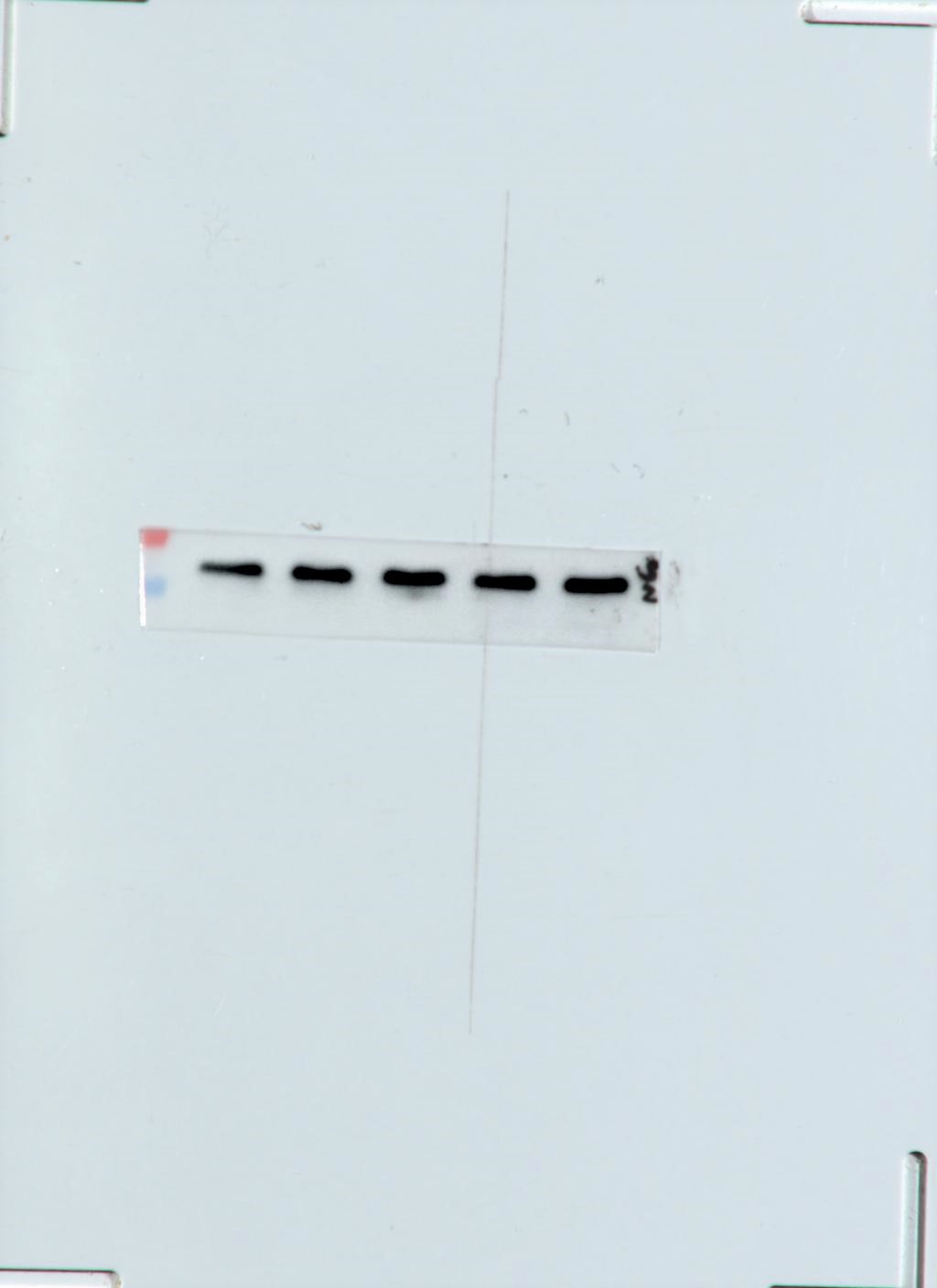

Supplement: Supplemental Information 1 [file peerj-08-8514-s001.zip › western blot/Figure4/Figure4C/A549/smad2.jpg]

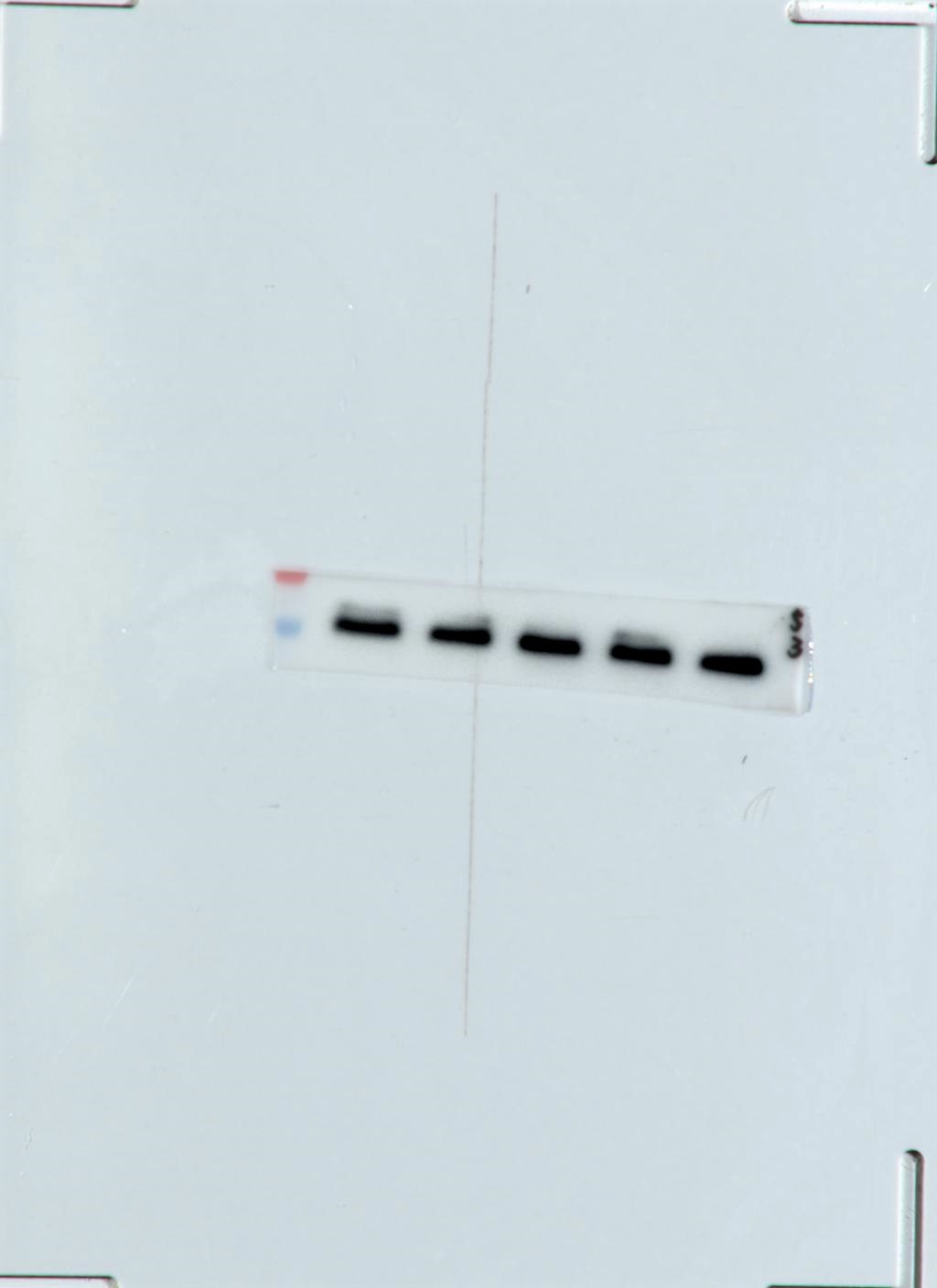

Supplement: Supplemental Information 1 [file peerj-08-8514-s001.zip › western blot/Figure4/Figure4C/A549/smad3.jpg]

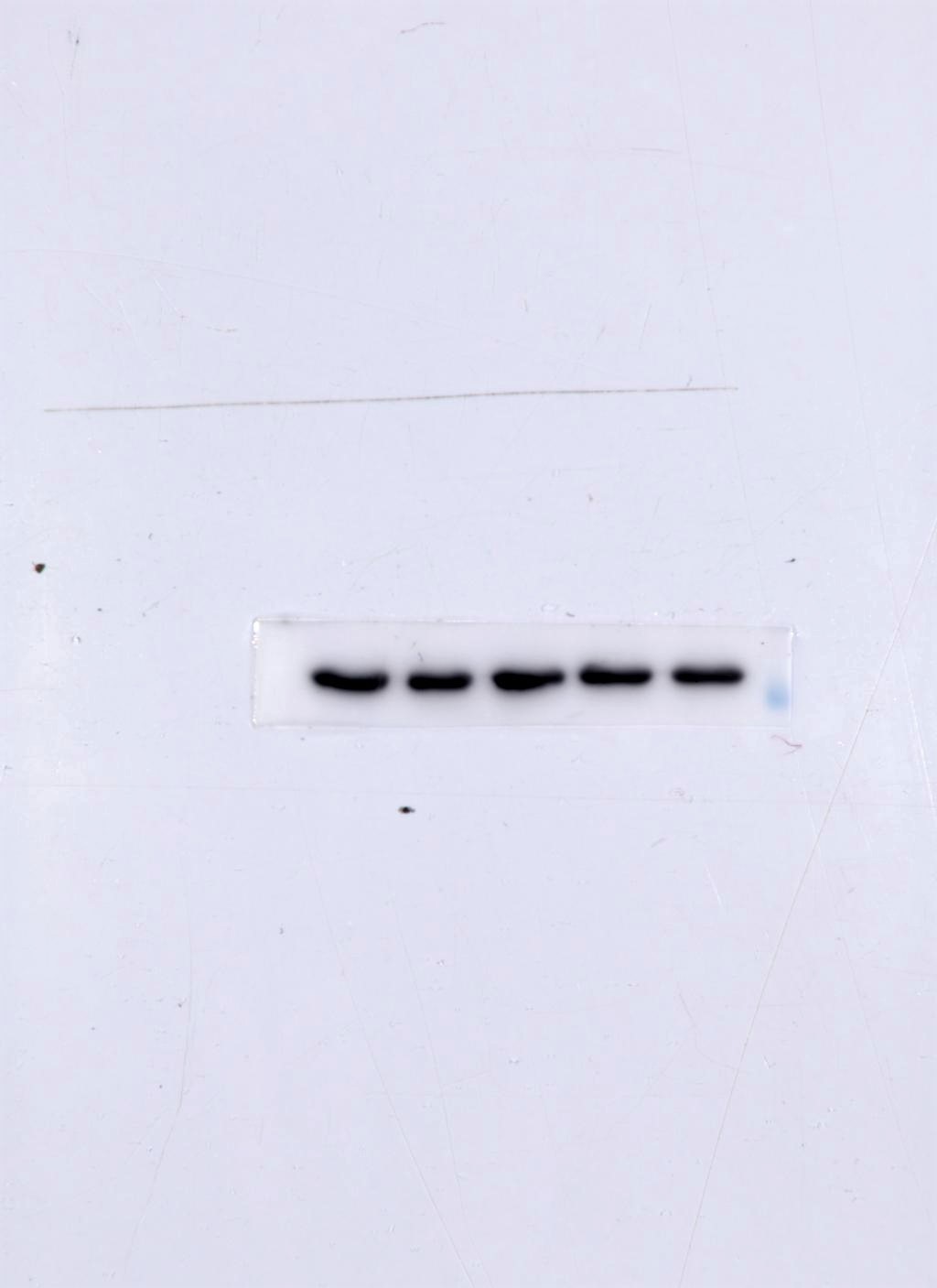

Supplement: Supplemental Information 1 [file peerj-08-8514-s001.zip › western blot/Figure4/Figure4C/BEAS-2B/GAPDH.jpg]

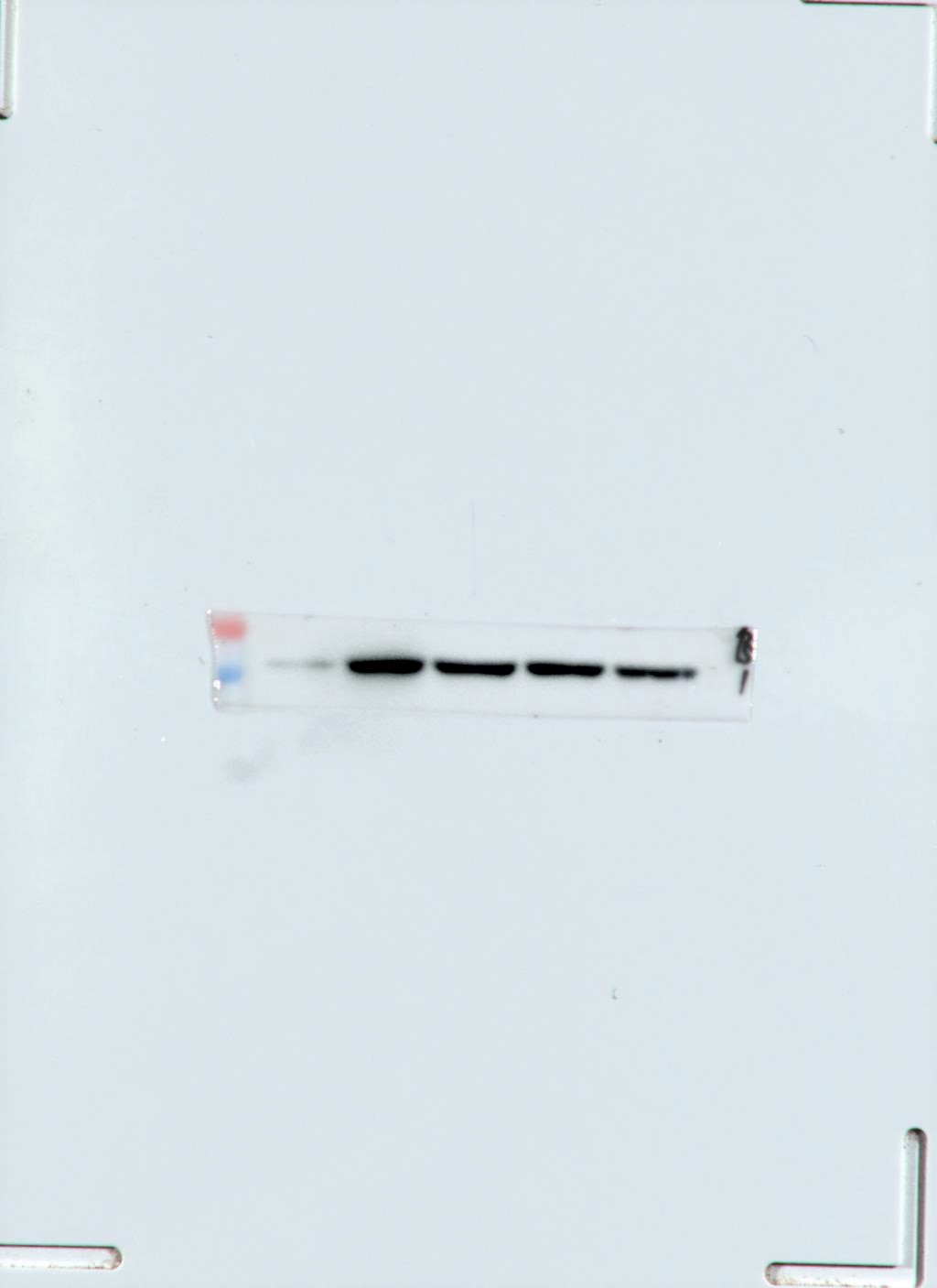

Supplement: Supplemental Information 1 [file peerj-08-8514-s001.zip › western blot/Figure4/Figure4C/BEAS-2B/psmad2.jpg]

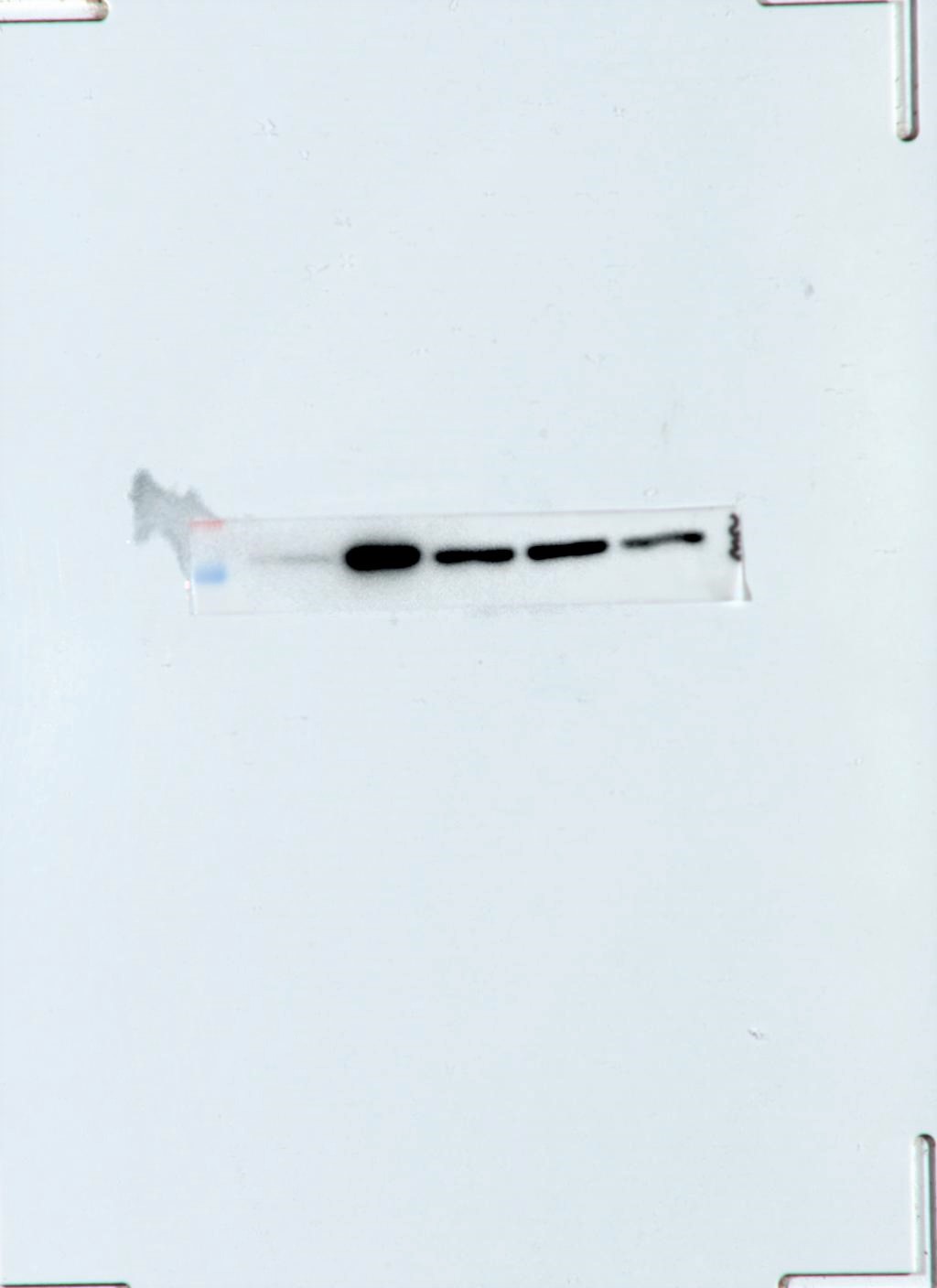

Supplement: Supplemental Information 1 [file peerj-08-8514-s001.zip › western blot/Figure4/Figure4C/BEAS-2B/psmad3.jpg]

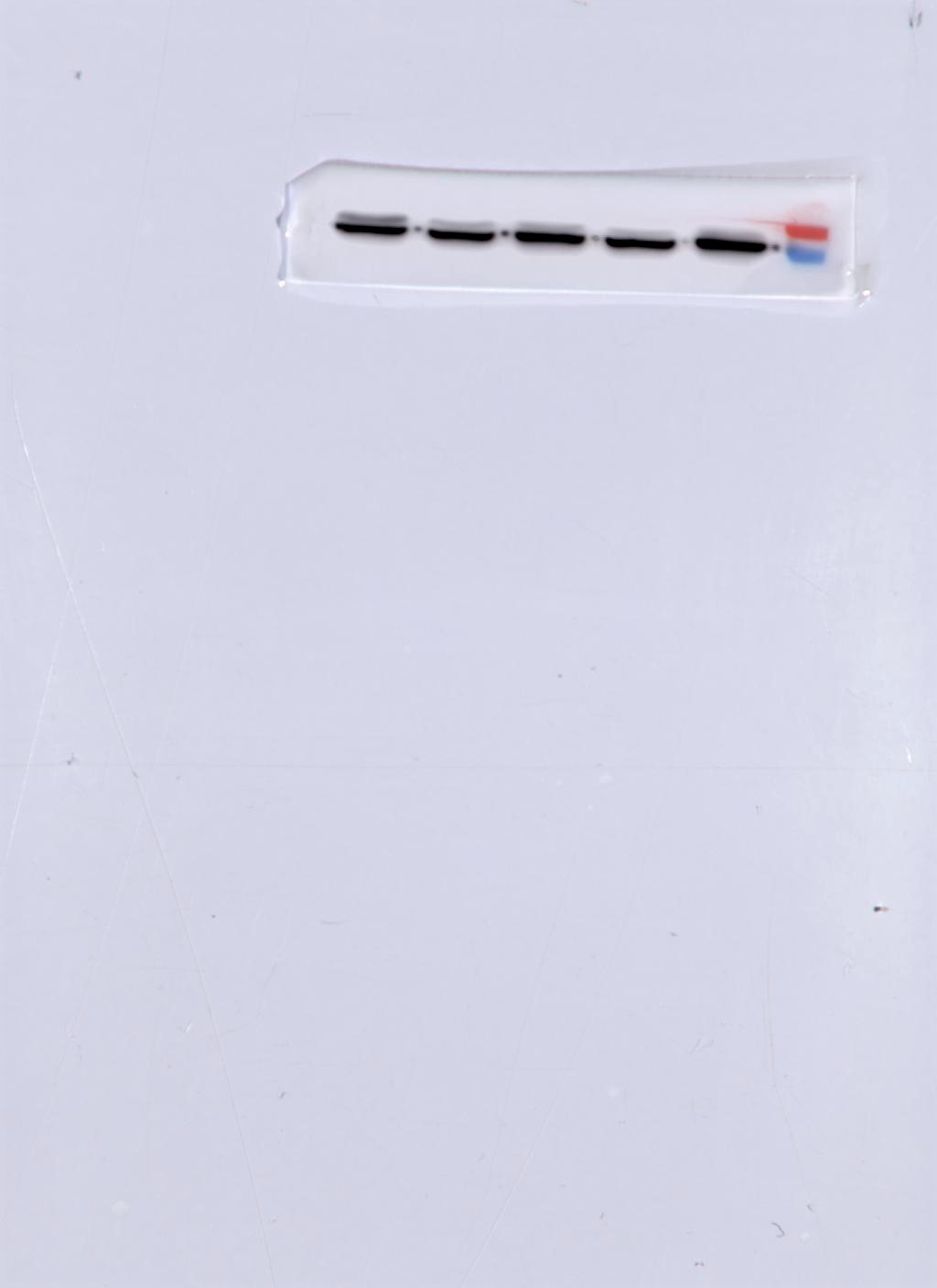

Supplement: Supplemental Information 1 [file peerj-08-8514-s001.zip › western blot/Figure4/Figure4C/BEAS-2B/smad2.jpg]

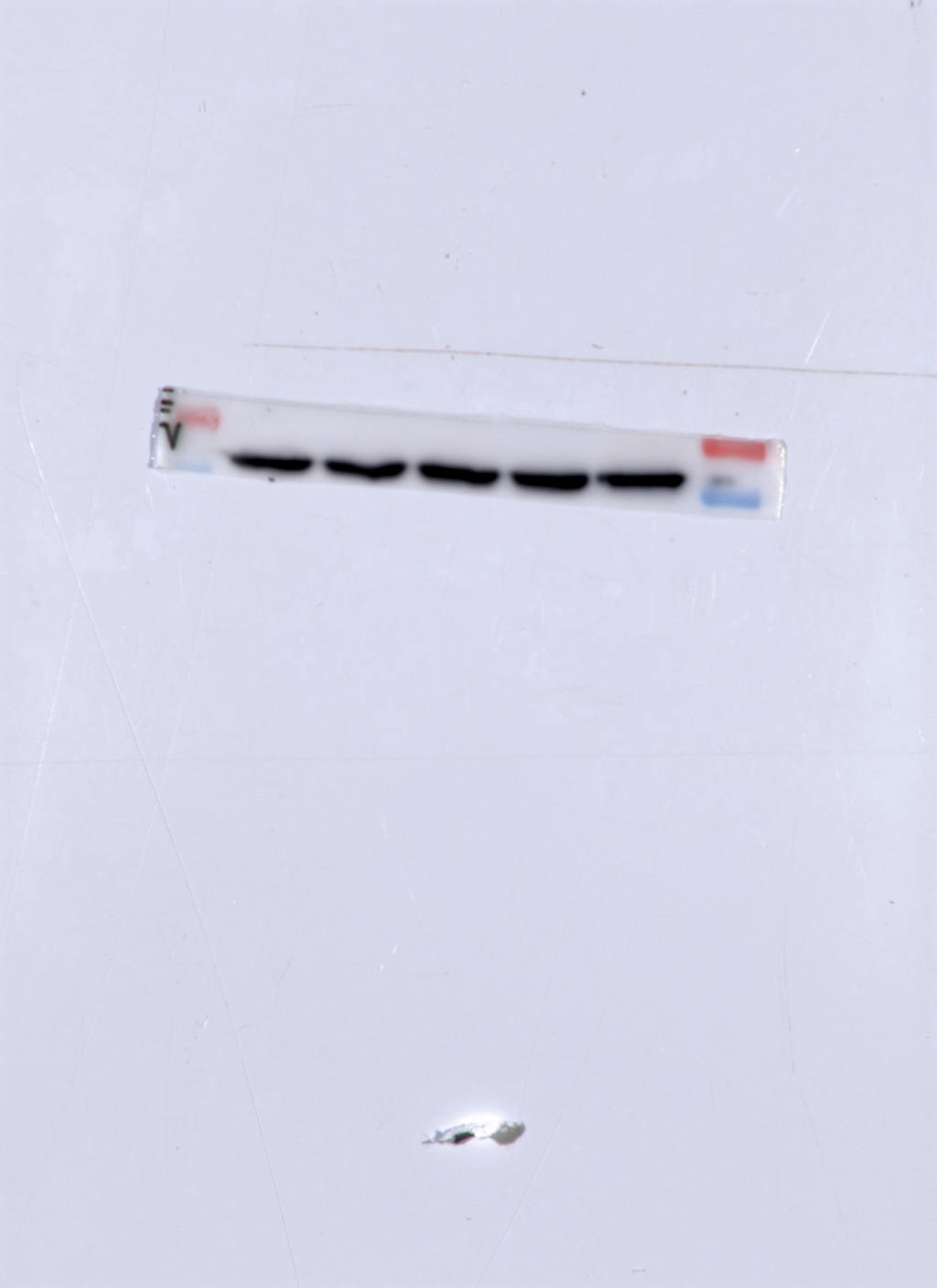

Supplement: Supplemental Information 1 [file peerj-08-8514-s001.zip › western blot/Figure4/Figure4C/BEAS-2B/smad3.jpg]
